# Organisational scale and fraud volatility: An exploratory agent-based simulation of occupational fraud dynamics

**DOI:** 10.1371/journal.pone.0342964

**Published:** 2026-07-17

**Authors:** Christopher Freeman

**Affiliations:** Centre for Cybercrime and Economic Crime, School of Criminology and Criminal Justice, University of Portsmouth, United Kingdom; Yango University, CHINA

## Abstract

Fraud is commonly studied as an individual-level phenomenon. Less attention has been given to how fraud behaves at the level of organisational systems. Empirical research on the relationship between organisational size and fraud has produced mixed findings, partly because detected-incident datasets cannot reveal the underlying generative mechanisms of fraud. This study addresses that gap by examining whether fraud scales systematically with organisational size, and whether social influence and network connectivity moderate that relationship. This study is an exploratory, conceptual agent-based simulation not empirically calibrated to real-world data. An agent-based model was developed in which employees interact within simulated organisations under varying structural and behavioural conditions. Simulations were run across nine organisational sizes (25–6,400 agents), ten levels of susceptibility to social influence (q_avg = 0 to 0.9), and five levels of network connectivity scaling (αk = 0–1), yielding 9,000 simulations in total. Log-log regression was used to estimate scaling exponents for three outcomes: mean fraud, peak fraud, and fraud volatility. Both average and peak fraud levels scale approximately linearly with organisational size. However, fraud volatility scales superlinearly (β = 1.393), meaning that a doubling of organisational size is associated with approximately 2.6 times greater variability in the level of fraud. Susceptibly to social influence and network connectivity significantly moderate the volatility of fraud activity but do not significantly affect mean fraud levels. Theoretically, these findings establish organisational scale as a structural mechanism shaping fraud dynamics and introduce fraud volatility as a distinct dimension of risk that scales disproportionately with size — one that average incidence measures fail to capture. Practically, proportional fraud control resourcing is adequate for managing average fraud levels, but managing volatility requires a different approach. Because fraud volatility is largely invisible in real organisations, the findings support reorienting fraud risk identification away from individual-centred frameworks toward affordance mapping — the systematic analysis of structural conditions that make fraud possible — as a more complete basis for directing counter-fraud effort than case-led detection alone.

## Introduction

Fraud has grown into the most commonly experienced crime in England and Wales. The most recent Crime Survey of England and Wales (CSEW) estimates 4.1 million incidents in the previous year, representing a 14% increase on the preceding period [1]. Fraud can have devastating consequences for victims, producing not only financial losses but also significant psychological and even physical harms [2,3].

While public attention often focuses on fraud against individuals, fraud committed against organisations presents a distinct challenge both empirically and epistemically. A recent UK survey reported that 27% of businesses experienced fraud within the previous year, with 74% of these reporting multiple incidents [4]. However, official reports consistently acknowledge that detected cases represent only a small fraction of total fraud activity, with the true scale of organisational fraud likely to be substantially higher [5,6]. Because most fraud remains undetected or unreported, empirical datasets provide only a partial view of the phenomenon.

These limitations create significant obstacles for research into organisational fraud. Economic crime research increasingly calls for interdisciplinary approaches that integrate criminological theory with insights from economics, organisational studies, and computational modelling [7,8]. The present study reflects this trend. It combines criminological perspectives on fraud with concepts from complexity science and agent-based modelling to examine how organisational systems generate patterns of fraudulent behaviour. Recent work has begun to frame fraud not simply as a count of detected incidents but as a dynamic system behaviour whose underlying drivers may remain partially hidden within organisational structures and interaction patterns [9].

A central unresolved question is whether fraud scales proportionally with organisational size. Larger organisations might be expected to experience more fraud due to their greater resources, transaction volumes and operational complexity. The more meaningful question, however, is whether larger organisations might face disproportionately greater fraud risk. Existing evidence is inconsistent. Surveys conducted by the Association of Certified Fraud Examiners and the UK Home Office [4,10] do not consistently show higher fraud rates in larger organisations once exposure is taken into account. By contrast, Barnes and Webb [11] found that both fraud susceptibility and individual fraud loss magnitudes increase with organisational scale. This inconsistency is difficult to resolve using observational data, because detected-incident datasets conflate the underlying prevalence of fraud with the effectiveness of detection mechanisms [12]. Larger organisations may simply detect fraud more readily, making their reported rates an unreliable guide to actual exposure.

These limitations motivate the three research questions addressed by this study:

1. Does fraud in organisations scale proportionately with organisational size?
2. Do variations in network connectivity and susceptibility to social influence shape fraud dynamics?
3. How does organisational scale influence the volatility of fraud activity over time?

Resolving these questions from observational data alone is not feasible. Detection limitations mean that empirical datasets systematically undercount fraud and conflate prevalence with detection effectiveness. Individual-level frameworks also tend to overlook systemic properties that only become visible when organisational structure and interaction dynamics are explicitly modelled. Agent-based modelling offers an alternative, allowing hypothesised generative mechanisms to be specified and observed within simulated organisational systems, without dependence on detected-incident data. Building on the researcher’s previous argument that fraud may be understood as an emergent property of a complex adaptive system [13], this study models how organisational scale interacts with two system-level mechanisms: social influence among employees and the structural connectivity of organisational networks.

This framing draws on evidence that complex systems—biological, urban and organisational—commonly exhibit non-linear scaling relationships in which outcomes increase either more slowly or more rapidly than system size [14–16]. Whether fraud follows similar scaling dynamics within organisational systems has received little systematic investigation. Economic crime research has increasingly called for interdisciplinary approaches that integrate criminological theory with computational modelling [7,8], but the relationship between organisational scale and fraud dynamics remains largely unexplored from this perspective [9].

Consistent with this exploratory modelling approach, the study does not test formally specified hypotheses. Instead, it examines how theoretically grounded mechanisms relating to organisational scale, network connectivity and social influence may generate observable patterns in fraud outcomes. In particular, the analysis explores whether these mechanisms are capable of producing non-linear scaling relationships and varying degrees of stability and volatility in fraud dynamics.

There is currently no systematic simulation-based investigation of how fraud scales with organisational size. This study addresses that gap using an agent-based model that represents employees interacting within organisational networks of varying size and structure, run across 9,000 simulations spanning organisations of 25–6,400 agents. The study makes three contributions: it demonstrates that fraud volatility scales superlinearly with organisational size while mean fraud scales approximately proportionally; it extends prior agent-based models of occupational fraud by treating organisational size and network connectivity as primary design variables; and it shows that complexity-oriented simulation can reveal generative fraud mechanisms that remain hidden in empirical detected-incident data.

### Literature Review

Empirical data confirm that fraud remains a significant and widespread problem for organisations. A recent UK survey found that 71% of businesses reported a direct or indirect financial loss due to fraud, with 27% experiencing fraud in the previous year and 74% of those reporting multiple incidents [4]. The biannual report by the Association of Certified Fraud Examiners [10], drawing on 1,921 occupational fraud cases worldwide, found that 21% of victim organisations had fewer than 100 employees, 22% had 100–999 employees, 31% had 1,000–9,999 employees, and organisations of more than 10,000 employees accounted for the remaining 26%. In the UK, 37% of businesses with a turnover exceeding £1 million reported fraud compared with only 27% below that threshold [4]. These figures suggest that the relationship between organisational scale and fraud incidence is unlikely to be simply proportional.

Barnes and Webb [11] analysed survey data from UK organisations to test whether organisational size affects susceptibility to theft and fraud. Their findings indicated that larger organisations reported higher susceptibility to fraud and that the monetary size of individual fraud losses increases disproportionately with organisational size. They attribute this to structural features of scale: greater access to resources, increased transaction volumes, and organisational complexity. Interestingly, their analysis also finds that variation in management control configuration does not significantly affect overall susceptibility [11]. This suggests that structural properties may exert a stronger influence on fraud exposure than the specific design of control systems alone.

Taken together, however, the evidence across studies is inconsistent. ACFE and UK Home Office surveys do not consistently show higher fraud rates in larger organisations once exposure is controlled for [4,10], while Barnes and Webb [11] find the opposite. This inconsistency is difficult to resolve using only observational data. All three datasets rely on detected fraud and therefore capture only the observable portion of total organisational fraud activity [17]. Larger organisations may detect fraud more readily, meaning that higher reported rates may reflect detection effectiveness rather than underlying prevalence [12]. Empirical methods alone are therefore insufficient to determine whether fraud scales proportionally, sublinearly, or superlinearly with organisational size.

### Individualistic accounts: the fraud triangle and its critiques

While Sutherland’s [18,19] seminal work has an enduring influence on the study of white-collar crime, there is a need for precision in discussion of the topic. White-collar crime can be divided into ‘corporate crime’, this being acts committed by those within an organisation for the benefit of the organisation, and ‘occupational crime’, acts against the organisation’s interests for personal benefit, such as fraud or embezzlement [20]. This study is concerned with the latter.

Scholarly explanations of occupational fraud remain dominated by the fraud triangle paradigm, which portrays fraud as a consequence of personal motives, opportunities and rationalisations. At least nineteen such theories trace their lineage to Donald Cressey’s mid-twentieth century study of embezzlers [21–23], although later theories can exhibit misinterpretation or misrepresentation of Cressey’s work [23,24], and Cressey himself did not use the term ‘fraud triangle’ [25]. Other scholars and professionals have extended the triangle by including additional psychological, sociological or situational factors [e.g., 26–28]. The fraud triangle has become ubiquitous in both scholarly and professional literature on fraud. It has attained paradigmatic status in the Kuhnian sense: research focus remains anchored to positioning fraud as a psychological or moral problem of the individual [23], and this tradition has been highly influential in professional practice, including accounting, fraud examination and auditing [24,29].

This body of work is directly relevant to the present study, as the model adopts the core decision structure derived from this tradition. However, the fraud triangle and its variants largely overlook the significance of factors outside the individual, specifically the wider system in which the individual is situated and how that system both shapes and is shaped by individual actions. The problem of fraud requires analysis through both psychological and social lenses, since “[s]ocial structures powerfully nourish or retard individual malfeasance” [20].

A partial response to this critique can be found in a situational action theory-based model of fraud that proposes [30]:


$$\begin{array}{c} ``\text{Fraud }=\text{ Propensity for Fraud }\times \text{ Exposure to Criminogenic Environment}" \end{array}$$

Here, the propensity to commit fraud is a matter of individual psychology, while the criminogeneity of the environment is shaped by its moral norms, culture, workforce dynamics and tolerance of unethical behaviours. This model is a significant step in recognising structural influences on fraud. However, it still seeks to explain the behaviour of the individual rather than the aggregate dynamics of the system.

Bunge goes further, calling for a ‘systemic perspective on crime’ that advocates a multi-level account incorporating both individualist and holist perspectives: “…an individual’s actions cannot be understood without considering the systems of which he is a part, just as these cannot be understood except as being composed of individuals who maintain, reinforce, or weaken the bonds that keep them and others in their systems” [31]. This points toward analytical approaches capable of capturing interaction, feedback and system-level dynamics.

### Fraud as a systemic and complex problem

There are limited but notable examples of research into fraud that adopt a systemic perspective. Button et al. [5] developed a ‘fraud field’ model as a conceptual framework for explaining the opposing forces of threats and safeguards that together determine fraud levels. The researcher subsequently updated Fraud Field Theory using a complexity-theory-inspired approach [13], making opportunities the central element shaped by the interaction of threat and safeguard fields. This frames the fraud field as a complex adaptive system in constant flux, with fraud levels capable of exhibiting non-linear behaviour.

Similar critiques have emerged in adjacent domains where agent-based modelling has been used to examine how risk develops within complex social systems [32]. Rather than treating risk factors as independent and additive, such studies show how outcomes emerge through interaction over time, often producing non-linear and path-dependent dynamics. Risk, from this perspective, is a property of the system rather than a static attribute of its members.

The meaningful analysis of complex social systems requires attention to interactions between elements rather than properties of those elements in isolation [33,34]. Complex systems feature large numbers of dynamically interacting elements exchanging energy or information, governed by feedback loops and non-linearities [33,35]. Agents interact according to rules that determine responses to stimuli, and these rules are updated as agents learn from experience [36].

The effects of scale on system behaviour are well established in urban contexts. Research on cities challenges the assumption that per capita indicators scale linearly with population [e.g., 14, 16, 37]. Crime rates are no exception. Banerjee et al. [38] showed that while the number of criminals scales linearly with population, law enforcement capacity grows sublinearly. Beyond a threshold, law enforcement is outpaced and crime rates become superlinear. These findings suggest that system scale can fundamentally alter the balance between harmful activities and the mechanisms intended to constrain them. As organisations grow, increasing numbers of employees, transactions and interaction pathways may create conditions in which fraudulent behaviour can propagate in a similarly non-linear fashion.

Empirical research supports the idea that misconduct propagates through social interaction within organisations. Robinson and O’Leary-Kelly [39] found that individuals’ antisocial behaviour was positively associated with coworkers’ behaviour across 35 work groups. Dimant [40] demonstrated through experimentation that antisocial behaviour spreads through peer networks and that social proximity amplifies contagion effects. More recently, Mitsuhashi et al. [41] found that hospital nurses became more likely to violate rules when exposed to peer rule-breaking during shared shifts. These studies lend empirical support to modelling approaches that incorporate social influence as a driver of behavioural diffusion. Crucially, however, none of these studies examines whether organisational scale moderates these contagion dynamics.

### Agent-based modelling in criminology and economic crime

A systems-based understanding of fraud has begun to be operationalised through agent-based models (ABM). Although ABMs are increasingly used in criminological study, enabling the modelling of complex dynamic systems in which outcomes arise from agent interactions [42], their application to organisational fraud remains limited.

Kim and Xiao [43] used an ABM to examine statistical and spatial crime patterns arising in a welfare programme. Agents were either ‘recipients’ (N = 1,000) or ‘vendors’ (N = 20). Each had opportunities and propensity to engage in fraudulent exchanges. When the simulation outputs were compared to empirical data from a real programme, they showed good alignment. The model’s limitations were, however, well acknowledged.

Davis and Pesch [44] developed the most directly relevant ABM. They constructed a model of occupational fraud within an organisation to test the effectiveness of various fraud prevention techniques. The model comprised 100 agents (i.e., simulated employees) whose interactions were governed by rules derived from Cressey’s characterisation of occupational fraud, justified on grounds of its dominance in the professional literature and its simplicity.

The model’s rules determined that “…any agent in our model possessing motive, opportunity, and an attitude that frames the fraudulent act as acceptable will commit fraud” [44]. Each agent’s attitude to fraud was influenced by those in their local network, with q representing the probability of emulating others’ behaviour. At moderate or high values of q (> 0.3), significant swings in the number of agents engaged in fraud were observed. Where q < 0.3, fraud settled into a steady level. The transition was gradual rather than bifurcated.

Davis and Pesch’s contribution is invaluable. However, their modelling was constrained by the computing time required (around 5,000 hours), which led them to keep the model at 100 agents, noting that they “…felt that the additional insight provided was likely to be limited” [44]. As a result, they did not consider whether fraud dynamics might differ at other organisational sizes. The same limitation applies to Kim and Xiao [43], who substantially downscaled the real Ohio welfare system without treating population size as a variable of theoretical interest. Across both studies, the size of the agent population is fixed within the modelling design and never examined as a generative mechanism in its own right.

This gap is the departure point for the present study. Organisational size has not been examined as a primary design variable in ABM-based fraud research, despite evidence from complexity science that scale can fundamentally alter system dynamics. The present study addresses this directly.

### Theoretical Expectations

The literature reviewed above generates three theoretical expectations that guide the simulation design and the interpretation of results. These are not formal hypotheses, but theoretically grounded conjectures derived from complexity science and prior ABM research. Their purpose is to provide an explicit basis against which the simulation outputs can be assessed.

*Expectation 1:* Mean fraud levels will increase superlinearly with organisational size. Urban scaling research shows that harmful social outcomes often increase faster than population size [14,38]. Davis and Pesch’s [44] model demonstrates that social influence can generate non-linear fraud dynamics within a fixed-size organisation. Combining these insights, as organisations grow the expanding network of agent interactions should amplify fraud beyond simple proportionality.

*Expectation 2:* Fraud volatility will increase faster than mean fraud levels. In complex systems, growth in scale tends to amplify instability as well as average outcomes. As the number of interaction pathways through which pro-fraud attitudes can propagate increases disproportionately with size, fluctuations in fraud activity should become more pronounced. Volatility is therefore expected to scale more steeply than mean fraud.

*Expectation 3:* Network connectivity and susceptibility to social influence will moderate the scale–fraud relationship. Davis and Pesch [44] show that the parameter q governs whether fraud dynamics are stable or volatile at a fixed organisational size. In larger organisations with denser interaction networks and higher susceptibility, pro-fraud attitudes should circulate more widely, potentially strengthening the superlinear scaling relationship. Conversely, sparser networks and lower susceptibility should dampen contagion and moderate the relationship between size and fraud outcomes.

## Methodology

This study used agent-based modelling (ABM) to examine how fraud may emerge as a dynamic property of organisational systems. ABM allows hypothesised generative mechanisms to be explicitly specified and observed in operation, providing a contrast to empirical approaches that must infer underlying processes from incomplete observational data. Modelling individuals as agents whose behaviour is governed by rules enables persons to be represented holistically while recognising irreducible capacities such as intention and agency [45]. ABM therefore provides a means of examining how interactions between multiple mechanisms generate observable outcomes over long sequences [46], in this case the patterns of fraud behaviour within organisations.

To construct the model, NetLogo 6.4.0 [47] was used. The model builds upon the agent-based model developed by Davis and Pesch [44], retaining the core mechanisms of social emulation and opportunity-based offending. It adds both dynamic network scaling and varying the size of the simulated organisation to address the research questions. The construction of the model is explained in more detail in this section.

Each agent (employee) within the ABM represents an individual in the simulated organisation who will commit fraud if and only if three binary conditions are satisfied simultaneously: the agent must have an opportunity to commit fraud, possess a motive, and hold a pro-fraud attitude (akin to rationalisation). This decision rule follows the structure of Davis and Pesch’s [44] model and was retained unchanged in the present study. The model operationalises occupational fraud as employee-level misconduct undertaken against the employing organisation for personal benefit. In practical terms, this corresponds most closely to forms of internal misappropriation such as expense fraud, procurement abuse, or other abuses of entrusted organisational resources. The model does not simulate corporate crime undertaken for organisational benefit, nor external frauds perpetrated by customers or third parties.

**Fig 1:**
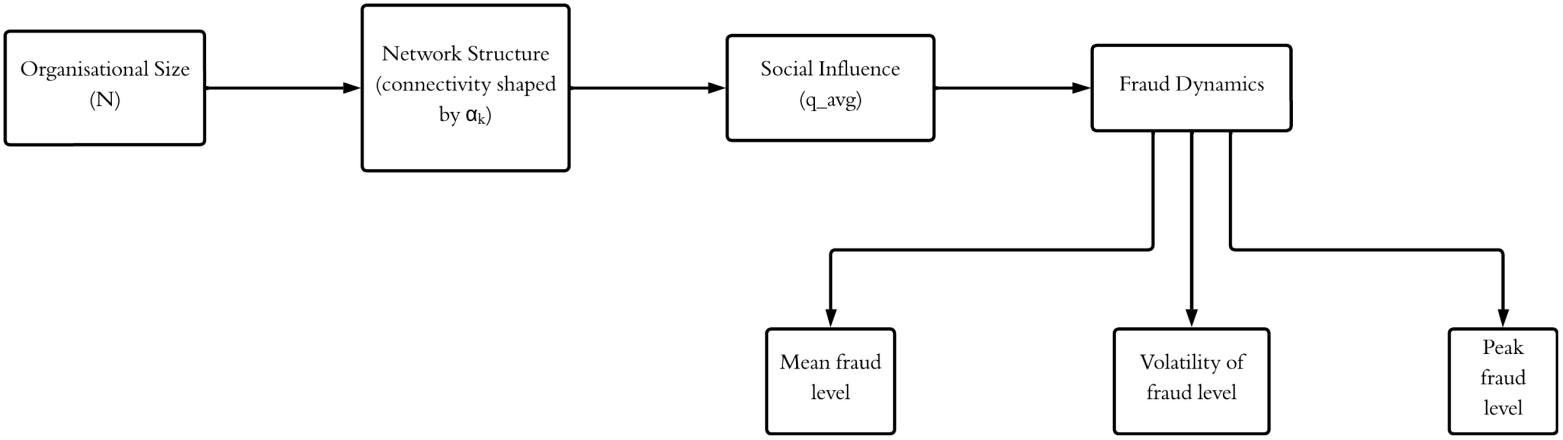
Conceptual framework linking organisational scale, network structure, social influence, and fraud outcomes.

The conceptual structure of the model is illustrated in Fig 1, which summarises how organisational size influences network structure, with connectivity shaped by a scaling parameter (αₖ). Together with susceptibility to social influence (q_avg), this determines how behaviours propagate within the system, giving rise to emergent fraud outcomes in terms of mean levels, peak events, and volatility.

At the start of each simulation run, every agent was independently assigned a binary value for attitude, motive and opportunity, each drawn from a Bernoulli distribution with probability 0.5. Because fraud requires the conjunction of all three states, the expected proportion of agents initially capable of committing fraud is therefore 0.5^3 = 0.125 (12.5%).

This parameterisation should not be interpreted as an empirical estimate of real-world fraud propensity. Rather, it represents a simplified generative mechanism designed to capture the structural logic of the fraud triangle within the model. The use of equal initial probabilities provides a neutral baseline from which the effects of interaction, scale, and network structure can be observed. It does not reflect any claim about the distribution of these attributes in real organisations.

The objective is not to calibrate individual behaviour but to explore how interactions between motive, opportunity and social influence can generate emergent patterns of fraud within organisational systems. Although the baseline arises mechanically from the model specification, it produces initial conditions broadly consistent with survey evidence suggesting that a non-trivial minority of individuals engage in economic misconduct.

For example, Shepherd et al. [48] report that 26% of respondents acknowledged committing at least one economic crime in the preceding year, including behaviours relevant to occupational settings such as accepting bribes, exaggerating expenses or falsifying timesheets. This echoed an earlier study by Karstedt and Farrall [49] where 61% of survey respondents in England and Wales reported having committed at least one of a list of ten types of dishonesty offence.

As with most research on fraud behaviour, such estimates are uncertain because they rely on self-reported conduct and may therefore underestimate the true prevalence of misconduct. Accordingly, the initial probabilities and binary representations should be understood as simplifying assumptions that allow the interaction of core mechanisms to be examined in isolation, rather than as empirically calibrated parameters intended to reflect real organisational populations.

Table 1, below, summarises the key variables, parameters, and agent attributes used in the model together with their operational definitions. Table 2 shows the structural parameters tested while Table 3 lists the behavioural parameters tested. Table 4 lists the measurements taken from the model and how these were analysed.

**Table 1:** Operational definitions of model variables and parameters.

Category	Variable/parameter	Description	Use in model
Agent attribute	Attitude	Whether an agent holds a pro-fraud disposition (analogous to rationalisation)	Binary state (0 = absent, 1 = present)
Agent attribute	Motive	Whether an agent experiences pressure or incentive to commit fraud	Binary state (0 = absent, 1 = present)
Agent attribute	Opportunity	Whether the organisational system affords the agent the opportunity to commit fraud	Binary state (0 = absent, 1 = present)
Agent state	Fraud status	Whether an agent commits fraud in a given tick	Fraud occurs only when attitude = 1 AND motive = 1 AND opportunity = 1
Network parameter	target_degree	Baseline average number of social ties per agent	Used to generate random social networks linking agents
Network parameter	αₖ (alpha_k)	Connectivity scaling parameter determining how network density changes with organisation size	Five values tested: 0, 0.25, 0.5, 0.75, 1
Network parameter	ref_size	Reference organisation size for network scaling	Connectivity equals base_degree when N = ref_size
Network parameter	base_degree	Baseline connectivity level at the reference organisation size	Used as the starting degree in the scaling equation
Network constraint	degree cap	Maximum number of network ties permitted	Network degree capped at 40 to avoid unrealistic densities
Behavioural parameter	q	Individual agent susceptibility to social influence	Probability that an agent adopts the observed neighbour’s attitude
Behavioural parameter	q_avg	Mean susceptibility to influence in the population	Ten values tested between 0 and 0.9
Structural variable	N	Organisation size (number of employee agents)	Nine values tested: 25–6,400
Simulation parameter	ticks	Length of each simulation	10,000 time periods
Simulation parameter	repetitions	Number of runs per regime	Twenty repetitions
Outcome variable	Peak fraud	Highest number of fraudulent agents observed in a simulation run	Recorded per simulation
Outcome variable	Average fraud	Mean number of fraudulent agents across all ticks	Recorded per simulation
Outcome variable	Fraud volatility	Standard deviation of fraud counts across ticks	Captures instability in fraud prevalence

**Table 2:** Structural parameters.

Parameter	Tested Values	Justification	Theoretical Implication
Organisational size (N)	25, 50, 100, 200, 400, 800, 1,600, 3,200, 6,400	Doubling sequence enables power-law analysis across a 256-fold range, approximating variation from small teams to large organisational units	Primary driver of fraud scaling dynamics; enables detection of superlinear, linear, or sublinear scaling relationships between N and fraud outcomes
Network connectivity scaling (αₖ)	0, 0.25, 0.5, 0.75, 1.0	Spans fully fixed network density (αₖ = 0) to fully proportional density growth (αₖ = 1); tests how interaction density changes with organisational size	Higher αₖ produces denser networks in larger organisations, potentially amplifying social contagion and fraud volatility
Reference size	100 (fixed)	Normalisation point for the network scaling function; held constant to isolate the effects of N and αk	Sets the baseline connectivity level from which network density scales with N; no independent effect on outcomes
Base degree	8 (fixed)	Approximates a plausible average number of informal workplace relationships per employee, consistent with prior ABM work	Sets baseline average co-worker interactions per agent; higher values would increase baseline contagion potential across all regimes
Degree cap	40 (fixed)	Prevents unrealistically high connectivity in large organisations where unconstrained scaling would produce implausible network densities	Constrains maximum individual connectivity; prevents network artefacts at large N

**Table 3:** Behavioural parameters.

Parameter	Tested Values	Justification	Theoretical Implication
Mean susceptibility to social influence (q_avg)	0.0, 0.1, 0.2, 0.3, 0.4, 0.5, 0.6, 0.7, 0.8, 0.9	Wide range from no social influence to near-total susceptibility; follows Davis and Pesch [44] in treating q as a theoretical scenario parameter rather than an empirical estimate	Higher q_avg produces stronger behavioural contagion; expected to amplify fraud volatility and moderate the relationship between organisational size and fraud dynamics; q_avg = 0 is the boundary condition where attitudes never change
Initial probability of attitude, motive, and opportunity	0.5 each (fixed)	Neutral baseline giving each agent an equal initial probability of possessing each fraud triangle condition; avoids imposing a priori assumptions about organisational culture	Produces an expected initial fraud rate of 12.5% (0.5³); provides a symmetric starting condition; the 10,000-tick run length ensures initial conditions are burned in before outcomes are recorded
Simulation length	10,000 ticks (fixed)	Long run ensures dynamics stabilise across all parameter regimes before outcomes are measured	Captures long-run attractor behaviour rather than transient dynamics; ensures scaling estimates reflect stable system properties
Repetitions per regime	20 (fixed)	Sufficient to characterise within-regime stochastic variability; balances precision with computational feasibility across 450 parameter regimes	Enables stable estimation of within-regime mean and variance; supports aggregation to regime means as the unit of analysis in regressions

**Table 4:** Output measures.

Measure	Definition	Analytical Treatment	Theoretical Relevance
Mean fraud	Mean number of fraudulent agents per tick across 10,000 ticks, averaged across 20 runs per regime	Log-transformed and regressed on ln(N); all 450 regime means retained	Captures average intensity of fraud dynamics; reflects the long-run central tendency of fraud activity within the system
Peak fraud	Maximum number of fraudulent agents in a single tick, averaged across 20 runs per regime	Log-transformed and regressed on ln(N); all 450 regime means retained	Captures worst-case fraud exposure; reflects the upper bound of fraud activity
Fraud volatility	Standard deviation of fraud counts across 10,000 ticks, averaged across 20 runs per regime	Log-transformed and regressed on ln(N); 405 regime means retained (45 regimes at q_avg = 0 excluded as zero social influence produces zero within-run variability)	Captures the instability and unpredictability of fraud dynamics; the key outcome exhibiting superlinear scaling with organisational size

*Note: The 450 parameter regimes comprise all combinations of 9 N values × 10 q_avg values × 5 αk values. Each regime was run 20 times, yielding 9,000 simulations in total.*

The generation of dynamic networks of heterogeneous agents is central to the model. Through these networks agents can exert influence upon each other. Each agent maintains a set of ties to others within their network. These ties represent informal workplace relationships and interactions. The network is the substrate through which behavioural norms spread. Networks were generated randomly to achieve an average number of social ties per agent, referred to as the ‘target degree’.

The model was allowed to run for 10,000 time periods (‘ticks’) per simulation, to allow for dynamics to be observed in the long run. The duration of ticks was not specified, as this is not important to the behaviour being modelled. Ticks should be understood as instances of interactions between agents rather than as fixed time periods. Twenty independent repetitions were run per regime, meaning a total of 9,000 simulations. These were run without fixed random seeds; stochastic variability is instead characterised through twenty independent repetitions per parameter regime.

During each tick, each agent observes the pro-fraud attitude of one of the other agents within its own network (which will either be pro-fraud (1) or not (0)). This simulates the interactions between co-workers. Parameter q_avg is the likelihood that an agent will adopt the same attitude as the neighbour that it observes. This was generated from a probability distribution from which each agent’s emulation likelihood q was drawn. Low values of q mean that emulation of the neighbour’s attitude is unlikely, whereas high values of q mean that it is far more likely.

This probability did not change as ticks progressed; there was no ‘drift’ nor random flips to simulate exogenous influences. Thus, if a fraudulent agent is paired with a non-fraudulent one, then the chance of their attitude changing to non-fraudulent is determined by probability q. Conversely, the probability of the non-fraudulent agent changing their own attitude to pro-fraud is determined by their own q value. However, unless their opportunity and motive were also both 1 then they would not become fraudulent themselves but would then have the potential to pass that attitude to others. The use of multiple parameter regimes across wide ranges allows the robustness of observed patterns to be examined under varying structural and behavioural conditions.

The model records three outputs: ‘peak fraud’, this being the highest number of fraudulent agents within a run; ‘average fraud per tick’, this value being the mean prevalence of fraud across the 10,000 ticks; and ‘fraud volatility’, this being the standard deviation of fraud counts across the 10,000 ticks.

Three independent variables were used. In line with the baseline model used by Davis and Pesch, the degree of social influence (*q_avg)* was varied. These values were not intended as empirical estimates from real organisations. They are theoretically contrasting scenario parameters designed to test how weak versus very strong social susceptibility alters the scaling behaviour of fraud. The lower values of q_avg represent a setting in which agents are relatively resistant to emulation, while higher values function as an strong contagion condition that makes amplification dynamics more visible. Their use therefore follows a scenario-based modelling logic rather than parameter calibration from observational data.

To examine how organisational scale influences fraud dynamics, nine population sizes were tested, whereas Davis and Pesch did not vary this in their study. These sizes follow a doubling sequence (25–6,400 agents), enabling analysis across multiple orders of magnitude while keeping the number of experimental conditions manageable. The range is intended to approximate variation from small teams or departments through to larger organisational units. Although not designed to replicate real-world firm size distributions, it provides sufficient variation to examine how system dynamics change with scale.

The existing empirical fraud datasets [4,10] do not provide this level of resolution, instead using only a small number of broad organisational size categories. While such banding is appropriate for descriptive reporting, it does not provide the granularity required to identify scaling relationships between organisational size and fraud prevalence. The logarithmic size progression used in this model therefore allows systematic exploration of whether fraud dynamics change as organisational systems increase in scale.

Crucially, variations in agent social network density were also modelled in some experimental regimes to test its effects. Where αₖ = 0 the network density does not grow with organisation size, with all agents being connected to a fixed number of neighbours, determined by target_degree in the model, this being the average number of agents in each network. The model then creates the number of edges required for the population to have this average degree, distributing those edges randomly across agents. As a result, individual agents may have more or fewer than eight neighbours that they can observe, but the organisation reliably exhibits a mean degree equal to target_degree.

The use of ten values of q_avg (0 to 0.9), five values of αₖ (0–1), and nine population sizes spanning a 256-fold range (25–6,400 agents) yields 450 distinct parameter regimes, each run twenty times, providing a systematic exploration of how the main findings vary across the full parameter space and functioning in effect as a comprehensive sensitivity analysis.

In other regimes, connectivity was increased moderately in line with organisation size, allowing agents to be connected to more co-workers in larger organisations, and more strongly to simulate higher density in more integrated organisations, up to a maximum of 40 connections to avoid unrealistic numbers of connections. The parameter ref_size was used as a normalisation point for the network scaling function. It specifies the organisation size at which the network has exactly the baseline connectivity (base_degree), and connectivity for larger or smaller organisations scales relative to this reference according to αₖ. Thus, the degree of connectivity scales with organisation size in this way:


degree(N)=base_degree×(N /ref_size)^αk, capped at 40

To quantify how fraud dynamics varied with organisational size under each regime, the output from the agent-based model was analysed using log–log linear regressions using IBM SPSS Statistics 30. Because agent-based modelling is being used, the simulated organisation represents the entire population of agents within the modelled system rather than a statistical sample drawn from a larger population. Each simulation therefore represents a complete artificial population of size N. Repeated simulation runs with identical parameter configurations are used to explore the stochastic variability inherent in the model rather than to estimate sampling error in the conventional statistical sense.

For every regime, fraud outcomes—peak fraud, average fraud across ticks, and volatility (standard deviation of fraud counts)—were transformed using natural logarithms and regressed on the logarithm of organisational size (ln N). To ensure independence of observations, the three outcome variables were first averaged across the twenty repetitions within each parameter regime prior to log transformation, yielding 450 regime means as the unit of analysis.

Model diagnostics were examined to assess the suitability of the log–log specification. Visual inspection of residual plots did not indicate systematic departures from linearity in log–log space, supporting the use of a linear approximation on the logarithmic scale. The unstandardised regression coefficient β provides an estimate of the scaling exponent, indicating whether fraud increases sublinearly (β < 1), linearly (β ≈ 1), or superlinearly (β > 1) with organisational size.

For fraud volatility, the 45 regimes at q_avg = 0 were excluded, as zero social influence produces zero within-run variability and consequently undefined log-transformed values; the volatility regression therefore uses 405 observations. As a sensitivity check, a nominal constant of 0.001 was added to all volatility values prior to log transformation, allowing all 450 regime means to be included. The resulting scaling exponent (β = 1.248, p < .001) remains clearly superlinear, confirming that the main result is not an artefact of the exclusion. The difference between the two estimates (1.393 versus 1.248) reflects the theoretical distinction between systems in which social influence operates and the boundary condition of zero social influence, where volatility is zero by construction.

The NetLogo model code and simulation output data used in this study are archived in a public Zenodo repository [50]. ChatGPT 5 (OpenAI) was used during the development of this study to assist with the generation, debugging, and refinement of the NetLogo agent-based model code. The tool was also used during manuscript preparation to identify typographical errors, improve readability, and enhance clarity of expression. All model design decisions, analytical procedures, interpretation of results, and final manuscript content were reviewed and approved by the author, who accepts full responsibility for the accuracy and integrity of the work.

### Model verification and validation

The model was developed to examine the behaviour of specified generative mechanisms rather than to reproduce or predict empirical datasets. As such, formal validation against real-world data was not undertaken.

Model behaviour was assessed through systematic exploration of parameter regimes varying organisational size, network connectivity scaling (αₖ), and susceptibility to social influence (q_avg). This approach allowed the consistency of observed patterns to be examined across a wide range of structural and behavioural conditions, rather than relying on a single parameter configuration.

Given the well-established limitations of empirical fraud data, which capture only a subset of underlying activity, the evaluation of the model is framed in terms of plausibility rather than empirical fit. The simulated outputs are therefore interpreted in relation to broad empirical regularities discussed in the literature, such as the persistence of non-zero fraud levels across organisational contexts and the heterogeneous distribution of fraud outcomes.

These comparisons are not intended as validation in a predictive sense, but as an indication that the model generates behaviour within a plausible domain consistent with existing empirical observations.

### Findings

This section examines how fraud outcomes vary with organisational size in the simulation model. Using regime means aggregated across the twenty repetitions per parameter configuration (N = 450 for mean fraud and peak fraud; N = 405 for volatility), log–log regressions were estimated to test whether fraud outcomes exhibit power-law scaling relationships with organisational scale. Three outcome measures were analysed: mean fraud levels, peak fraud events, and the volatility of fraud activity over time.

**Fig 2:**
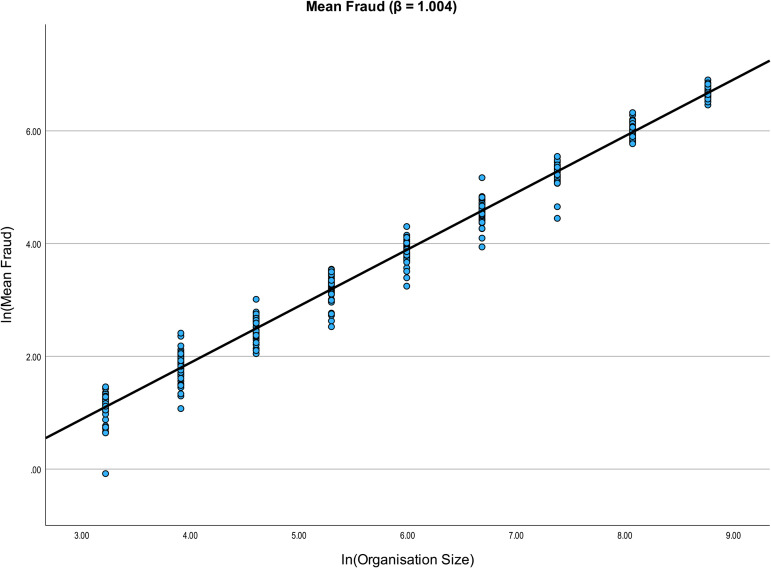
Log-log scatter plot of mean fraud against organisational size (N = 450 regime means). Each point represents the mean of average fraud per tick across 20 simulation runs for a given parameter regime. The regression line indicates approximately linear scaling (β = 1.004, R^2^ = 0.988), confirming that mean fraud levels increase approximately proportionally with organisational size across the full parameter space.

**Fig 3:**
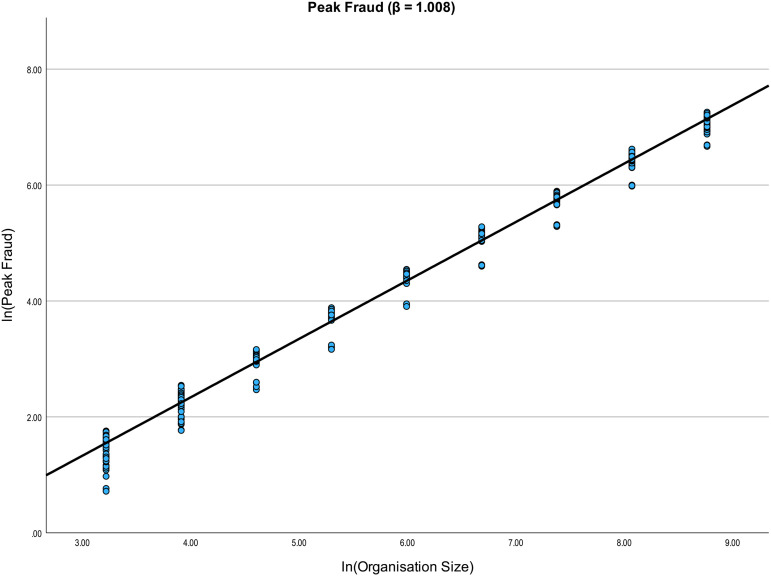
Log-log scatter plot of peak fraud against organisational size (N = 450 regime means). Each point represents the mean of peak fraud across 20 simulation runs for a given parameter regime. The regression line indicates approximately linear scaling (β = 1.008, R^2^ = 0.989), confirming that the highest level of fraud events increases approximately proportionally with organisational size.

**Fig 4:**
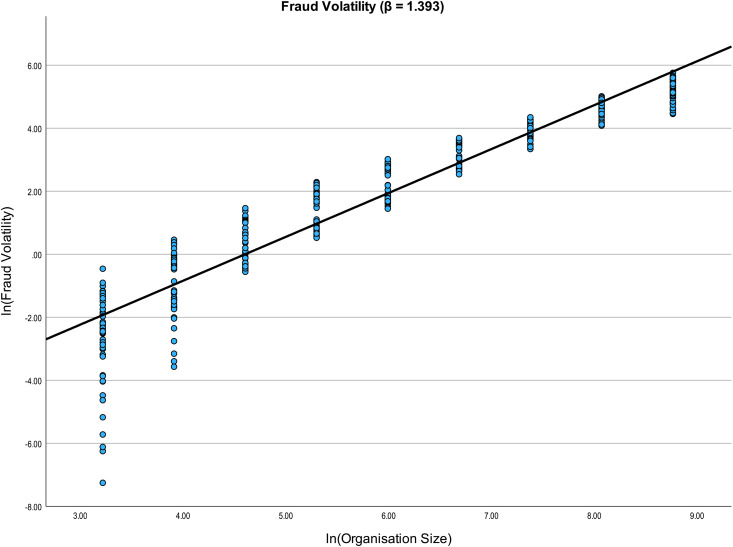
Log-log scatter plot of fraud volatility against organisational size (N = 405 regime means). Each point represents the mean standard deviation of fraud counts across 20 simulation runs for a given parameter regime. The 45 regimes at q_avg = 0 are excluded as zero social influence produces zero within-run variability. The regression line indicates superlinear scaling (β = 1.393, R^2^ = 0.898), indicating that the instability of fraud dynamics increases disproportionately with organisational size. The greater vertical spread of points relative to Figs 2 and 3 reflects the substantial moderating influence of social influence and network connectivity parameters on fraud volatility.

Fig 2, Fig 3, Fig 4 illustrate the log-log relationship between organisational size and these outcomes, while Table 5 reports the estimated scaling exponents from the log–log regressions. Table 6 then presents regression models that include the behavioural parameters and their interactions with organisational size. In addition to the direct effects of these parameters, interaction terms between organisational size and each behavioural parameter were also estimated to test whether the strength of the scaling relationship varies across behavioural regimes.

**Table 5:** Estimated scaling exponents (β) for fraud outcomes.

Outcome variable	Scaling exponent (β)	SE	t	p-value	R²	95% CI
Mean fraud	1.004	0.005	191.818	<.001	0.988	[0.994, 1.014]
Peak fraud	1.008	0.005	200.548	<.001	0.989	[0.998, 1.018]
Fraud volatility	1.393	0.023	59.461	<.001	0.898	[1.348, 1.438]

*Note: Estimated from log–log regressions of fraud outcomes on organisational size. N = 450 regime means for mean fraud and peak fraud; N = 405 regime means for volatility, as 45 regimes at q_avg = 0 produced zero volatility and were excluded from the log transformation.*

**Table 6:** Interaction regression results for fraud outcomes.

Variable	Mean fraud (ln_fraud)	95% CI	Peak fraud (ln_peak)	95% CI	Fraud volatility (ln_sd)	95% CI
ln_agents	1.017***	[0.993, 1.041]	0.949***	[0.929, 0.969]	0.835***	[0.745, 0.925]
q_avg	0.020	[-0.204, 0.244]	−0.056	[-0.238, 0.126]	−5.337***	[-6.174, -4.500]
αₖ	0.097	[-0.085, 0.279]	−0.487***	[-0.634, -0.340]	−3.121***	[-3.732, -2.510]
ln_agents × q_avg	−0.008	[-0.043, 0.027]	0.057***	[0.028, 0.086]	0.686***	[0.553, 0.819]
ln_agents × αₖ	−0.018	[-0.047, 0.011]	0.066***	[0.042, 0.090]	0.431***	[0.333, 0.529]
Constant	−2.187***	[-2.338, -2.036]	−1.426***	[-1.548, -1.304]	−2.185***	[-2.746, -1.624]
R²	0.988		0.992		0.943	
N	450		450		405†	

Note: * p < .05 ** p < .01 *** p < .001,

Note: *† as 45 regimes at q_avg = 0 were excluded from the volatility regression as zero social influence produces zero within-run variability (undefined ln(0)); non-significant coefficients shown without asterisks.*

Across the simulated organisational sizes, clear log–log relationships emerge between organisation size and fraud outcomes. The slopes of these relationships correspond to the estimated scaling exponents reported in Table 5. Mean fraud levels scale approximately linearly with organisational size (β = 1.004, p < .001), indicating that average fraud activity increases approximately in proportion to organisation size. This result does not support Expectation 1, which predicted superlinear scaling of mean fraud levels. Peak fraud events also scale approximately linearly with organisational size (β = 1.008, p < .001), indicating that the magnitude of extreme fraud events increases approximately in proportion to organisational size. Fraud volatility exhibits a superlinear relationship with organisation size (β = 1.393, p < .001), indicating that larger organisational systems exhibit disproportionately greater fluctuations in fraud activity.

These results indicate that organisational scale shapes both the level and the instability of fraud dynamics, but in distinct ways. Average fraud levels and peak events both scale approximately in proportion to organisational size. A doubling of organisational size is associated with an increase in average fraud levels of approximately 2^1.004 ≈ 2 times and peak fraud levels of approximately 2^1.008 ≈ 2 times. This confirms that both increase approximately proportionally with size.

Volatility, on the other hand, increases substantially more rapidly (β = 1.393): doubling organisational size is associated with an increase of approximately 2^1.393 ≈ 2.62 times, indicating disproportionately greater instability in fraud dynamics as organisations grow.

### Illustrative example

To illustrate these scaling patterns concretely, predicted fraud outcomes were derived from the regression models for two contrasting organisational sizes – N = 100 and N = 3,200, a 32-fold difference. At N = 100, the model predicts an average of approximately 12 fraudulent agents per tick and a peak of approximately 19 agents. At N = 3,200, these rise to approximately 392 agents on average and approximately 623 at peak. In both cases the increase is close to the 32-fold proportional expectation, confirming that mean fraud and peak fraud scale approximately linearly with organisational size. The per-capita fraud rate remains approximately stable across this 32-fold size difference, consistent with β ≈ 1.0 for both outcomes.

The picture is strikingly different for volatility. The predicted standard deviation of fraud counts rises from approximately 1.1 agents at N = 100 to approximately 124 agents at N = 3,200. Under proportional scaling the expected figure would be around 35 agents; the model predicts more than three times that amount. This disproportionate amplification of instability, while average and peak levels track size closely, illustrates the central finding: larger organisations do not experience more fraud per capita, but they experience it far more erratically.

### Influence of behavioural parameters

To examine the role of behavioural and structural parameters within the simulation, additional regression models were estimated including susceptibility to social influence (q_avg) and network connectivity scaling (αₖ). The results are reported in Table 6.

The regression results reveal a clear separation between the determinants of average fraud levels and the determinants of fraud volatility. For mean fraud, none of the behavioural parameters, nor their interactions with organisational size, reach statistical significance. Mean fraud levels are therefore almost entirely predicted by organisational size alone (R² = 0.988). This suggests that organisational scale is the dominant determinant of average fraud levels within the simulated system. For peak fraud levels, organisational size remains the dominant predictor (β = 0.949), but network connectivity scaling (αₖ) and both interaction terms are also statistically significant. This indicates that the magnitude of extreme fraud events is modestly but meaningfully shaped by network structure and its interaction with organisational size, in addition to scale itself.

Meanwhile, the behavioural dynamics of social influence and network connectivity shape how erratically and unpredictably fraud manifests within the system, without significantly altering the average level at which it occurs. This suggests a structural separation: mean fraud levels are determined almost entirely by organisational scale, consistent with a fixed per-agent fraud probability scaling with system size. Volatility reflects the dynamics of social contagion and network propagation, scaling superlinearly and responding significantly to susceptibility to influence and network connectivity. The structure and culture of organisational systems therefore matter primarily for managing how erratic fraud becomes, rather than how much of it occurs on average.

## Discussion

### Revisiting the research questions

The three research questions posed by this study can now be answered directly by reference to the simulation results. Firstly, mean fraud scales approximately linearly with organisational size (β = 1.004). The per-capita fraud rate is approximately constant across organisational sizes when averaged across parameter regimes. Fraud volatility, however, scales superlinearly (β = 1.393), indicating that larger organisations experience disproportionately more erratic fraud dynamics. Peak fraud events also approach proportionality (β = 1.008). A 32-fold increase in organisational size is therefore associated with an approximately proportional increase in mean fraud, but with a substantially disproportionate amplification of volatility.

The approximately linear scaling of mean fraud is notable in that it does not support Expectation 1. This suggests that social contagion mechanisms alone, as modelled here, are insufficient to produce superlinear mean fraud scaling. The superlinearity observed in urban crime contexts [e.g., 14,37] may therefore depend on factors absent from the present model, such as a degradation of control capacity with organisational size, or disproportionate expansion of opportunity structures.

Secondly, network connectivity and susceptibility to social influence shape fraud volatility but not mean fraud levels. The results in Table 6 show that for mean fraud, neither q_avg, αₖ nor their interactions with organisational size reach statistical significance. For volatility, all five predictors are highly significant, with large interaction effects indicating that social influence and network connectivity substantially amplify the erratic character of fraud dynamics in larger organisations.

Thirdly, organisational scale amplifies volatility far more strongly than it amplifies average fraud levels. This is the most novel finding of the study. While mean fraud doubles approximately with each doubling of organisational size, volatility scales at β = 1.393, meaning fluctuations in fraud activity grow substantially more rapidly than organisation size. Larger organisations therefore do not face more fraud per capita on average, but dramatically more unpredictable fraud dynamics. This finding has no known direct counterpart in prior empirical fraud research.

### Fraud as a system-level phenomenon

The results demonstrate that fraud is a dynamic, emergent property of organisational systems. The same behavioural mechanisms—individual susceptibility to social influence and network-mediated diffusion of pro-fraud attitudes—generate qualitatively different fraud dynamics depending on the structural scale of the system in which they operate. Larger systems do not simply contain more fraud; they exhibit fraud that is less stable and therefore harder to predict.

The results partially qualify the assumption of per capita linearity. For mean fraud levels, the per-capita rate is approximately constant across organisational sizes, meaning that larger organisations do not experience disproportionately more fraud, on average. However, for volatility, proportionality fails substantially: the instability of fraud dynamics grows far more rapidly than organisational size, making fraud behaviour substantially more erratic and unpredictable in larger systems.

These patterns are also qualitatively consistent with the empirical inconsistency in survey findings on organisational size and fraud [4,10]. Because volatility scales disproportionately with size, cross-sectional surveys capture larger organisations at varying points in their fraud cycle, producing highly variable results that are difficult to replicate across studies. The volatility finding therefore provides a plausible systemic mechanism for the empirical ambiguity that detection-based datasets alone cannot explain.

Fraud exhibits two distinct scaling regimes: a superlinear one for volatility and approximately linear scaling for both peak and average levels. This has implications for how fraud risk is measured. Focusing on aggregate incident counts captures only one dimension of this far richer dynamic. Periods of low detected fraud may reflect temporary stability rather than genuine control effectiveness, while episodic peaks can overwhelm safeguards in ways that average measures fail to anticipate. Attending to the full distribution of fraud behaviour, understanding its central tendency, its extremes, and its variability over time will provide a more accurate picture of systemic risk.

### Implications for fraud theory

This paper introduces organisational scale as an explicit structural variable in occupational fraud research, demonstrating that varying organisational size produces a systematic divergence in the fraud dynamics observed. This extends the theoretical scope of computational approaches to fraud beyond Davis and Pesch’s [44] fixed-population design. Furthermore, the results establish fraud volatility as the key dimension of disproportionate risk in larger organisations. While both mean fraud and peak levels are approximately stable across organisational sizes, the instability of fraud dynamics grows substantially more rapidly. This distinguishes the risk profile of larger organisations not through higher average fraud, but instead through significantly greater unpredictability.

These contributions complement, rather than replace, individual-level frameworks such as the fraud triangle, which remains a valid account of why individual agents engage in fraud. The model’s decision rule is derived directly from Cressey’s framework and retained unchanged from Davis and Pesch’s design. What the present study adds is an account of why identical individual-level mechanisms produce very different aggregate outcomes depending on the structural scale of the system in which they operate. The fraud triangle explains the conditions under which fraud occurs at the individual level; organisational scale and network structure explain how much, how often, and how erratically it emerges at the system level.

### Positioning within Critical Realism and Complexity Theory

As set out in the literature review, this study is grounded in critical realist and complexity-theoretic perspectives that conceptualise fraud as an emergent property of complex adaptive systems. The ABM operationalises these frameworks computationally. It represents the Real domain in Bhaskar’s [51,52] stratified ontology: a space in which generative mechanisms can be specified, activated and observed without the epistemic distortions introduced by detection bias and underreporting. Within the simulation, every fraudulent event is observable, enabling systematic exploration of mechanism–outcome relationships that detected-incident datasets cannot support.

The results reinforce Bunge’s [31] systemic view that individual actions cannot be understood without reference to the systems in which they are embedded. Fraud does not arise from individual dispositions alone: it arises from the interaction between those dispositions and the structural conditions that shape how motives, opportunities and social influences combine. The present study extends this perspective by demonstrating that organisational scale is one such structural condition, and that its effect on fraud dynamics is systematic and non-linear.

These results should nonetheless be interpreted as exploratory. The model is a deliberate simplification, with mechanisms and parameters that are theoretically grounded but not empirically calibrated. The patterns generated represent plausible system-level dynamics rather than predictions of behaviour in specific real organisations. They provide a basis for theoretically informed hypotheses that future empirical research can test.

### Implications for policy and practice

Established approaches to fraud prevention have largely remained aligned with the fraud triangle perspective, even though Cressey himself did not intend his research to serve as a direct framework for prevention [21,53]. The simulation results indicate that organisational scale exerts a dominant structural influence on fraud outcomes, while interaction networks and susceptibility to social influence shape the variability of fraud activity. Larger organisations do not face disproportionately greater fraud exposure on average: mean fraud scales approximately proportionally with size, but the instability of fraud dynamics scales substantially more rapidly. Effective fraud prevention therefore requires attention both to the individual conditions that motivate misconduct and to the systemic features that drive volatility within organisations.

Controls aimed solely at influencing individual behaviour may reduce detected instances of fraud but do not necessarily address the deeper structural conditions that generate fraud risk [9]. In critical realist terms, organisational knowledge of fraud is confined to the Empirical domain, i.e., those incidents that are observed. Expanding detection capabilities, for example through data analytics, may reveal more fraud that has already occurred, shifting cases from the Actual to the Empirical domain. While this may enhance deterrence and accountability, it does not necessarily address the latent generative mechanisms that create new forms of fraud risk.

In practice, improving organisational culture is often seen as a central component of fraud prevention, typically through awareness and ethics programmes, leadership messaging, and improved communication across teams. However, the same interaction networks that allow ethical norms to spread can also transmit misconduct. Research on behavioural contagion shows that antisocial behaviour can propagate through workplace networks, sometimes more readily than prosocial behaviour [39–41]. The model results suggest that where employees are highly susceptible to the attitudes of those around them, fraud dynamics can become more erratic and volatile within organisational networks. This highlights a dilemma: strengthening communication and connectivity within organisations may promote ethical culture, but it can also accelerate the diffusion of harmful norms if misconduct becomes normalised within influential groups.

For practitioners, this implies that attention should be paid not only to formal control systems but also to the structure and operation of organisational influence networks. Interventions that focus solely on detection or individual deterrence may overlook the role of interaction networks and behavioural propagation. Techniques such as organisational network analysis can help identify highly connected individuals who shape norms across teams. Ensuring that these key actors reinforce organisational values may therefore be particularly important in preventing the normalisation of fraudulent behaviour.

A further implication concerns the resourcing of counter-fraud capability. Because average fraud levels scale approximately proportionally with organisational size, proportional increases in counter-fraud resources should, in principle, maintain equivalent control effectiveness over average fraud levels as organisations grow. The more challenging problem concerns volatility. The model shows that fraud volatility scales disproportionately with organisational size, but in real organisations, this volatility is largely invisible.

Organisational knowledge of fraud dynamics is confined to what is detected and reported: a series of snapshots rather than a continuous picture. Practitioners cannot observe the underlying distribution of fraud activity, only its occasional surface manifestations. This creates a structural resourcing problem that better measurement cannot fully solve. At times, fraud activity within a system may be at its lowest ebb; at others, the same system may be experiencing peak conditions that overwhelm existing controls. Neither state is predictable from the detected data available.

It is important to recognise that the invisibility of these dynamics does not diminish their reality or their consequences. In critical realist terms, the generative mechanisms that drive fraud volatility operate within the Real domain regardless of whether their effects reach the Empirical domain through detection. Unobserved fraud is not therefore an absence of fraud: it is fraud whose causal effects are being produced but not yet encountered. Controls that fail to account for this may appear effective while the underlying conditions for escalation remain intact and active.

The implication is not that organisations should invest in ever more sophisticated measurement in the hope of achieving complete visibility. Such visibility is not achievable in principle, given the inherently hidden character of fraud. Rather, counter-fraud systems should be designed with an explicit acceptance that a significant proportion of fraud dynamics will always remain unobserved, and that resilience to unpredictable variation, rather than optimisation for average conditions, should be a design objective for control frameworks in larger organisations.

For policymakers and regulators, the findings suggest that organisational scale and complexity should be explicitly considered when designing governance requirements. As organisations become larger and more interconnected, the number of potential interactions between employees increases, altering network structures and creating conditions in which fraud dynamics may become more volatile and unpredictable. Ensuring that oversight, audit, and investigative capacity are aligned with organisational complexity, and with the greater instability that complexity brings, may therefore be important in maintaining effective fraud control.

Beyond resourcing and governance, the scaling relationships identified in this study have implications for how organisations design and target counter-fraud interventions. Parameter regimes characterised by higher levels of connectivity and susceptibility to social influence are associated with higher levels of fraud volatility and greater dispersion in outcomes. In organisational settings, this suggests a need for more targeted, system-aware approaches that focus on identifying and managing high-risk interaction structures, such as densely connected networks or areas of strong behavioural influence.

These findings also highlight the limitations of evaluating fraud risk solely through detected incident counts. Periods of apparent stability may reflect temporary lulls rather than genuine control effectiveness, while episodic peaks may overwhelm existing safeguards. Attending to the full distribution of fraud behaviour—its average level, its peak, and its variability over time—therefore provides a more meaningful basis for organisational risk assessment than snapshot measures alone.

More fundamentally, these findings suggest that organisations would benefit from reorienting their approach to fraud risk identification away from individual-centred frameworks toward a more structural analysis of the conditions that make fraud possible. Building on the Fraud Field Theory framework developed in previous work [9,13], the present model supports an approach grounded in affordance mapping: the systematic identification of the structural and cultural conditions that create the situational affordances through which fraud becomes possible. This reframing locates fraud risk within the Real domain as a property of system design rather than as a consequence of individual intention and capability.

The fraud field can in this sense be understood as a structured space of potential: the configurations of controls, incentives, routines, and network structures that determine where and how fraud could plausibly occur. Meanwhile, detection activity functions as a mechanism for testing whether those potentials are being realised, rather than as a primary source of knowledge about risk. Crucially, this framing is durable across changing conditions: although the specific techniques and technologies through which fraud is committed evolve continuously, the underlying mechanisms by which organisational systems generate affordances for fraud are more stable. Risk identification grounded in affordances therefore targets the generative layer of fraud dynamics rather than its surface expressions. This is more consistent with the complexity-informed critical realist perspective developed throughout this paper.

In operational terms, this approach implies a different sequencing of fraud risk management activity from that typically found in practice. Where case-led approaches use historical detections as the primary means of identifying where risk resides, affordance mapping operates upstream: it identifies where within the system fraud could plausibly occur independently of whether it has yet been detected and directs investigative resources toward those areas accordingly. Detection then becomes a way of testing whether identified affordances are being exploited, building cumulative knowledge of the system rather than simply confirming what is already known. This is a meaningful shift, because case-led approaches are structurally self-reinforcing as they direct attention toward patterns that have already produced observable outcomes, leaving novel or emerging risks in shadow. Combining affordance-based risk identification with empirical case data allows organisations to work toward a more complete picture of their fraud exposure, attending to latent vulnerability alongside realised harm.

A related benefit of this approach concerns the confidence it can provide under conditions of irreducible uncertainty. Where affordances are identified through structural analysis and found to correspond to areas already covered by effective controls, this offers meaningful assurance that the control environment reflects the actual architecture of risk rather than simply the record of past incidents.

Where structural analysis reveals plausible pathways for fraud that are not currently addressed by controls, this constitutes a substantive signal of exposure that warrants attention irrespective of whether any cases have been recorded. The absence of detected fraud should not be taken as evidence of absence, as it may reflect the limits of detection capability rather than the absence of underlying activity. To interpret a clean detection record as confirmation of safety is to commit precisely the epistemic error that a critical realist framework cautions against: treating the boundaries of the observable as the boundaries of the real. Affordance mapping addresses this by anchoring risk assessment in what the system makes structurally possible, rather than in what it has so far revealed.

Ultimately, the results suggest that fraud control should be understood as the governance of complex organisational systems whose structural properties can amplify or suppress fraudulent behaviour as those systems grow, rather than as the attempted management of individual misconduct. These implications are necessarily tentative and should be interpreted as illustrative of how scaling dynamics may influence organisational fraud risk, rather than as prescriptive guidance applicable to all organisational contexts. The model provides insight into possible mechanisms through which fraud dynamics may arise, but further empirical work is needed to assess how these patterns manifest in real organisational contexts.

### Limitations and directions for future research

#### Organisational structure and network realism.

Organisational networks in the model are generated randomly to produce heterogeneous interaction structures. Real organisations typically exhibit hierarchical reporting relationships, departmental clustering, and power dynamics associated with positional authority, all of which shape how norms and behaviours propagate. These features may amplify or dampen the scaling relationships identified here, particularly in large organisations that are multi-divisional, geographically dispersed, or functionally differentiated. Future research should incorporate more realistic network structures, including small-world, modular, and hierarchically branching designs. This would enable the testing of the robustness of the scaling findings under conditions more representative of real organisations.

### Agent behaviour and parameterisation

Agent behaviour is governed by the simplified binary states for opportunity, motive and pro-fraud attitude, with equal initial probabilities assumed across all agents. This simplification captures the core logic of the fraud triangle but necessarily omits the psychological, social and organisational heterogeneity present in real workforces. Parameter values for susceptibility to social influence and network connectivity were selected to span a theoretically meaningful range rather than being calibrated to empirical data, meaning results should be interpreted as illustrating possible dynamics rather than estimating their real-world magnitude. Future research should explore continuous and heterogeneous representations of agent attributes and seek to calibrate key parameters using organisational data, such as internal audit findings, detected fraud incidents, or employee survey evidence on cultural susceptibility.

### Empirical calibration and external validation

The simulation environment presented in this paper is closed: regulatory changes, economic shocks, technological developments and governance reforms are excluded. Additionally, every fraud event within the simulation is observable, whereas real-world datasets capture only detected incidents. Together these features mean that formal empirical validation is not undertaken, and the findings should be understood as plausible generative dynamics rather than estimates of real fraud rates in specific organisations. Collaboration with organisations possessing detailed records of communication patterns, transaction flows, or detected fraud incidents would enable the model to be applied to specific contexts and its outputs compared with observed detection patterns. This would provide a basis for iterative model development and empirical grounding.

### Scope of modelled fraud

The model represents occupational fraud committed by internal agents against their employing organisation. It does not include fraud perpetrated by, or in collusion with, customers, suppliers or other external parties. The findings are therefore most applicable to organisations characterised by relatively dense informal interaction networks and less applicable to contexts where external fraud vectors or formal control environments dominate. Future research should extend the model to incorporate external actors and multi-party fraud scenarios to provide a more comprehensive account of the full range of organisational fraud risk.

### Priorities for future research

Three directions are identified as the most important for researchers building on this work. Firstly, incorporating empirically observed network structures drawn from organisational communication records, reporting hierarchies, or transaction logs would allow the robustness of the scaling relationships to be tested under structurally realistic conditions. This would directly address the most significant limitation of the current model. Secondly, calibrating model parameters against specific organisational data, using partial indicators such as audit findings or detected fraud rates, would allow simulated vulnerability profiles to be compared with observed risk patterns, bridging the gap between exploratory simulation and practical application. Thirdly, using the calibrated model as a testbed for policy interventions through systematically varying governance structures, network connectivity, or cultural susceptibility to influence would enable the relative effectiveness of different counter-fraud strategies to be examined in a controlled simulated environment. This would provide theoretically grounded guidance for practitioners before real-world implementation.

## Conclusion

This study examined whether fraud scales proportionally with organisational size and whether network connectivity and susceptibility to social influence moderate that relationship. Mean fraud scales approximately linearly with organisational size (β = 1.004), while fraud volatility scales superlinearly (β = 1.393) and peak events scale approximately proportionally (β = 1.008). A 32-fold increase in size is associated with an approximately proportional increase in mean fraud, but with a substantially disproportionate amplification of volatility. Network connectivity and susceptibility to social influence significantly moderate fraud volatility but have no significant effect on mean fraud levels.

The study makes several theoretical contributions to economic criminology and organisational complexity research. It demonstrates that mean fraud scales proportionally with organisational size while volatility scales superlinearly, showing that scale determines not how much fraud occurs on average, but how unpredictably it manifests. Furthermore, it establishes fraud volatility as a theoretically distinct and practically important dimension of fraud risk, disproportionate to organisational size in ways that average incidence measures cannot capture. Finally, it shows that per capita linearity holds for average fraud levels but substantially fails for volatility, with implications for how risk assessments based on incident rates should be interpreted in larger organisations. These contributions complement individual-level accounts such as the fraud triangle by showing how identical individual mechanisms produce qualitatively different system-level dynamics at different organisational scales.

For practitioners and policymakers, the results suggest that proportional fraud control resourcing is adequate for managing average fraud levels, which scale proportionally with size. However, managing volatility—the disproportionately erratic character of fraud dynamics in larger organisations—requires a different kind of response. Because fraud volatility is largely invisible in real organisations, the answer does not lie primarily in better measurement: the hidden character of fraud dynamics is irreducible, not merely a current limitation of detection capability. Rather, it implies designing counter-fraud systems for resilience to unpredictable variation, and reorienting risk identification toward the structural affordances that make fraud possible rather than the historical cases that have been recorded. Interventions targeting social influence and network connectivity can reduce instability without necessarily reducing the average fraud rate, and an affordance-based approach to risk identification provides a more complete basis for directing those interventions than case-led detection alone.

As an exploratory simulation study, these findings are indicative of plausible mechanisms rather than predictive of outcomes in specific organisations. The most important direction for future research is empirical: testing whether the scaling relationships identified here are present in real-world data, using longitudinal datasets with sufficient resolution to distinguish average levels from volatility. Extending the model to incorporate more realistic network structures would allow the robustness of these relationships to be examined under more representative organisational conditions. The open nature of social systems means that complete knowledge of fraud will always remain out of reach. Nevertheless, modelling approaches grounded in complexity theory and critical realism illuminate the otherwise hidden mechanisms through which fraud emerges within organisational systems, and their continued development offers a promising path for advancing both the scholarly understanding of fraud and the design of more effective, system-level responses.
