# Supplementary material for: Organisational scale and fraud volatility: An exploratory agent-based simulation of occupational fraud dynamics
Source: PLoS One. 2026 Jul 17;21(7):e0342964. doi: 10.1371/journal.pone.0342964 (PMC13378982; doi:10.1371/journal.pone.0342964)
Supplement: Attachment — Submitted filename: Reviewer Attachments for Manuscript Number PONE-D-25-65420.pdf [file pone.0342964.s001.pdf]

Critical Review of Research "Detecting Emergent Organizational Fraud Dynamics using Agent-Based Modeling"

Introduction (Background)

This study opens by emphasizing that organizational fraud remains poorly understood, with most previous studies focusing too heavily on individual perpetrators. The "fraud triangle" approach, comprising personal factors (pressure, opportunity, and rationalization), has been cited as dominating both academic explanations and professional practice, neglecting the influence of the broader organizational system. This introduction logically builds the argument that a multi-level perspective is needed that considers the interaction of individuals with the systems in which they exist, in line with Mario Bunge's systemic crime view.

Criticism: While it's true that the fraud triangle is popular, the authors tend to overstate the research gap at the organizational level. Previous studies have acknowledged the role of organizational culture and the internal control environment in influencing fraud, for example through the concept of "tone at the top" or the COSO framework. However, this is under-emphasized. The introduction fails to mention the literature on organizational factors (structure, internal controls, etc.) known to impact fraud. Consequently, the impression is created that there has been no attempt to understand fraud systematically, even though several management and audit studies have addressed it empirically.

In terms of logic and consistency, the problem presented is clear: the author wants to prove that fraud in organizations is a complex phenomenon that emerges from the system, not simply the sum of individual motivations. The problem formulation and research questions are consistent with this gap, for example, whether fraud rates increase proportionally with organizational size, and how structural connectivity and social influence interact to shape fraud dynamics. These questions are relevant and arise from the background presented.

However, an intelligent skeptic might question the implications of the initial premise: Is it true that organizational fraud is “poorly understood” simply because the previous approach was individualistic? While the individualistic approach may be dominant, it doesn’t mean we are completely blind to systemic factors. Furthermore, a skeptic might argue that viewing fraud as an “emergent property of the system” risks obscuring individual responsibility—as if the perpetrator were merely a product of the system. The initial approach, which heavily emphasizes critical realism and complexity theory, also seems philosophically heavy-handed. This can reduce readability for practitioners who prefer concrete explanations rather than theoretical terms like “epistemic fallacy” or stratified ontology.

Framework Alternatives: As an alternative or complementary framework, the author could refer to other theories such as Routine Activity Theory in an organizational context or the concept of fraud risk factors from the auditing literature. For example, organizational size may influence the level of oversight and the likelihood of fraud detection, a more empirical perspective on institutional control. Linking the issue to real-world data—for example, a survey of fraud rates in SMEs versus large corporations—would strengthen the urgency of the issue. Recommendations for improvement: The introduction should include brief empirical evidence (audit reports or case studies) demonstrating how large-scale fraud can occur due to system failures, to strengthen the

justification for a systemic approach. Also, acknowledging existing research in the field of organizational culture and internal control would provide a more balanced background.

Theoretical Review (Literature Review)

The author reviews the literature by highlighting the dominance of the fraud triangle paradigm and its variants (fraud diamond, fraud pentagon) and underlining the weak empirical basis of these models. The literature reviewed shows that most fraud research still focuses on why individuals commit fraud, rather than how organizational systems encourage or prevent fraud. The author cites other conceptual efforts such as the “fraud field” model by Button et al. (2023) comparing threats and safeguards, and the author's own previous research (Freeman, 2025) revising the fraud field with a complex adaptive system approach. In addition, previous agent-based modeling (ABM) studies are discussed: Kim & Xiao (2008) who simulated fraud in welfare programs, and especially Davis & Pesch (2013) who formed the basis of this research model. Davis & Pesch modeled 100 employees with the rule that agents who have motives, opportunities, and attitudes that support fraud will commit fraud. They found that imitation probability (q) above the threshold of 0.3 triggers large fluctuations in the number of fraudsters (U-shaped pattern), while q below 0.3 makes the number of frauds stable low (inverse-U pattern). The authors emphasize that the model is limited (only 100 agents, static network structure) due to computational constraints, and does not consider organizational scale or changes in network density. From this review, the author identified a gap in that no research has explored the effects of organizational size and internal network dynamics on fraud rates.

Criticism: Overall, the literature review lacks depth and comprehensiveness. First, the literature reviewed is relatively limited to the theoretical framework of the fraud triangle and a few ABM studies. The authors do not review empirical studies on organizational factors influencing fraud. For example, research in forensic accounting or corporate governance has shown a correlation between internal control weaknesses or a permissive culture and fraud incidents, but these are not reflected in the review. By ignoring such empirical findings, the authors risk creating a strawman argument, suggesting that there is only one individual approach versus their own complex approach.

Second, although the authors mention the concepts of "fraud field" and complexity, the literature on social network theory in the context of white-collar crime is barely discussed. Yet, there are studies on collusion networks or whistleblowing that are relevant to understanding social interactions in fraud. Similarly, analogies from the urban scaling literature (e.g., Bettencourt et al.'s on city scale and crime) appear fleetingly. However, it doesn't explain how the concept of allometric scaling applies to business organizations. This makes the theoretical foundation seem less solid—readers aren't given a complete picture of whether increasing organizational size has been empirically found to impact fraud rates or whether it hasn't been studied at all.

Implied Assumptions: This review assumes that the individualistic approach is incapable of explaining the variation in fraud at the organizational level, and that a complex-adaptive approach is a better solution. This assumption has not been validated; it is possible that simple factors such as the number of transactions or structural complexity in large organizations lead to more fraud detection, without necessarily attributing this to the emergent nature of complexity. The existing literature on fraud risk factors (e.g., number of employees, volume of funds managed, etc.) should be reviewed to see if scale-dependent patterns have been observed.

Consistency: The literature section successfully identifies a research gap that serves as the study's objective: that organizational scale and internal network density have not been studied for their impact on fraud dynamics. This is consistent with the introduction. However, the literature's

arguments are somewhat biased toward the author's own perspective (critical realism). For example, the literature concludes by stating that most fraud studies focus on observables (detected cases, individual motivations) and fail to consider latent mechanisms at the system level. This aligns with the philosophy of critical realism, but the authors fail to address potentially opposing perspectives, such as the purely positivist view that is skeptical of simulation models that are not verified by data. Ignoring these alternative perspectives makes the review seem one-sided.

Counter-argument (Skeptical Perspective):A skeptic could argue that the individual versus systemic explanation is a long-standing continuum in social science—not a new gap. Large organizations naturally have more complex bureaucracies, making fraud more difficult to detect; this is a simple, linear explanation without the need for the concept of emergentism. Thus, the claim that “scale and culture hold the potential to drive fraud levels in unexpected ways” is unfounded. This needs to be proven, not simply assumed from complexity theory. Skeptics might also point out that the criticized fraud triangle literature is empirically weak, but the author's ABM model also lacks empirical support—meaning it replaces one data-poor approach with another, also untested one.

Alternatives & Suggestions: To strengthen the theoretical review, authors should: (1) Broaden the scope of the literature – include empirical findings on the relationship between organizational size and fraud incidents (e.g., global fraud survey reports often report that larger companies suffer greater absolute fraud losses, but perhaps smaller percentages), and research on organizational culture and internal control as influences on fraud. (2) Link the complexity framework to other theories, such as Contagion theory (the theory of the transmission of deviant behavior in organizations) or percolation theory in social networks, to demonstrate a broader scientific basis for the use of ABM. (3) State the limitations of previous approaches as well as the challenges of new approaches fairly. For example, the author could acknowledge: “The fraud triangle paradigm is simple and lacks systems considerations, but even the complexity approach faces validation challenges.” This way, readers see that the author is aware of the full context, rather than simply pushing his own perspective.

Research Method (Agent-Based Modeling)

This study uses Agent-Based Modeling (ABM) with NetLogo 6.4.0 software to simulate organizations of varying sizes. The model design is based on the Davis & Pesch (2013) model, adopted in its entirety in its core part: Each agent represents an employee, and will commit fraud if and only if three binary conditions are met simultaneously (having an opportunity, a motive, and an attitude that supports fraud). These three factors are considered analogous to the elements of the fraud triangle (opportunity, pressure, and rationalization). At the start of the simulation, each agent is randomly assigned a status of 0 or 1 for each of these three variables (with a probability of 0.5 for each). With this random initialization, the expected value is approximately 12.5% of employees begin the simulation in an “active fraud” state (because 0.5×0.5×0.5 for each of the three variables).

Next, the model incorporates an internal social network mechanism. Each agent has a number of connections with other agents that represent informal relationships in the workplace (colleagues, acquaintances). This network is randomly generated with a certain average degree (number of connections per agent) called the target degree. The network serves as a medium for the dissemination of behavioral norms: it is through these connections that social influence occurs. The simulation is run in discrete time steps (ticks) of 10,000 ticks per trial, intended to allow long-term patterns to emerge. There is no concrete time value per tick (not seconds or days); ticks simply represent moments of interaction between employees. Every tick, each agent will

"observe" one neighbor in its network at random and see whether that neighbor has a pro-fraud status (attitude = 1) or not. The agent then has the potential to imitate the behavior of its neighbor: if the neighbor is pro-fraud, the agent can adopt a pro-fraud attitude; conversely, if the neighbor is not fraudulent, the fraudulent agent can return to honesty. This imitation probability is different for each agent, denoted q, and is drawn from a distribution. The average q in the population is set as a scenario variable: the study tested two extremes of q_avg, namely 0.15 (very low, meaning the agent is likely to be unaffected by social influence) and 0.95 (very high, the agent is almost certain to imitate the behavior of the neighbor). **The q value of each agent is considered**remains constant throughout the simulation (does not change over time and there are no other external influences). **This influence rule is symmetrical: if a fraudulent agent meets an honest neighbor, the agent has a probability q of abandoning the fraudulent attitude (becoming honest); conversely, an honest agent who meets a fraudulent neighbor has a probability q of becoming pro-fraud.** Important: Although attitudes can be contagious, an agent who turns pro-fraud will not actually commit fraud unless he also has the opportunity and motivation (opportunity & motive = 1) So, a person can be infected with a bad attitude but not take action, unless other factors happen to support it.

The model records three main outputs each run: (1) Peak fraud – the maximum number of agents in fraud status (all three factors = 1) at any point in time within 10,000 ticks; (2) Average fraud per tick – the average prevalence of fraud per tick during the simulation; (3) Fraud volatility – the standard deviation of the number of fraudulent agents over time. Each experimental scenario was run 5 times (5 independent runs with different random seeds) to see the stability of the pattern, so that the total simulation was 6 regimes × 8 sizes × 5 replications = 240 runs.

The study designed six experimental regimes (R1–R6) with the following combinations of factors:
- Two levels of susceptibility to influence (susceptibility to social influence): low (q_avg = 0.15) vs high (q_avg = 0.95)

- Three patterns of scaling internal network connectivity: - $\alpha_k = 0$ (static): A sparse network that does not increase in density as the organization size increases. All company sizes have a fixed average number of connections (constant target degree, e.g., ~8 average connections per agent) - $\alpha_k = 0.5$ (moderate growth): The larger the organization, the more connections agents can have, but at a sub-linear rate (square root of size, due to the exponent of 0.5). - $\alpha_k = 1$ (high growth): The larger the organization, the denser the network becomes linearly (average number of connections ∝ size). To prevent unrealistic scenarios (everyone knows everyone in a giant organization), researchers set an upper limit of 40 connections per agent. This means that even if the formula degree(N) = base_degree × (N/ref_size)^α_k is applied, the actual value of degree is limited to a maximum of 40.

The combination of the above variables produces 6 regimes: R1 (low q, α_k=0), R2 (low q, α_k=0.5), R3 (low q, α_k=1), R4 (high q, α_k=0), R5 (high q, α_k=0.5), R6 (high q, α_k=1). In addition, the size of the organization (N) was varied: 50, 100, 200, 400, 800, 1,600, 3,200, 6,400 agents. This range represents small to large companies. Davis & Pesch previously did not change the size (fixed at 100 agents), so scaling analysis is possible here.

The simulation data were then analyzed using log-log linear regression to obtain the scaling exponent (β). For each regime, the researchers regressed ln(output) (peak fraud, average fraud, volatility) against ln(N) (organization size). The obtained β coefficient indicates whether fraud increases sublinearly (β < 1), linearly (≈1), or superlinearly (β > 1) with respect to the growth in size. Furthermore, in order not to assume one scale pattern applies across the entire range, the authors also performed separate log–log regressions on the three size groups (small, medium,

large). This banded analysis step is to detect the possibility of local non-linearity, for example, changes in scaling behavior in a closed medium-sized organization when all sizes are combined.

Critique of Assumptions & Limitations: The ABM methodology used greatly simplifies organizational reality. Key criticisms include:

- **Simple Agent Representation:**Using binary variables of opportunity, motive, and attitude to define fraudulent behavior is an extreme reduction. In the real world, “opportunity” and “motivation” do not exist as static yes/no values; they are continuous and dynamic. For example, fraud opportunities can change with job rotations or the implementation of new controls, and motivation can increase when financial pressures arise. In the model, agents who initially lack motives are assumed to be permanently unmotivated to cheat, regardless of evolving circumstances. This is an unvalidated assumption and deviates from reality, where motives can emerge suddenly (e.g., a personal financial crisis) and opportunities can be created or lost. Consequently, this simplification means the model may fail to capture important scenarios—for example, fraud that occurs due to a new motive emerging midway through the process would never occur in the simulation, because each agent's motive is locked in place from the start.

- **No Detection/Enforcement Mechanism:**This model completely ignores the organization's response to fraud. In the simulation, if an agent meets all three conditions, they "commit fraud," and there are no consequences—they continue to be fraudsters until they change their behavior due to peer influence. In reality, organizations have systems for detection, audits, and punishment. Fraudsters can be fired or criminalized, which removes them from the "population" of perpetrators. By ignoring detection/punishment, the model allows the number of fraudsters to fluctuate freely without any permanent reduction due to intervention. This tends to result in higher volatility than in the real world, as in reality, fraud waves often stop when cases are discovered. This assumption is questionable—perhaps the authors deliberately focused on "if undetected, what are the internal dynamics?", but for practical purposes, it reduces the relevance of the results.

- **Random & Homogeneous Social Networks:**The authors model employee friendship networks as random graphs with a certain average degree. This simplifies the actual hierarchical and segmented organizational structure. In the real world, employees tend to be more connected to their teams/sections, managers have connections to subordinates, etc. A realistic network would be clustered or scale-free (some people are highly connected, most are only slightly connected). A random model can result in a statistically even distribution of connections, where anyone can be friends with anyone—this is less realistic. For example, in a random model, a junior staff member could be directly connected to a leader in another department, whereas formal structures rarely allow for such intense interactions. The absence of communities or hierarchies also eliminates the potential for detecting local fraud rings. This assumption of network homogeneity has not been validated with any organizational data, making it a significant weakness. Recommendation: models should consider small-world networks or communities to better approximate organizational reality.

- **Arbitrary Connectivity Scaling Scheme:**The author's approach sets the average degree according to the formula degree(N) ∝ N^α_k with three values of α_k (0, 0.5, 1) While it's an interesting idea to examine the effects of density, the choice of exponents of 0.5 and 1 seems arbitrary. The authors don't provide an empirical basis for why 0.5 (the square root) is considered "moderate." Are there any studies showing that every time the number of employees quadruples, the average number of relationships doubles? Without real-world

organizational data, this is purely an exploratory assumption. Furthermore, the 40-connection cap introduces artificial nonlinearity: for example, in a scenario with α_k=1, organizations above a certain size reach degree 40 and cannot grow denser. This means that at large sizes, α_k=1 effectively becomes α_k<1 (connectivity growth slows due to saturation). This 40-connection cap is a subjective choice—why 40, not 20 or 100?—and has not been justified. It may be assumed that humans are unlikely to have close relationships with more than ~40 coworkers, but it's worth citing the literature (e.g., Dunbar's number theory of ~150 relationships, etc.). The implication of this assumption is that simulation results for the high-connectivity regime may be influenced by this technical decision (a fully connected network is reached before the maximum N). This could cause the scaling pattern in very large organizations to become sublinear again simply due to model limitations, not real phenomena.

- **Extreme Social Parameters Only:**The model only tests q_avg 0.15 vs. 0.95 as a comparison of low vs. high social influence. This is an extreme choice that may simplify the analysis, but sacrifices understanding of the middle ground. According to Davis & Pesch, a critical threshold of around q ~0.3 separates stable vs. fluctuating dynamics. By choosing 0.15 (well below the threshold) and 0.95 (well above), the authors skip analysis in the transition zone (0.3–0.5), which is particularly interesting for understanding critical points. This decision may be due to computational time constraints or a focus on sharp contrasts. However, the study does not answer, for example, what happens to large-scale fraud behavior if the organizational culture is moderate (neither strong nor weak)? In other words, is the scale effect only significant at the extremes of highly ethical versus highly permissive cultures? This is an unanswered question. Suggestion: Ideally, the method should be complemented with sensitivity analyses at different q values to map the transition zone.

- **Less Realistic Initialization:**Choosing a probability of 0.5 for each motive, opportunity, and attitude means that at the start of the simulation, an average of 1 in 8 employees has committed fraud. 12.5% of employees commit fraud from the start. This is a very high figure compared to the real world – in healthy organizations, the prevalence of active fraud is typically below 1%. The authors don't provide a reason for not using a more realistic figure (perhaps to avoid too many "zero fraud" simulations at the start). Consequently, the model may tend to overshoot (because the starting point is already quite high). If in reality most organizations start in a "quiet" state (very little fraud), the effects of contagion may be more limited. The authors also don't report experiments with different initial states (e.g., only 1% fraud at the start) – this is a critical gap, as the model's robustness to the initial assumptions remains untested.

- **Limited Number of Repetitions:** Only five trials per scenario are considered a minimum. Given that this model is stochastic (random), five trials may not be enough to ensure that the average output has converged. Furthermore, outputs such as peak fraud can be heavily influenced by outliers (e.g., one run happens to produce a large fraud outbreak, while others don't). Ideally, more replications (dozens) or confidence interval calculations are provided. Without these, it's unclear whether the pattern differences between regimes are significant or simply a coincidence of a few runs. This relates to the data analysis in the following section.

Consistency & Validity of Method: Despite the above criticisms, the method is consistent with the research question: it clearly incorporates key variables (N size, connectivity, social influence) into the model. The use of ABM is indeed well-suited to exploring generative mechanisms from

the perspective of critical realism advocated by the authors – ABM allows for the observation of emergent behavior that is difficult to investigate with limited field data. Conceptually, the advantages of ABM are exploited: the authors can “turn on” or “turn off” factors (e.g., comparing α_k=0 vs. 1) to see their impact, something that is difficult to do in the real world. This model also maintains the core logic of the previous model (Davis & Pesch), thus providing continuity in the literature.

However, the external validity of this method depends on the similarity of the patterns to reality. Because the authors did not validate the model against any empirical data (e.g., there was no parameter calibration to ensure the simulation results mimic actual fraud statistics), this method is more of a theoretical exploration. A skeptic might say, "Your model is truly a toy model—it's too simple, so any results won't necessarily be relevant to fraud phenomena in real companies." This criticism is valid, given the many assumptions mentioned above. The authors do acknowledge that empirical data on fraud is partial and biased (because it only includes what is detected). thus ABM is used as an alternative to uncover latent mechanisms. While this argument is valid, it is important to note that the model's limitations must be honestly acknowledged.

Method Improvement Suggestions: In the future, the model can be gradually expanded to be more realistic: - Non-binary & Dynamic Variables: Include fraud severity or loss amounts, so that the output is not only a count of people but also financial impact. Allow the agent's opportunity or motive to vary over time (e.g., through a periodic random distribution or influenced by external factors such as company performance). - Real Organizational Structure: Use a more realistic network topology, such as a small-world network with departmental clusters, or real company communication network data (if available) to mirror the structure. The authors themselves recommend this as a further research. - Detection/Punishment Mechanism: Add a rule that a fraud agent has a chance of being detected every tick, then being removed (fired) or reset to 0 (if the punishment interpretation is deterrent). This will create new dynamics (new members replacing those fired, etc.) and see if the system continues to produce similar patterns with control interventions. - Richer Parameter Experiment: Perform sensitivity analysis with various q_avg values (instead of 0.15 and 0.95, try 0.3; 0.5; 0.7) to map how the transition from stable to unstable depends on size. Also, try different initial conditions (e.g., initially 1% fraud) to see if the initial percentage only affects the time to steady state or changes the long-term fraud level. - Number of Simulations & Statistics: Increase the number of replications or use statistical approaches (e.g., ANOVA) to assess the influence of each factor (size, q, α_k) and their interactions. Since the model is full-factorial, it should be possible to analyze it with a 2×3×8 ANOVA or non-linear regression. The author only performed regressions per regime, whereas an integrated analysis could have demonstrated the significance of interaction effects (e.g., the effect of size depends on α_k and q). - Qualitative Empirical Validation: Although it is difficult to obtain complete fraud data, the model can be validated qualitatively. For example, compare the simulated pattern with the pattern reported by internal auditors: do medium-sized companies sometimes experience more severe fraud spikes than larger companies? Finding real-life case studies (e.g., scandals in medium-sized vs. large companies) can be a check on the consistency of the model results with reality.

With the above improvements, the ABM methodology will become more credible and the model findings more reliable as a reflection of real-world phenomena, not merely a simulation artifact.

Data Analysis and Results

The authors report that across all scenarios, the simulation results do not exhibit a simple linear scaling pattern. Instead, fraud, as a model output, exhibits non-linear, scale-dependent behavior, often leading the system to become unstable (fluctuating) at certain sizes. No single, universal scaling exponent was found that applies across the entire range of organizational sizes..

Comparative analysis (per size group) revealed the existence of different behavioral phases: there are scale intervals where the increase in organizational size results in sublinear fraud growth (slower than the growth in employees), in other intervals linear, and in other intervals superlinear (faster – meaning the risk of fraud increases more rapidly than the increase in the number of people). A "volatile" phase was also found, where the number of fraud perpetrators was unstable and fluctuated significantly. This multi-phase pattern is masked when the data is analyzed in aggregate – global regression tends to hide these non-monotonic patterns.

More specifically, organizational size interacts with network and cultural factors. The authors note that scale and culture (here, culture refers to the level of social influence q_avg) can "drive fraud levels in unexpected ways.". Medium-sized organizations appear to be most vulnerable to superlinear fraud growth when their internal networks are dense and their culture is permissive (high q) In other words, the simulations found a disproportionate spike in fraud rates in mid-sized organizations with high connectivity and attitudinal uniformity. This indicates a kind of tipping point: companies are too large to have the same level of informal oversight as small companies, but not yet large enough to have the same level of formal bureaucracy/control as large companies—a combination of which leads to a fraud boom. Meanwhile, in very large organizations, while the absolute number of frauds is higher, the rate of increase may slow or the pattern may change. For example, there may be a repositioning (regime shift) where, after a certain scale, additional employees actually increase complexity, hindering the spread of fraud (because large organizations tend to be segmented, not as "cohesive" as medium-sized ones). While the authors don't describe it in those terms, their findings point to this interpretation: there is no single scaling law, but rather several subtle phase transitions depending on network conditions.

Additionally, internal network density and social influence proved to be key factors. Regimes with dense networks (α_k=1) and high q resulted in high volatility in the number of fraudsters. Fraud in these environments occurs in waves: it can suddenly spike (high peak intensity) and then decline, rather than remaining stable. The authors emphasize the importance of measuring volatility and peaks in this way, rather than simply the average incidence, as these metrics are emergent properties of the system that can be missed by simply looking at total cases.. As an example of the implications: two companies may both experience 100 fraud cases per year (the same average), but if one experiences 100 cases evenly distributed (±8–9 cases per month) while the other experiences 100 cases that mostly occur in the last 2 months of the year (an explosion of cases), the risks are different. A rapid explosion can "overwhelm the control system" because it occurs simultaneously. The authors' model shows scenarios where intertemporal variability increases with certain factors – something that is not apparent when comparing only annual totals.

The authors also state that their findings support the critical realism approach, where generative mechanisms (internal interactions) form patterns that cannot be predicted solely from individual traits.. Two experiments with the same individual parameters but different sizes and structures can produce very different aggregate fraud results – this confirms that the system context determines the outcome.[44].

Criticism of the Analysis: Although the reported results sound reasonable and are in line with the authors' theoretical expectations, there are several weaknesses in the data analysis:

- **Lack of Statistical Significance:** The results report primarily phenomenological patterns (linear/sublinear/superlinear) without any formal statistical information. With only 8 data points (measures) per regression, the resulting β coefficients may have wide confidence intervals. Furthermore, when the authors subdivide the data into 3 size groups (small, medium, and large), each group contains ~2–3 points—statistically insufficient for robust

regression. Even if calculated, the slope of the 3 points essentially only reflects the difference between the smallest and largest points in that group. This means that results like “linear vs. superlinear phase” could be artifacts of the choice of group boundaries. For example, if “medium-sized organizations” were defined differently, the slope might change. The authors do not explain how they established the small/medium/large group boundaries—based on specific numbers or simply dividing the eight sizes into three segments. Without justification, the analysis seems somewhat exploratory and post-hoc (“seeing a pattern in the graph, then subdividing the groups to highlight that pattern”).

- **No Inter-run Variability Reported:** In randomized simulations, it's best to report the variance between replicates. Is the mentioned pattern (e.g., a superlinear R6 in the middle) consistent across all runs or due to 1-2 outlier runs? The authors don't provide error bars or standard errors in our citations. Without these, it's difficult to assess the reliability of the results. One scenario might be sublinear on average, but some runs might be superlinear. Especially with high volatility, peak fraud can vary. Ideally, the analysis should include a confidence interval or p-value for each reported exponent. Without these statistics, the claim "there is no universal scaling exponent," while qualitatively correct, lacks strong assertion.

- **Less In-depth Interpretation of Causes:** The authors identify a pattern (linear/sub/super) and label it a U-shape or inverted-U, similar to D&P. However, the analysis does not delve into why this pattern occurs in the model. For example, why does a medium size model with high q and high α_k produce superlinearity? Is it because at sizes of ~400-1600 agents, the network is large enough to form a giant component, allowing fraud infection to spread to nearly all agents (peaks approaching 100%), whereas at 50-200 agents, this does not occur? The authors do not elaborate on the specific mechanisms behind the pattern, even though ABM allows for more detailed inspection (e.g., tracking the distribution of fraud cluster sizes, the duration of fraud episodes, etc.). Without such a mechanistic analysis, the results are descriptive. For complexity scientists, there should be interest in the potential for bifurcations or phase transitions. In the text, D&P concludes that there are no sharp bifurcations (gradual changes).[45]Is this also the case with this new model, or is there any indication of a tipping point? The authors state that the transition they found is also a gradual shift, not a discrete bifurcation, but the detailed discussion is minimal.

- **No Comparison with Empirical Data:** It's difficult, but it would be interesting if the authors could compare the model's output trends with empirical evidence (if any). For example, they found that in the worst-case scenario, fraud grows superlinearly with size (meaning, the fraud ratio per employee increases for larger companies). Does fraud survey data show something similar? Some reports might suggest that fraud losses per employee tend to increase in medium-sized organizations (because smaller organizations are relatively safe, while larger organizations have control). While official data is limited, a little comparison would provide confirmation or at least a plausibility check. Without it, readers must accept this simulation pattern as merely a possibility.

- **Volatility vs Average:** The author correctly highlights the need to measure volatility. However, a quantitative analysis of volatility also needs to be discussed. Does volatility (standard deviation) tend to increase with N for all regimes, or only certain regimes? Is there a critical scale at which volatility spikes? For example, perhaps volatility is low at R6 for N=50-100, but above N=200 it suddenly spikes. Presenting some kind of graph of volatility vs. N per regime would be very informative, but we don't see that detail.

Therefore, unfortunately, the analysis doesn't fully explore the insights of this volatility measure beyond general statements.

- **Effect of Connectivity Limit 40:** As mentioned in the method critique, the 40-connection cap may affect the results in the α_k=1 regime. If base_degree is set to a small N, in large organizations (>500 employees), all agents already reach 40 connections (the maximum capacity). This means that increasing from 1000 to 6000 employees does not increase the average connectivity any further. This could explain why superlinear growth is only observed at medium levels (at medium levels, density increases sharply towards a maximum; at large levels, density stagnates). The authors do not address the effect of this cap. Without additional analysis (e.g., running without the cap as a control experiment), it is difficult to know whether the nonlinear pattern is partly due to the 40-connection cap decision.

Skeptic's Counter-argument: Skeptics might argue that the results are not surprising: “Non-linearity of scale” is commonplace in complex systems. Larger organizations mean more interactions, and therefore a greater potential for behavioral outbursts (good or bad)—it's almost a tautology. So, while the model provides specific numbers, its conceptual insights can be considered “expected.” Skeptics might also ask: is this linear vs. superlinear distinction of practical significance? If larger organizations also have more control, perhaps the actual outcomes in the real world remain linear (as control balances out network effects). Without accounting for formal control factors that typically increase in larger companies (e.g., a stronger internal audit department), the simulation patterns can be misleading.

Analysis Suggestion: To strengthen the analysis, authors should: (1) Present complete tables/regressions including standard errors for the scaling exponents in each regime and band. This way, readers know how certain the “superlinear” statement is – e.g., β=1.2 with $p<0.05$, etc. – and so on. (2) Add visualizations – log-log graphs of output vs. N for several key regimes. Visualizations will clearly show where the curve bends or changes slope. (3) Use change-point detection methods instead of manually splitting the groups. There are statistical techniques to detect non-linearity (piecewise regression, spline). This is more objective than assuming three static groups. (4) Analyze internal mechanisms: for example, calculate the time autocorrelation of fraud rates to see if there is a periodic cycle; examine the distribution of the duration of high fraud episodes; or examine the profiles of several extreme runs to identify path-dependent scenarios (perhaps there are runs where fraud is extinct vs. runs where fraud is dominant, even if parameters are the same). This in-depth analysis will provide an understanding of the “why” behind the “what” emerging from the data.

With a more rigorous analysis, the conclusion that organizational fraud exhibits complex patterns would be more convincing than simply a qualitative observation. Given the academic audience, transparency of data and statistics is crucial for credible findings that can be replicated by other researchers.

Discussion

In the discussion, the authors interpret the results as evidence that fraud against organizations is an emergent property of complex systems. They reaffirm the theoretical argument: these findings strengthen the complexity-theoretical critical realist proposition that fraud arises from the interplay of interacting mechanisms in an open system, rather than the dispositions of individuals alone. This means that employee fraudulent behavior cannot be separated from the context of the organization's structure, culture, and surrounding interactions. This challenges the validity of traditional anti-fraud strategies that focus too much on "rogue" individuals or rely on linear

assumptions (e.g., 2 employees = 2 times the risk). The author argues that the scale and culture of an organization can trigger disproportionate levels of fraud (unexpected ways) For example, an internal network that is too dense plus a permissive culture can make a company vulnerable to a spike in fraud. The finding of a superlinear phase in medium-sized organizations (in the simulation) serves as an example that companies of a certain size may face a greater risk of fraud "explosion" relative to their size.

The authors then draw practical implications: they argue that the effectiveness of conventional approaches, such as anti-fraud training featuring case studies of perpetrators (in the hope of deterring them), may be limited or even counterproductive. Why? Because seeing numerous examples of fraud can inadvertently normalize the behavior in employees' minds ("other people cheat too"). Similarly, the authors criticize data-driven solutions (forensic analytics based on known fraud patterns) as a closed feedback loop: such detection models will only find similar cases that have already occurred, so that innovations in new fraud schemes or latent risks that have never appeared before will be missed. He said this does not touch on the causal mechanisms in the Real domain (using the term critical realism). Instead, the authors advocate a systems approach: "mapping the affordances of the fraud field" – mapping the structural factors within the organization that are prerequisites for the emergence of fraud opportunities. With this mapping, interventions can be directed to the root causes (e.g., improving incentive structures, reducing unsupervised areas, etc.) before the combination of factors creates opportunities for fraud.

The authors also highlight that anti-fraud performance measurement should not simply look at the average number of incidents. If management only sees "5 cases this year, 5 cases last year" (stable), they could have a false sense of security, even though the model indicates the potential for latent volatile episodes. Volatility and peak intensity measures need to be considered—for example, monthly or quarterly variations in incidents, not just annual totals—to detect whether a system is approaching a critical threshold. Essentially, snapshot metrics can obscure latent potential for escalation.

Criticism: The author's discussion is conceptually rich, but there are several things that deserve criticism:

- **Overemphasis on Critical Realism:** The author strongly emphasizes the critical realist interpretation. Terms such as epistemic fallacy appear, namely the assumption that relying on empirical data (which is limited to detected cases) will always fail to understand the full reality. For practical or positivist readers, this section may be considered too philosophical and not down-to-earth. The key message is simple: recorded fraud data is the tip of the iceberg, so we need other methods (models, qualitative investigations) to understand the hidden parts. It would be more effective if discussed more directly without heavy philosophical jargon. The impression is that the author is trying to frame this study as a paradigmatic revolutionary step, when up to this point, what has been done is an exploratory simulation with real limitations. A reviewer might feel that the author is slightly over-claiming theoretical significance without sufficiently humbly discussing the limitations of the model.

- **Lack of Recognition of Model Limitations:** The discussion barely addresses the specific weaknesses of the model outlined in the methods. For example, there are no statements like "of course, our model simplifies things like X and Y." This is a significant gap. The discussion section should not only outline the implications of the results but also the limitations of the study. The author may feel that including these would weaken the narrative; however, without mentioning limitations, a critical reader could judge the author

as insufficiently reflective. For example, the discussion could state: "These results were obtained in a closed simulation without incorporating fraud detection, so direct implications for the real world require caution." Without such language, there is a sense of jumping to conclusions.

- **Generalization to Practice:** The recommendations (mapping affordances, paying attention to volatility, etc.) remain at a general conceptual level. The discussion doesn't provide concrete examples of how an organization can apply these insights. For example, how can we practically "map the affordances of the fraud field"? Is it through a comprehensive risk audit, an employee culture survey, or an analysis of internal communication networks? Reading such advice might confuse practitioners. Similarly, it says dense networks + high influence = high risk, but the mitigation steps aren't elaborated. Does this mean companies should limit informal employee interactions (clearly unrealistic), or ensure a strong ethical culture before the organization grows (a possible insight, but not explicitly stated). The lack of translating the results into actionable guidance makes the practical contribution of this study somewhat abstract.

- **Not Relating to Real Case Studies:** The discussion would have been stronger if it had connected the results to real-life examples. For example, it could have been stated, "The phenomenon of fraud explosions in mid-sized companies was seen in scandal X, where massive fraud occurred in a short period of time." Linking to real-life examples would have demonstrated relevance. Unfortunately, the authors don't do this. The literature on organized crime or corporate fraud may have relevant case studies (e.g., the Enron scandal can be viewed from the perspective of a strong but distorted culture).

- **Skeptical Counter-arguments to Implications:** The author argues that individualistic strategies are inadequate. Skeptics might counter: Individual-focused strategies (such as employee screening and personal ethics training) are indeed limited, but not useless. The proposed systemic approach is also not easy to implement—understanding latent causal mechanisms requires in-depth investigation, and each organization is unique. Furthermore, traditional controls such as segregation of duties, internal audits, and so on, while considered "symptomatic," have been proven to prevent many practical frauds. Therefore, don't be too quick to discard old approaches; instead, complement them with new perspectives. The author's discussion falls into a false dichotomy: it seems as if individual versus systemic approaches are mutually exclusive. The best solution, however, may be a synthesis—improving culture and systems while still taking individual action. This point is underappreciated.

- **Over-Generalizing Model Findings:** The authors take the model results (e.g., medium-sized organizations with high connectivity have a high risk) and present them as general reality. This is problematic because the model hasn't been validated. The discussion notes that "organizations with dense internal networks and a culture of high influence appear vulnerable to disproportionate fraud risk."[49]. While it sounds logical, the word “appear” should be followed by “in our simulations.” Without that clarification, readers could assume this is an empirical finding.

Alternative Approaches/Thoughts: If the author wanted to strengthen the interpretation of the results, they could use a classical organizational sociology perspective. For example, consider the concept of "small firms: high interpersonal oversight -> suppresses fraud; large firms: high formal control -> suppresses fraud; medium firms: too large for interpersonal oversight, too small for strong formal control -> prone to fraud." This explanation is less jargon-ridden, but it aligns

with the model's results. By linking it to existing theories (perhaps a theory of the optimal size of bureaucracy versus internal crime), the discussion will be more grounded.

The authors could also discuss how a strong culture can have a two-pronged impact: high cultural coherence in a large organization can be positive if the norms are ethical, but if it happens to shift negatively, the effects are massive (fraud is rampant). This is actually implied in the results (high q + high connectivity = danger). So a recommendation might be: build a strong culture of integrity before the organization reaches scale, as reversing a bad culture in a large organization is much more difficult. Unfortunately, such a point is not clearly articulated.

Suggestions for Discussion: 1. Provide a "Limitations" paragraph. Explicitly state the model's limitations and implications: e.g., "Our findings are hypothetical, simulation-based. While the emerging patterns are consistent with complexity theory, the model's limitations (e.g., factor X has not been modeled) mean caution is needed before generalizing to all organizations." This demonstrates the author's critical scientific approach to his or her own work. 2. Offer ways to implement the results. For example, "Practitioners can apply these findings by conducting organizational network analysis (identifying key employees who connect units) and ensuring ethics programs focus on these nodes to prevent the normalization of misconduct." Such suggestions will make the discussion more concrete. 3. Compare with conventional insights. Explain how this new view complements existing knowledge. For example, "These results do not mean we should abandon individual screening (the fraud triangle), but they do suggest that systemic factors (networks and culture) create the conditions in which the fraud triangle can manifest itself extensively. Therefore, effective prevention needs to target both levels: the individual and the system." This balance prevents skeptical readers from dismissing the new idea entirely. 4. Review the literature through the lens of the results. Are these findings consistent with Kim & Xiao's (they found model-data alignment in welfare programs)? Do they support Button's argument (fraud field equilibrium)? A discussion of the literature will further clarify the study's contribution.

With the steps above, the discussion will be more critical, balanced, and help readers understand the value and context of the limitations of this research's scientific contribution.

Conclusion and Recommendations

This study concludes that the level of fraud in an organization is not a linear quantity that is easily predicted from the number of employees alone, but rather an emergent phenomenon that is influenced by the complex interaction between size, network structure, and organizational culture. There is no single "law of scaling" – fraud can grow slower, in proportion to, or faster than the growth of the organization depending on internal conditions. The main finding confirms that fraud is not simply the accumulation of individual actions, but arises from the interplay of multi-level mechanisms (e.g., many individuals with weak motivations can become a major problem if they influence each other in an open system). In other words, the systemic context determines whether an individual's potential for fraud will be realized massively or not.

Based on these scientific conclusions, the authors recommend a paradigm shift in anti-fraud efforts. Practitioners are advised not to focus solely on average or total cases, but also to be aware of the volatility and peaks of fraud incidents as indicators of system health. The author also suggests that developing a fraud control strategy should include a comprehensive mapping of the organization's "fraud field" – that is, identifying latent factors such as control gaps, areas prone to collusion, influential employee groups, and prevailing informal norms. With this mapping, management can make proactive interventions at the system level (e.g., changing business

processes that give too much authority to one person, breaking up internal networks that are too siloed, or transferring key personnel to break the chain of negative influence).

In addition, the authors recommend a future research approach that combines modeling with empirical data. They acknowledge that actual fraud data is always partial (because only cases are detected), but views the integration of ABM and empirical studies as a solution. It is recommended to conduct simulations with real organizational network structures and test various intervention scenarios in such virtual environments.The results of such simulations can then be carefully validated by comparing the simulation patterns with empirical data (e.g., incident patterns from audit reports) and supplemented with qualitative research (interviews, ethnographic studies) to uncover mechanisms not apparent in the quantitative data. This step is believed to deepen the understanding of fraud as a complex phenomenon while supporting the development of more effective system-level prevention strategies. The author recognizes that because social systems are “open,” our knowledge will never be perfect; but a combination of empirical and modeling approaches can “illuminate hidden mechanisms” and gradually reduce ignorance in this area.

Critical Evaluation: The authors' conclusions are generally consistent with the results – they don't overdo it by promising absolute certainty, even acknowledging the complex nature of open systems. However, there are some critical caveats:

- **Generalization vs Context:** The conclusion confirms the emergent nature of fraud, but it should be accompanied by a contextual reminder that it is based on simulation. In the original text, the caveat "based on our model" was not explicitly stated when stating, for example, "dense networks + high influence = vulnerable." In a scientific report, this should be more explicit to avoid the impression that this is an empirical law. Fortunately, the authors immediately follow up with recommendations for further research for validation, indicating they understand these results are merely hypotheses that need further testing.

- **Traceability to Initial Destination:** The research objectives (asked in the introduction as questions) are answered in the conclusions. For example, they asked whether fraud increases proportionally with size—the answer is: not always, the scaling pattern depends on the conditions. They asked about the role of connectivity and influence—the answer is: very influential, resulting in different systemic behaviors. So, internally, the conclusions are consistent with the research questions.

- **Scientific Contribution:** The authors claim to open new avenues of fraud studies. From a critical perspective, these contributions are preliminary. They have demonstrated proof of concept that ABM can generate multi-scale emergent fraud behavior. However, until empirical validation is provided, these results are hypothetical. The contribution to fraud theory lies in supporting a multi-level perspective (Bunge, etc.)—but again, this is more of a model illustration than empirical evidence. It would be better for authors to state that their findings support a particular theoretical framework but still require testing. For example, it might be helpful to state, "This study provides preliminary support for perspective X" rather than implying that the old perspective should be abandoned.

- **Practical Recommendations:** The advice provided is sound: focus on systemic factors, monitor volatility. However, some recommendations may be difficult to understand without implementation guidance. For example, the phrase "snapshot measures risk obscuring latent escalation."That's true, but what are the concrete recommendations? Perhaps it should be added: "Therefore, management should monitor long-term trends and the distribution of cases across the organization, not just the annual total." This explanation is missing from the text, making the recommendations seem abstract.

Additional Suggestions:1. Write a Limitations section before the conclusion. This is academically important. Explicitly mention the limitations of the model (which we discussed in the methods). Examples include the assumption of static variables, the absence of external factors, etc., as well as data limitations (hidden fraud). By outlining these in the conclusion, the reader is assured that the authors have not overlooked the study's shortcomings. 2. Prioritize Further Empirical Validation. The authors have recommended mirroring real networks and comparing data. This suggestion is very appropriate. Additionally, they could recommend collaborating with companies to test-bedding this model. For example, applying the model to internal data from a large company (with adjusted parameters) to see if the model's predictions match the areas of vulnerability identified. This would bridge the model-reality gap. 3. Don't Forget the Humans: While this research is about systems, ultimately, policies must be implemented by people. Recommendations could address the importance of an effective integrity culture program. The model results clearly show that a highly contagious culture (attitude) can have detrimental effects. Therefore, strengthening the integrity culture of large companies through training, rotating leaders to ensure the company's values are shared, etc., is a practical recommendation aligned with the findings (reducing effective q_avg). 4. Alignment with Regulations: Organizations are often subject to anti-fraud regulations (e.g., mandatory internal audits for public companies). The authors could recommend that regulators address the scale factor: for example, requiring mid-sized companies to have adequate audit/investigation functions due to their vulnerability. This public policy recommendation would demonstrate the broad impact of the findings.
