# Supplementary material for: Organisational scale and fraud volatility: An exploratory agent-based simulation of occupational fraud dynamics
Source: PLoS One. 2026 Jul 17;21(7):e0342964. doi: 10.1371/journal.pone.0342964 (PMC13378982; doi:10.1371/journal.pone.0342964)
Supplement: Attachment — Submitted filename: REVIEW_PONE-D-25-65420_R1.pdf [file pone.0342964.s004.pdf]

Review PONE-D-25-65420_R1

1. Substantive Summary of the Study

This study positions fraud as a systemic phenomenon arising from the interaction of agents within the organization, rather than merely the sum of individual actors' decisions. The model operationalises a simple variant of the fraud triangle (motive, opportunity, attitude) in the form of binary variables that, when met simultaneously, trigger fraudulent actions by agents.

The authors conducted 9,000 simulations for various combinations of organisational size, social influence parameter (q), and network connectivity scaling parameter (αk) to observe the scaling patterns of fraud mean, peak, and volatility. The main results show that the average fraud and fraud volatility tend to increase superlinearly with the size of the organization, while the peaks of fraud tend to scale nearly linearly with the size of the system.

2. General Evaluation and General Recommendations

Substantively, the article addresses a relevant and relatively new topic at the intersection of economic criminology, organisational fraud studies, and complexity models.The article offers an important contribution by illustrating that organisational structural factors (scale, interaction networks, attitude contagion) have the potential to produce fraud patterns that are disproportionate to the size of the organization.

However, the article has several major weaknesses related to the realism and justification of the model, the absence of strong validation procedures, over-extension of philosophical discussions, and the consistency of the strength of the results' claims compared to the model's limitations. Based on that, the main recommendation is Major Revision before the article is deemed acceptable for PLOS ONE.

3. Evaluation of Each Stage of the Article

A. Title

Weakness

The phrase "evidence from an agent-based model" has the potential to give the impression that the results carry empirical weight comparable to observational or experimental data-based studies, whereas this study is exploratory and based on a highly simplified model.

The term "occupational fraud" in the title is not well-supported by modelling that substantively distinguishes the type of fraud or the context of organisational roles, as the agents in the model are homogeneous and only have a binary fraud status.

Suggestion

The author should modify the title to explicitly indicate the exploratory and simulated nature, for example by adding the words "simulated" or "exploratory modelling". The authors should more explicitly relate the term "occupational fraud" to the model structure (for example, explaining that the model represents embezzlement or certain internal misappropriation at the level of trusted individuals).

B. ABSTRACT

Weaknesses

The abstract does not explicitly state that this model is highly simplified, using binary assumptions and random networks, and is not calibrated to empirical data, so readers might interpret the results as if they were pure empirical estimates. The use of technical terms such as "superlinearly" and "volatility" without intuitive explanations may make it difficult for readers who are not modellers or scaling experts to understand the practical implications of the results.

Suggestion

The abstract should include an explicit sentence marking the study as an exploratory simulation based on a conceptual model, not an empirically-calibrated model. The abstract should provide a brief, more intuitive explanation of the main technical terms (for example, "the average fraud grows faster than the growth in the number of employees" as a translation of "superlinearly").

C. Introduction

Weaknesses

The introduction is too lengthy and contains several repetitions of arguments (for example, about the limitations of detected data, the need for a systemic perspective, and the role of complexity science), making the main focus less sharp. The formulation of specific contributions and research objectives is still mixed with general discourse, making it difficult for readers to clearly identify what is truly new and different compared to previous ABM fraud studies.

Suggestion

The introduction should be condensed by eliminating repetition and moving the more general theoretical discussion to the literature review section. The author should add an explicit paragraph detailing the key contributions of the article in bullet points (for example, three main contributions to theory and method).

D. Theoretical Review and Fraud Literature

Weaknesses

The theoretical review is too broad and delves deeply into several aspects (e.g., the history and controversies of the fraud triangle, detailed interpretation of Cressey) that are not entirely necessary to justify the model design.

The direct relationship between previous empirical findings on organization size–fraud and technical choices in the model (size range, social parameters, network shape) is less explicitly explained, making the model appear more as a generic abstraction rather than a derivative of the literature.

Suggestion

The authors should focus the literature review on sources that are truly relevant to the model design (scaling crime studies, contagion deviance, ABM fraud, and empirical studies on organisational size). In-depth philosophical or historical discussions about the fraud triangle can be summarised or partially moved to the conceptual discussion section.

E. Theoretical Framework and Research Questions

Weaknesses

The theoretical framework is not explicitly visualised in the form of a conceptual diagram, making the boundary between the conceptual model and the computational model still unclear for non-modeling readers. The article does not propose explicit hypotheses (even non-directional) for each combination of key parameters, making it difficult to distinguish which results align with theoretical expectations and which patterns are truly unexpected.

Suggestion

The authors should include a conceptual framework diagram that connects organisational size, network structure, social influence, and fraud outcomes (mean, peak, volatility). The authors should formulate a set of explicit hypotheses, such as the expectation of superlinearity under certain conditions of connectivity and contagion.

F. Method: Agent-Based Model Design.

F.1. Model and Agent Specification

Weakness

The assumption that motive, opportunity, and attitude are initialised as Bernoulli variables with a probability of 0.5 lacks a strong empirical basis and tends to be arbitrary. The use of binary thresholds for the three components of the fraud triangle ignores the variation in the intensity of motivation, opportunity, and rationalisation, making fraudulent behaviour overly mechanistic and not closely resembling psychological and organisational reality.

Suggestion

The authors should justify the selection of initial values and the distribution of agent attributes more clearly, or acknowledge it as a pure simplification and test the sensitivity to parameter variations (e.g., 0.2 – 0.8). In future research, the authors could consider continuous or multimodal representations for attitude and opportunity to approach real heterogeneity.

F.2. Network Structure and Organisational Scale

Weakness

The network structure is generated as a random network that does not represent the typical characteristics of real organisations (hierarchy, departments, workgroups, and supervisor-subordinate relationships). The range of organisational sizes was chosen without strong empirical justification and without discussing the implications that many real organisations fall outside this range or have subunits that behave differently.

Suggestion

The authors should test at least one or two types of alternative network structures that are more realistic (e.g., small-world with department clusters and some long ties) to examine the robustness of the results.

The author should add an explanation of why the size range of 25–6,400 was chosen and how this range relates to the size of real organisations (e.g., business units or departments).

F.3. Experiments, Output, and Scaling Analysis

Weaknesses

The article has not presented regression diagnostic analysis or robustness tests (e.g., outlier checks, residual nonlinearity, or comparison with alternative fittings) which are important when claiming a power-law relationship.

The division of results into "whole range" and "banded scaling" adds complexity to the narrative but is not accompanied by concise visualisations (such as heatmaps or surfaces), making it difficult for readers to follow the patterns.

Suggestion

The authors should add basic diagnostic checks (residual plots, additional goodness-of-fit) to justify the use of the log-log linear model and the interpretation of the power law. The authors should consider compact visualisations (heatmaps of exponent scaling as a function of q and αk) to replace lengthy narrative descriptions.

F.4. Model Verification and Validation

Weakness

There is no separate section that systematically explains the verification process (whether the model operates according to specifications) and validation (whether the emerging patterns are consistent with empirical stylised facts about organisational fraud).

The article has not yet demonstrated an effort for pattern-oriented validation or at least a comparison of simulation results with known qualitative characteristics from real-world fraud studies.

Suggestion

The authors should add a subsection "Model verification and validation" that explains the steps of code verification, parameter sensitivity testing, and pattern matching with basic empirical facts (for example, the prevalence of fraud never being zero or 100 percent persistently).

The authors should relate the simulation output to some empirical patterns (e.g., the distribution of fraud losses in large vs. small companies) to provide an initial indication that the model operates within a reasonable behavioural domain.

G. Results

Weaknesses

The presentation of the results is very dense with numbers and technical terms, making it difficult for non-modeling readers to understand the substantive significance of the differences in β values between scenarios. There are formatting and layout issues with the tables (editing artefacts, duplicate rows, disorganised column formatting) at the end of the manuscript that reduce readability.

Suggestion

The authors should include more verbal interpretations linking the β values to practical consequences for the organization (for example, describing how the risk of fraud changes when the organization doubles in size under certain regimes). The authors should clean up all the result tables, ensure label consistency, and remove any remnants of formatting from word processing.

H. Discussion.

Weaknesses

The discussion allocates a significant portion to criticism of the fraud triangle and philosophical discussions, but does not fully critique the inherent limitations of the model design itself and the potential bias in scaling results. Some parts of the philosophical argumentation are repetitive and take up space that could be used for a more detailed discussion on the substantive implications of the results for fraud control design in real organisations.

Suggestion

The discussion should balance criticism of the individualistic view of fraud with a critical reflection on the simplifications and limitations of the models used. The author should condense the repetitive philosophical discussions and replace them with a more concrete discussion on how organisations can utilise insights regarding scale effects and informal networks.

I. Theoretical and Practical Implications

Weaknesses

Policy implications are still relatively general and not always directly linked to specific model parameter configurations or scenarios.
The article does not sufficiently emphasise that these implications are hypothetical and need further testing, considering that the model has not been empirically validated.

Suggestion

The author should link practical recommendations to specific model scenarios, for example, highlighting parameter regimes that produce exceptionally high volatility and explaining relevant organisational actions.

The authors should explicitly label the practical implications as "preliminary thoughts" that require confirmation from real organisational data before widespread adoption.

J. Limitations and Directions for Future Research

Weaknesses

Although the limitations are acknowledged, the language used to describe the results (e.g., "stable superlinear relationships") in other parts of the manuscript is still strong enough to create an impression of over-claiming compared to the model's capabilities.

Limitations related to the representation of organisational size and variations in formal structure have not been discussed in depth in terms of their potential impact on the generalisation of results.

Suggestion

The authors should align the entire manuscript with a strong emphasis on the exploratory and tentative nature of the results, especially in the conclusion and implications. The author should discuss in more detail how significant differences in real-world organisational structures (e.g., multi-divisional, global) can limit the direct application of the model's results.

K. Language Quality, Structure, and Format Compliance

Weaknesses

The manuscript is too long and repetitive in several segments, especially regarding the critique of the fraud triangle and the exposition of critical realism ontology, which tends to obscure the empirical and methodological focus. There are technical formatting issues in the table section and the end of the manuscript (editing artefacts, duplicate text, messy column formatting) that do not meet the resubmission manuscript standards.

Suggestion

The author should substantially shorten the manuscript (15–20%) with a focus on eliminating repetition and moving highly philosophical conceptual discussions to an appendix or a separate article. The author should conduct a thorough final technical edit to correct all tables, captions, and formatting traces before the next submission.

L. Summary of Main Strengths and Weaknesses

L.1 Main Strengths

- The topic is highly relevant and relatively new at the intersection of fraud studies, organisations, and complexity science.
- The integration of criminology literature, economic crime, and complex systems theory is quite rich and demonstrates the author's depth of theoretical understanding.
- The simulation design includes many scenarios and a variety of organisational sizes, with systematic reporting of scaling indicators.
- The simulation code and data are provided openly, supporting transparency and replication.

L.2 Main Weaknesses

- The model assumptions are overly simplified, particularly regarding the distribution of agent attributes and network structure, without adequate empirical justification and sensitivity testing.
- The lack of explicit model verification and validation procedures diminishes the strength of the claim that the discovered scaling patterns reflect generic fraud mechanisms in real organisations.
- The philosophical discussion and critique of the fraud triangle paradigm tend to be excessive, obscuring the article's concrete empirical and methodological contributions.
- There is an inconsistency between the acknowledgement of the model's significant limitations and the strong language used to describe the stability of the outcome patterns.

M. Final Recommendation.

Based on a thorough evaluation of the theoretical substance, model design and analysis, reporting quality, and the balance between claims and limitations, the review recommendation is: Recommendation: Major Revision.

The authors need to undertake the following major improvements:

1. **Clarify the epistemic position** of the research as exploratory modelling with clear generalisation boundaries, and adjust the strength of the result claims' language to the degree of available validation.
2. **Strengthen the model's foundation and evaluation** through better parameter justification, sensitivity testing, and documented verification/validation procedures.

3. **Focus and condense the manuscript,** particularly by reducing conceptual repetition and balancing the portion of philosophical discussion with substantive implications for theory, measurement, and anti-fraud policies in organisations.

If these improvements are consistently implemented, this article has the potential to make a significant contribution to the development of complexity-based approaches and agent-based modelling in the study of organisational fraud.
