# Supplementary material for: Organisational scale and fraud volatility: An exploratory agent-based simulation of occupational fraud dynamics
Source: PLoS One. 2026 Jul 17;21(7):e0342964. doi: 10.1371/journal.pone.0342964 (PMC13378982; doi:10.1371/journal.pone.0342964)
Supplement: Attachment — Submitted filename: ARTICLE - PONE-D-25-65420_R2.pdf [file pone.0342964.s006.pdf]

Translated from Indonesian to English - www.onlinedoctranslator.com

REVIEW ARTICLE PONE-D-25-65420_R2

Article Title: Scaling of Occupational Fraud with Organizational Size: Evidence from an Exploratory Agent-Based Model

PART 1: TITLE ANALYSIS

Article Title

Full Title:"Scaling of Occupational Fraud with Organizational Size: Evidence from an Exploratory Agent-Based Model"

Title Weaknesses

The title is too long (18 words in the full title), which can reduce its impact and memorability. The term "exploratory agent-based model" may be too technically specific for readers from a non-modeling background. There's no explicit indication of the superlinear scaling phenomenon, the key finding, so readers won't immediately grasp the uniqueness of this research.

Improvement Suggestions

1. Consider shortening the full title to: "Organizational Size and Occupational Fraud: Superlinear Scaling Patterns in Agent-Based Models"
2. Add the keyword "superlinear scaling" to the title to highlight the unique findings and theoretical contributions of the novel.
3. Make sure the title can be easily identified through academic search databases with keywords such as "fraud scaling," "organizational systems," and "complex adaptive systems."

PART 2: ABSTRACT ANALYSIS

Abstract Weaknesses

The abstract exceeds the usual PLOS ONE word limit (250 words maximum). The authors do not explicitly mention the focus on two key system mechanisms (social influence and network connectivity), which should have been communicated for a richer theoretical context. The limitations of the study (the exploratory nature of the model and the simulation data, not empirical) are not mentioned in the abstract, although this is crucial for methodological transparency.

Improvement Suggestions

1. Reduce the abstract length to 200-220 words while retaining essential information.
2. Add one sentence about the two main system mechanisms: "The study examined how organizational scale interacts with social influence and network connectivity."

3. Include an explicit constraint: "Findings from exploratory agent-based modeling should be interpreted as generative mechanisms rather than empirical validation"
4. Keep the quantitative results specific as they are most informative for academic readers.

PART 3: INTRODUCTION

Introduction Weaknesses

The introduction is too long (running into many pages) and the detail may be excessive for a journal with tight page limits. Some references to empirical fraud data (such as UK businesses statistics) are not integrated seamlessly with complexity theory, creating the feeling that there are two separate narratives: (1) fraud as a practical problem, and (2) fraud as a complex systems phenomenon.

The author is less clear about why specifically social influence and network connectivity were chosen as the focus mechanisms (Carter, 2020). The theoretical justification for the choice of these two mechanisms needs to be strengthened with more explicit reference to systems dynamics theory or complex adaptive systems literature.

Improvement Suggestions

1. Reduce the length of the introduction by consolidating redundant sub-points and retaining only the most relevant empirical examples.
2. Add a small subsection titled "Theoretical Mechanisms" that explicitly explains why social influence and network connectivity are critical mechanisms in organizational fraud systems.
3. Use stronger transition statements between empirical observations of fraud and theoretical frameworks of complexity.
4. Explain research questions early in the introduction, not just at the end
5. Consider adding a small figure that visualizes the theoretical model before the methodology is explained.

PART 4: LITERATURE REVIEW AND HYPOTHESES

Weaknesses of Theoretical Review

The hypotheses are not written in a clear formal format (H1, H2, H3). The author refers to these as "exploratory research questions" rather than "hypotheses," which is acceptable but does not provide specific targets for testing (Carter, 2020Some recent literature on scaling laws in complex systems and critical realism as a philosophical foundation feels somewhat underexplored. The connection between critical realism and agent-based modeling methodology needs to be justified more deeply.

The dominant literature review discusses fraud and complex systems, but the literature on organizational structure and hierarchical effects in the propagation of behavior is less developed, even though this is relevant for the interpretation of findings (Carter, 2020).

Improvement Suggestions

1. Formalizing three research questions into hypotheses with a clearer predictive direction (although exploratory, can provide directional expectations)
2. Add a more detailed subsection on scaling laws in the complex systems literature (physics-inspired power laws, universality classes) to provide a stronger theoretical grounding.
3. Explain more explicitly the philosophical foundation of critical realism and how this guides model design and interpretation.
4. Discuss in more depth the implications of hierarchical and departmental structures in real organizations, and why this model simplification is justified.
5. Add a comparative table comparing findings from different empirical studies on organizational size and fraud, showing heterogeneity that indicates the need for a modeling approach.

PART 5: RESEARCH METHODOLOGY ANALYSIS

Methodological Weaknesses

The model uses random network generation to produce heterogeneous interaction structures, but real organizations typically exhibit hierarchical reporting relationships, departmental clustering, and differentiated communication patterns (Carter, 2020). The authors acknowledge this limitation, but do not fully explore the implications of this simplification for the generalizability of the findings.

Parameter uncertainty is a significant issue. Values for parameters such as susceptibility to social influence and connectivity scaling were chosen to explore scenarios theoretically rather than calibrated against empirical organizational data (Carter, 2020This means the results should be interpreted as illustrations of how different behavioral and structural conditions can shape fraud dynamics, not as estimates of magnitude in real organizational settings.

The model focuses exclusively on fraud by internal employees, excluding fraud by customers, suppliers, or external actors (Carter, 2020). This is a reasonable scope limitation, but reduces the applicability to broader fraud phenomena.

The model does not capture the full range of conditions present in real organizational environments, including geographic dispersion, technological complexity, transaction volumes, and governance arrangements (Carter, 2020The absence of these factors can shape or constrain opportunities for fraud and the effectiveness of safeguards.

Improvement Suggestions

1. **Network Structure Enhancement:**Modify the model to incorporate more realistic organizational network structures such as small-world networks or modular networks that capture clustering, hierarchy, and long-range ties. This is particularly relevant for large organizations that are multi-divisional and geographically dispersed.Carter, 2020).

2. **Calibration Strategy:**Develop a validation strategy that includes sensitivity analyses for parameter values. Conduct interviews with fraud risk practitioners to obtain empirical ranges for parameters such as susceptibility to influence in real organizational contexts.

3. **Expanded Fraud Types:**Consider extending the model to include external fraud (customer, supplier) or collaborative fraud involving multiple perpetrators, to increase realism and scope.

4. **Hierarchical Effects:**Incorporate explicit hierarchical structures with differentiated access privileges and information asymmetries. Model how fraud opportunities and detection probabilities vary across organizational hierarchies.

5. **Comparative Validation:**Conduct comparative validation with empirical fraud data from multiple sources (fraud surveys, forensic audit reports, whistleblower databases) to verify that simulated fraud dynamics produce outcomes in the plausible domain consistent with empirical observations.

6. **Transparent Documentation:**Provide complete model documentation, parameter tables, and code availability (ideally on OSF or GitHub) to enhance reproducibility and facilitate further research.

PART 6: ANALYSIS OF RESEARCH FINDINGS

Weakness of Results

Results are purely exploratory and based on simulation, not empirical data. The authors clearly communicate this, but the gap between simulated outcomes and real-world fraud prevalence remains substantial. Some simulation runs (n=14) produced peak fraud values of zero and were excluded from the log-transformed regressions, introducing a minor upward bias in the estimated scaling relationship (Carter, 2020), particularly in regimes where fraud prevalence is very low.

Lack of external validation is a significant limitation. The model examines generative mechanisms rather than attempting to reproduce specific empirical datasets, so simulated organizations are stylized representations rather than replicas of real organizations. Organizational size in the model is represented solely by number agents, whereas real organizations differ along many additional dimensions (Carter, 2020).

Improvement Suggestions

1. **Future Empirical Validation:**Collaborate with organizations willing to share anonymized fraud data to validate whether similar scaling relationships exist in real-world settings.

2. **Sensitivity Analyses:**Conduct extensive sensitivity analyzes to show robustness of findings across different parameter configurations and initial conditions.

3. **Complementary Statistical Approaches:**Employ additional statistical techniques (eg, machine learning approaches to identify non-linearities, Bayesian hierarchical models to account for structural uncertainty) to validate power-law relationships.

4. **Scenario Analysis:**Present results in the form of scenarios (low-fraud, high-fraud, high-volatility environments) to make findings more actionable for practitioners.

PART 7: DISCUSSION

Weaknesses of Discussion

The discussion was too long and sometimes repetitive, repeating points from the results section. Some discussions about critical realism as a philosophical foundation felt tangential to the main empirical narrative about fraud scaling.

The connection between simulated findings and actual practitioner decision-making is less concrete. The discussion provides general principles but fewer actionable guidelines for specifically how fraud prevention strategies can be adapted to different organizational sizes.

The limitation discussion, although comprehensive, could be better integrated with the findings discussion rather than treated as a separate section.

Improvement Suggestions

1. **Synthesize with Practice:**Add a subsection titled "Implications for Fraud Risk Management" that translates simulated findings into specific practices (e.g., "For organizations doubling in size, detection thresholds for anomaly detection should be adjusted by a factor of 2.4 minimum").

2. **Visual Summaries:**Use summary figures or tables that consolidate key findings and limitations to enhance clarity.

3. **Comparative Discussion:**More directly compare findings with empirical fraud survey data to contextualize the realism of simulated patterns.

4. **Speculative but Grounded Future Work:**Identify specific hypothesis-driven future research that can test generative mechanisms proposed in the model.

PART 8: CONCLUSION

Weaknesses of Conclusion

The conclusions lack actionable and specific next steps for both researchers and practitioners. The general statement that "larger organizations require disproportionately stronger prevention strategies" is not specific enough for implementation.

There is no sufficient reflection on the generalizability limitations and boundary conditions to which these findings apply.

Improvement Suggestions

1. **Specific Calls to Action:**End with specific recommendations for: (a) researchers—what empirical studies should be conducted to test mechanisms; (b) practitioners—what specific fraud prevention policies or organizational redesigns may reduce fraud risk given scaling patterns.
2. **Generalizability Boundaries:**Explicitly state the types of organizations where findings are likely most applicable (eg, "findings likely most relevant for large hierarchical organizations with distributed decision-making") and which require additional research (eg, "small organizations, matrix structures, highly digitalized organizations").
3. **Bridge to Practice:**Add a concluding paragraph that connects theoretical contributions to actual organizational governance challenges and opportunities for implementing findings.

PART 9: LIMITATIONS

Weaknesses in Presentation Limitations

Although limitations were identified, the response to them was somewhat passive. The authors acknowledge but do not always suggest concrete mitigation strategies. Some limitations (such as the absence of a hierarchical structure) were proposed for future work but were not explored as sensitivity analyses in the current study.

Improvement Suggestions

1. **Limitation Hierarchy:**Rank limitations in terms of impact on conclusion validity (critical, moderate, minor) to help readers assess robustness.
2. **Mitigating Strategies:**For each major limitation, suggest a specific strategy to mitigate it within the current study (e.g., for network realism, run additional sensitivity analyses with small-world network structures).
3. **Boundary Conditions Table:**Create a table that explicitly delineates organizational characteristics where model findings are likely valid vs. invalid.

PART 10: RESEARCH IMPLEMENTATION

The research has high potential for implementation in several domains.:

1. Policy Development: Findings can inform the design of fraud prevention policies that account for organizational scale effects. Regulatory bodies (e.g., financial authorities,

audit standards bodies) can develop risk-based guidance that differentiates expectations based on organizational size (Carter, 2020).

2. Organizational Design: The results show that structural properties (network connectivity, susceptibility to social influence) shape fraud dynamics. This suggests that organizations can redesign control environments by focusing on structural interventions rather than just individual-level training.Carter, 2020).

3. Fraud Detection Systems: Model insights can inform the development of advanced anomaly detection systems that account for non-linear scaling. Machine learning-based detection systems can be calibrated differently for organizations of different sizes (Carter, 2020).

4. Academic Research: This study opens a new research direction integrating complexity science with criminology. Findings validate the utility of agent-based modeling in fraud research.

Implementation Challenges

1. Practitioner Skepticism: Because findings are based on simulation, practitioners may require additional empirical validation before investing in new prevention strategies (Carter, 2020).

2. Complexity Barriers: Organizational adoption of a complexity science perspective on fraud management requires a significant paradigm shift from current individual-focused approaches.

3. Context Dependency: Findings may vary across industries, geographies, and organizational types, requiring contextual adaptation.

Improvement Suggestions

1. **Implementation Roadmap:**Develop detailed roadmap for how organizations of different sizes can implement findings:
    - For organizations growing from 500 to 1,000 employees: strengthen network-based controls, invest in integration systems
    - For large multinational corporations: develop modular control architectures that can scale with growth
2. **Pilot Studies:**Propose collaborative pilot studies with willing partner organizations to test whether scaling relationships predict real fraud risk patterns
3. **Training Programs:**Develop training materials for fraud professionals and governance practitioners on the complexity science perspective on fraud management.
4. **Development Tools:**Create interactive tools (eg, web-based calculators) that allow practitioners to input organizational characteristics and receive fraud risk assessments based on the model's scaling relationships
