# Supplementary material for: Organisational scale and fraud volatility: An exploratory agent-based simulation of occupational fraud dynamics
Source: PLoS One. 2026 Jul 17;21(7):e0342964. doi: 10.1371/journal.pone.0342964 (PMC13378982; doi:10.1371/journal.pone.0342964)
Supplement: Attachment — Submitted filename: ROUND 3_14 Mei PONE-D-25-65420_R3.pdf [file pone.0342964.s008.pdf]

MANUSCRIPT REVIEW: PONE-D-25-65420_R3

1. **Title Review**

Current title: "Scaling of occupational fraud with organizational size: Evidence from an exploratory agent-based model."

Weakness

- The phrase "Scaling of occupational fraud with organizational size" less explicitly emphasizes that the findings are conceptual and simulation-based, not empirical estimates based on field data.
- The title does not fully reflect another key dimension that is very prominent in the manuscript, namely the volatility of fraud and the role of social network structures.

Improvement Suggestions

- Consider summarizing the title into a more focused two-clause form, for example (just an example):
  - "Organizational Scale and Occupational Fraud Dynamics: An Exploratory Agent-Based Simulation"
  - or "Non-linear Scaling of Occupational Fraud with Organizational Size: An Agent-Based Simulation Study".
- Add an explicit emphasis that this study is exploratory and simulation-based to avoid readers' expectations of empirical calibration or formal hypothesis testing.
- If the journal emphasizes practical contributions, it may be worth considering including the terms "fraud risk" or "fraud volatility" in the title to make the implications for risk management more immediately apparent.

2. Abstract Review

The abstract includes the background, methods (9,000 simulations, organizational size 25–6,400 agents), main findings (superlinear scales for average fraud and volatility, and practical implications), and the study's position as an exploratory model.

Weakness

- Abstracts tend to be long and dense with information, potentially exceeding the usual word limit and reducing readability for the general reader of the journal.
- The sentence structure in the abstract is quite complex, with some sentences containing many clauses so that the reader's cognitive load increases.
- The statement regarding the exploratory nature and uncalibration of the model to empirical data is already there, but it is embedded in the middle of the paragraph, so that it can be missed by readers who read quickly.
- Policy and practice implications are mentioned in general terms, but there is no clear distinction between theoretical contributions and practical implications.

Improvement Suggestions

- Summarize the abstract into 3–4 very short paragraphs (or 1 structured paragraph) with the following pattern:
    a. Background and gaps;
    b. Methods (ABM, organizational size range, key parameters);
    c. The main quantitative findings (scale exponent values and their interpretation) and qualitative findings (the role of scale and networks);
    d. Main theoretical and practical implications.
- Consider stating explicitly, near the beginning of the abstract, that this study is an "exploratory, conceptual agent-based simulation not empirically calibrated to real-world data."
- Simplify long compound sentences into shorter sentences with a clear subject–verb–object–adverb structure to improve clarity for readers across disciplines.

- Clarify the distinction between theoretical contributions (e.g., incorporating organizational scale and volatility into the framework of fraud as a systemic phenomenon) and practical consequences (e.g., the need for non-linearly increasing control strengthening in large organizations).

3. Preliminary Review

The introduction outlines the context of fraud in England and Wales, the limitations of empirical data, calls for an interdisciplinary approach, and points to the need to view fraud as a systemic and dynamic phenomenon.

Weakness

- The introduction is quite long and contains a lot of descriptive statistics and general information that could be summarized without compromising the argument.
- The transition from the general picture of national fraud to the specific issue of "scaling with organizational size" could still be sharpened, so that the relationship between survey figures and questions of organizational size is more explicit.
- The formulation of the research agenda (research questions) only appears after several pages, so the reader has to wait a long time to understand the main focus of the study.
- Specific contributions to the literature (e.g., to economic criminology, the fraud triangle, complexity theory, and the criminological ABM literature) have been mentioned, but have not been summarized concisely at the end of the introduction.

Improvement Suggestions

- Summarize the general context sections (national fraud statistics, psychological impact) into one or two paragraphs that directly address the main empirical issue,

namely the ambiguity of the relationship between organizational size and fraud prevalence.

- Clarify and accelerate the transition to the main gap:
  - that survey studies and ACFE reports provide inconsistent findings regarding the relationship between organizational scale and fraud,
  - and that detection limitations make it difficult to use observational data to confidently test scaling patterns.
- Move the research questions statement to an earlier part of the introduction, for example after the first 2–3 paragraphs, so that the reader immediately has a map of the direction of the study.
- Add an introductory concluding paragraph that explicitly presents: (1) the gap; (2) the approach; (3) the main contribution in 3–4 concise sentences, with clear declarative language.

4. Review of Theoretical Studies and Hypotheses.

The manuscript includes a very extensive literature review, including: organizational fraud survey data, the fraud triangle literature and its variants, critiques of the individualistic approach, fraud as a systemic phenomenon, complexity theory and scaling laws, and ABM literature in criminology. The authors also state that this study does not test formal hypotheses, but rather begins with research questions and theoretical mechanisms.

Weakness

- The literature review is very comprehensive but tends to be descriptive and extensive, with many sub-arguments repeated in the discussion section, leading to redundancy.
- The review structure is not yet fully organized thematically with a clear outline (e.g.: (1) empirical evidence on fraud and organizational size; (2) individual-level fraud theories and their limitations; (3) complexity theory and scaling; (4) ABM in economic criminology).

- Although the authors chose not to formulate formal hypotheses, conceptual expectations (e.g., the superlinear vs. linear scaling conjecture, or the implications of denser networks) have not been formulated explicitly as “propositions” that can guide the interpretation of the results.
- There is a risk of “overloading” the reader with philosophical references and discussions (critical realism, Bhaskar, Bunge) before the reader understands the core mechanisms of the model.

Improvement Suggestions

- Reorganize the literature review thematically with sharper subheadings, for example:
  - “Fraud against Organizations and Organizational Scale”;
  - “Individualistic Accounts: Fraud Triangle and Its Critiques”;
  - “Fraud as a Systemic and Complex Phenomenon”;
  - "Agent-Based Modeling in Criminology and Economic Crime".
- After the primary literature review, explicitly formulate 2–4 theoretical propositions or “expectations” (although not called formal hypotheses), for example:
  - That the average level of fraud will increase superlinearly with the size of the organization;
  - That fraud volatility is increasing faster than average;
  - That changes in network connectivity and social influence will moderate the scale–fraud relationship. This formulation will help the reader assess the extent to which the simulation results align or contradict expectations.
- Consider moving some of the philosophical discussion of critical realism to the discussion section so that the introduction and literature review remain focused on establishing a more immediate empirical and theoretical context for the model.

5. Research Methods (Agent-Based Modeling and Analysis)

The methodology section explains the use of NetLogo 6.4.0, agent specifications (motive, opportunity, attitude), social network, q and α parameters.$_{k}$, organizational size, number of simulations (9,000), and the analysis approach using log–log regression for estimating the scaling exponent.

Weakness

1. **ABM model specifications and reporting standards**

   - Despite the detailed narrative explanation, the manuscript does not explicitly mention or follow a standard reporting framework such as the ODD (Overview, Design concepts, Details) protocol common for ABM publications in top-tier journals.
   - Some technical aspects (e.g., how the random network is formed, the random seed initialization, and the agent update scheme) are described, but could be more systematic in a table or sub-heading format to facilitate replication.

2. **Justification of binary parameters and assumptions**

   - The choice of a 0.5 Bernoulli distribution for the three attributes (motive, opportunity, attitude) has been justified as a neutral baseline, but the explanation is still qualitative and could be deepened with empirical references or more explicit sensitivity simulations.
   - Binary representations for complex variables such as “pro-fraud attitude” and “opportunity” are recognized as simplifications, but there is no additional exploration of whether the scaling results are robust to variations in initial probabilities or more realistic distribution schemes (e.g., asymmetric distributions).

3. **Statistical analysis of simulation output**

   - The log–log regression approach to simulation results is quite common for estimating scaling exponents, but discussion of the structure of the simulation data (inter-run dependencies, pseudo-replication, or clustering based on parameter configuration) is still limited.

- The removal of 14 zero fraud peak values from the log–log regression is mentioned, but there is no sensitivity analysis of whether alternative approaches (e.g., adding a small constant) change the exponent meaningfully.

4. **Verification and validation**

   - The verification and validation section states that the model is not validated against empirical data and emphasizes “plausibility” alone, but does not describe systematic robustness testing procedures (e.g., global sensitivity analysis of key parameters, or additional experiments that show the limits of the model's behavior).

Improvement Suggestions

- Consider organizing the methods section according to the ODD framework or an equivalent framework recognized in the ABM community, so that the model description is easier to review and replicate.
- Add a table or appendix summarizing all parameters (range, tested values, justification, and theoretical implications) beyond the existing Table 1. This table should separate structural, behavioral, and output parameters.
- Expand the explanation regarding sensitivity analysis:
  - Explain whether the authors have tested alternative variations of the initial probability (e.g., 0.3 or 0.7) and whether the scaling pattern remains stable;
  - If you haven't already, consider adding a short experiment or at least an explicit discussion of the possible impact of the variation on the results.
- Clarify the structure of the simulation data and justify the use of standard OLS regression on log–log output: whether each point in the regression represents an average over 20 runs per configuration, or a run-by-run observation; and how the authors address issues of independence and clustering.
- In the verification/validation section, describe the steps of exploring the model's behavior (e.g., checking face validity, testing extreme parameters, or checking

whether the output returns to the expected pattern at certain parameters), with short examples so that the reader has more confidence in the model's robustness.

6. Data Analysis / Results

The results section describes the log–log relationship between organizational size and three outcomes: mean fraud, peak fraud, and fraud volatility, as well as the interaction effects with q_avg and $\alpha._k$.

Weakness

- The presentation of the results focuses heavily on scaling exponents and p-values, but provides little intuitive illustration in the form of concrete numerical examples (e.g., examples of small vs. large organizations with average fraud rates and volatility).
- Readers who are not experts in scaling laws may have difficulty connecting the abstract exponents with practical implications, even though the author has included the interpretation “doubling size ≈ 2.37 times fraud”.
- There is no direct visualization of the time series dynamics of fraud for some representative scenarios, so the term “volatility” remains abstract for readers unfamiliar with the simulation output.

Improvement Suggestions

- Add a short subsection providing a simple numerical example: for example, an illustration of how the average fraud rate and volatility differ for N = 100 vs. N = 3,200, based on representative simulation results, so that readers understand the effect size in more concrete units.
- If technically feasible, consider displaying time series (change in the number of fraud agents per tick) for two or three contrasting configurations (e.g. small vs large organizations with low and high q_avg) to visualize the differences in volatility patterns that are one of the key claims.

- Clarify in the text whether the reported exponent numbers are from regressions on the inter-run average per configuration or across individual runs, and explain the implications of this approach for the interpretation of R^2 and p-values.

7. Discussion and Discussion of Results

The discussion is very rich, connecting the results to complexity theory, critical realism, fraud dynamics, the fraud triangle, and Fraud Field Theory, as well as implications for the perception of fraud as a systemic phenomenon.

Weakness

- The discussion section is very long and contains some repetition of arguments that have already appeared in the literature review (e.g., criticism of the fraud triangle, the emphasis that fraud is systemic, and the discussion of the Empirical–Actual–Real domains).
- The flow of the discussion sometimes switches quickly between high philosophical aspects and practical implications, without always providing clear structural markers for the reader.
- The study's main focus—namely the non-linear relationship between organizational scale, fraud rates, and volatility—is sometimes overshadowed by extensive theoretical discussion.
- The direct relationship between the findings and the three research questions formulated at the beginning can be made more explicit and structured.

Improvement Suggestions

- Consider restructuring the discussion into subsections with informative headings, for example:
    a. “Revisiting the Research Questions”;
    b. “Fraud as a System-Level Phenomenon”;

c. “Implications for Fraud Theory (Fraud Triangle and Beyond)”;
d. “Implications for Practice and Policy”;
e. "Positioning within Critical Realism and Complexity Theory".

- In the first subsection, explicitly answer each research question by referring directly to the main results, before expanding the discussion to theoretical and practical consequences.
- Reduce the repetition of narratives regarding criticism of the fraud triangle and the position of critical realism by referring to the previous section, so that the discussion appears sharper and more economical.
- Explicitly state what the core theoretical contributions are (e.g., including organizational scale and volatility as explicit dimensions of fraud dynamics; demonstrating that the assumption of per capita linearity is often misleading) and how these contributions complement, rather than replace, individual frameworks such as the fraud triangle.

8. Conclusion

The conclusion section summarizes that fraud is better understood as an emergent property of organizational systems that changes with scale, and highlights the value of a complexity-based simulation approach to the study of fraud.

Weakness

- Conclusions tend to repeat arguments that have been discussed at length in the discussion, making them feel less concise and a bit repetitive.
- The conclusion structure does not clearly separate: (1) a summary of the empirical results of the model; (2) theoretical contributions; (3) practical implications; and (4) a further research agenda.
- Some sentences still use a long narrative style with several clauses, which can be simplified.

Improvement Suggestions

- Summarize the conclusion into 3–4 paragraphs that each focus on:
    a. Short answers to research questions related to scaling fraud with organizational size;
    b. Major theoretical contributions to the fraud, complexity, and economic criminology literature;
    c. Key practical implications for control design and governance;
    d. The most priority direction for further research.
- Avoid repeating methodological details and critical arguments presented in previous sections; focus on the most important take-home messages.
- Use sentences with a shorter, clearer structure (subject–predicate–object–adverb) to improve readability, especially for readers from other disciplines.

9. Research Limitations and Further Research Agenda

This section is quite honest and comprehensive: the authors acknowledge the limitations of empirical calibration, the simplicity of the network structure, the binary attribute scheme, the limitations of a closed simulation environment, and the challenges of external validation.

Weakness

- While rich, the exposition of limitations is so long and detailed that some key points are drowned out by more technical details.
- Some aspects of the limitations (e.g. random network representation vs. hierarchical organizational structures) reappear in other sections, giving rise to repetition.
- The further research agenda has not been clearly prioritized; many possible extensions are mentioned, but not arranged from the most pressing/realistic to the more speculative.

Improvement Suggestions

- Group the limitations into 3–4 main categories, for example:

    - (1) Organizational structure and network;
    - (2) Representation of agent behavior;
    - (3) Empirical calibration and validation;
    - (4) Scope of fraud (internal only, does not include external perpetrators).
- In each category, summarize 1–2 sentences explaining the limitations followed by 1 sentence indicating the most immediate direction for further research.
- Highlight the 2–3 most important further research priorities (e.g., empirical integration of network structures; calibration against specific organizational data; use of the model as a test-bed for policy interventions), and place these priorities at the end of the section as concrete guidance for future researchers.
