# Supplementary material for: Organisational scale and fraud volatility: An exploratory agent-based simulation of occupational fraud dynamics
Source: PLoS One. 2026 Jul 17;21(7):e0342964. doi: 10.1371/journal.pone.0342964 (PMC13378982; doi:10.1371/journal.pone.0342964)
Supplement: Attachment — Submitted filename: 2026-03-17 Response to reviewers PLOS One-1.docx [file pone.0342964.s005.docx]

## Responses to Reviewer 1:

| **Reviewer reference** | **Recommendations** | **Comments** |
| --- | --- | --- |
| 1.1 | No empirical validation against real organizational fraud data, been acknowledged but not adequately addressed. Please revise. | The revised manuscript now addresses this issue more explicitly in the Limitations and Directions for Future Research section. The paper clarifies that the present study is intended as a theoretical and exploratory simulation examining the possibility that fraud dynamics scale with organisational size, rather than as a calibrated model of any specific organisation.  Further references to empirical studies have been added, alongside recognition of the epistemic limitations of these.  The discussion also notes that empirical validation in this domain is inherently challenging because organisational fraud data are typically partial and incomplete, reflecting only detected or reported incidents. The revised text therefore emphasises the need for future work to explore opportunities for empirical calibration and validation using organisational data where available, including potential collaboration with organisations that can provide access to internal fraud or audit datasets. |
| 1.2 | Visualization: Please add a conceptual framework diagram (research model) at the end of the Introduction or the beginning of the Methods to make it easier for readers to understand the relationship between variables. Do not forget to include adequate explanations upon the model. | We thank the reviewer for this helpful suggestion. In the revised manuscript, the conceptual relationships between organisational size, interaction structures, behavioural susceptibility and emergent fraud outcomes are now explained more explicitly in the Introduction and Methodology sections. These revisions clarify how the key elements of the model relate to one another within the simulation framework. While a formal conceptual diagram has not been added, the structure of the model and the relationships between variables are now described more clearly in the text. |
| 1.3 | Parameter Justification - q_avg values (0.15 vs 0.95) lack empirical grounding; no sensitivity analysis. Please provide the references as well as the sensitivity analysis | We thank the reviewer for this helpful suggestion. The revised manuscript clarifies that the social influence parameter (q_avg) represents the susceptibility of agents to adopt the attitudes of those in their interaction network, drawing conceptually on the literature on behavioural contagion and social influence in organisations. Rather than relying on two illustrative regimes, the experimental design has now been substantially expanded. The model was re-run across a wider parameter space with more granular values for both q_avg and the connectivity scaling parameter (αₖ), the addition of a smaller organisational size (N = 25), and twenty repetitions for each parameter configuration. This produced a dataset of 9,000 simulation runs and allowed the sensitivity of fraud outcomes to varying levels of social influence to be examined more systematically. These revisions are described in the Methodology and Findings sections. |
| 1.4 | No sensitivity analysis or alternative model assumptions tested. Please conduct the testing, and provide us the results and interpretation | We thank the reviewer for this helpful suggestion. In response, the simulation experiment has been substantially expanded and re-run across a broader parameter space. The revised design includes more granular values for the key behavioural parameters (q_avg and αₖ), the addition of an additional organisational size (N = 25), and twenty repetitions for each parameter configuration. This resulted in a dataset of 9,000 simulation runs and allows the sensitivity of fraud outcomes to different parameter combinations to be examined more systematically. The revised Methodology and Findings sections now report these analyses and discuss how the results vary across the expanded parameter space. |

## Responses to Reviewer 2:

| **Reviewer reference** | **Recommendations** | **Comments** |
| --- | --- | --- |
| 2.1 | The introduction should include brief empirical evidence (audit reports or case  studies) demonstrating how large-scale fraud can occur due to system failures, to strengthen the  justification for a systemic approach. Also, acknowledging existing research in the field of  organizational culture and internal control would provide a more balanced background. | We thank the reviewer for this helpful suggestion. The introduction has been revised to include additional empirical context illustrating how organisational and system-level conditions can contribute to large-scale fraud events, drawing on evidence from fraud surveys and official reporting. The revised text also places greater emphasis on the role of organisational culture and social influence processes in shaping fraud risk, helping to situate the systemic perspective developed in the paper alongside existing research on behavioural norms within organisations. |
| 2.10 | Present complete  tables/regressions including standard errors for the scaling exponents in each regime and band.  This way, readers know how certain the “superlinear” statement is – e.g., β=1.2 with p<0.05, etc.  – and so on. | In the revised manuscript, the results tables have been expanded to report the estimated coefficients together with their standard errors and statistical significance. This allows the scaling relationships identified in the analysis to be interpreted more transparently and provides readers with a clearer indication of the precision of the estimated scaling exponents. |
| 2.11 | Add visualizations – log-log graphs of output vs. N for several key regimes.  Visualizations will clearly show where the curve bends or changes slope. | We thank the reviewer for this helpful suggestion. In the revised manuscript, additional visualisations have been incorporated to illustrate the relationship between organisational size and fraud outcomes. In particular, log–log plots are now included to show how fraud measures vary across the full range of simulated organisational sizes. These figures help make the scaling relationships identified in the regression analysis more transparent and allow readers to see how fraud outcomes change as organisational size increases. |
| 2.12 | Use change-point  detection methods instead of manually splitting the groups. There are statistical techniques to  detect non-linearity (piecewise regression, spline). This is more objective than assuming three  static groups. | We thank the reviewer for this helpful suggestion. In the revised manuscript, the earlier analysis based on manually defined organisational size groups has been removed following the expansion and re-analysis of the simulation experiment. Organisational size is now treated as a continuous variable and the results are estimated using regression models across the full range of simulated organisational sizes. This approach allows the scaling relationships between organisational size and fraud outcomes to be assessed directly without imposing predefined group boundaries. As a result, the analysis no longer relies on manually specified size bands, addressing these concerns about arbitrary thresholds. |
| 2.13 | Analyze internal mechanisms: for example, calculate the time autocorrelation of  fraud rates to see if there is a periodic cycle; examine the distribution of the duration of high fraud  episodes; or examine the profiles of several extreme runs to identify path-dependent scenarios  (perhaps there are runs where fraud is extinct vs. runs where fraud is dominant, even if  parameters are the same). This in-depth analysis will provide an understanding of the “why”  behind the “what” emerging from the data. | The aim of the present study was to examine whether organisational fraud dynamics exhibit scaling relationships with organisational size, rather than to undertake a detailed time-series analysis of internal simulation trajectories. For this reason, additional analyses such as autocorrelation of fraud rates, episode duration distributions, or path-dependent case comparisons were not incorporated into the current paper. However, the revised manuscript now highlights these types of analyses as promising directions for future research in the Limitations and Directions for Future Research section, noting that the simulation framework developed here provides a basis for exploring such dynamics in greater detail. |
| 2.14 | Provide a “Limitations” paragraph. Explicitly state the model’s  limitations and implications: e.g., “Our findings are hypothetical, simulation-based. While the  emerging patterns are consistent with complexity theory, the model’s limitations (e.g., factor X  has not been modeled) mean caution is needed before generalizing to all organizations.” This  demonstrates the author’s critical scientific approach to his or her own work. | The revised manuscript now includes an expanded Limitations and Directions for Future Research section prior to the conclusion. This section explicitly acknowledges that the findings are derived from a simulation-based model and therefore represent stylised explorations of organisational fraud dynamics rather than direct empirical estimates. It also discusses key modelling limitations, including the simplified representation of organisational structures, the use of fixed behavioural parameters, and the exclusion of external organisational or environmental factors. These clarifications emphasise that the results should be interpreted as exploratory insights into possible system dynamics rather than as universally generalisable predictions. |
| 2.15 | Offer ways to  implement the results. For example, “Practitioners can apply these findings by conducting  organizational network analysis (identifying key employees who connect units) and ensuring  ethics programs focus on these nodes to prevent the normalization of misconduct.” Such  suggestions will make the discussion more concrete. | The revised manuscript expands the Implications for Policy and Practice section to include more concrete examples of how the findings could be operationalised in organisational settings. In particular, the discussion now highlights the potential value of approaches such as organisational network analysis to identify highly connected individuals who may shape behavioural norms across teams, allowing ethics and integrity programmes to focus attention on key nodes within organisational networks where behavioural influence may be most significant. |
| 2.16 | Compare with conventional insights.  Explain how this new view complements existing knowledge. For example, “These results do not  mean we should abandon individual screening (the fraud triangle), but they do suggest that  systemic factors (networks and culture) create the conditions in which the fraud triangle can  manifest itself extensively. Therefore, effective prevention needs to target both levels: the  individual and the system.” This balance prevents skeptical readers from dismissing the new idea  entirely. | The revised manuscript now clarifies more explicitly how the complexity-oriented perspective developed in the paper complements rather than replaces existing individual-level explanations of fraud. In particular, the discussion emphasises that frameworks such as the fraud triangle remain useful for explaining individual decisions to offend, but that systemic factors—such as organisational scale, network structures and social influence—shape the conditions under which those individual motivations translate into widespread organisational fraud. This clarification has been incorporated into the Discussion section to highlight the complementary relationship between individual and system-level perspectives. |
| 2.17 | Review the literature through the lens of the results. Are these findings consistent with  Kim & Xiao's (they found model-data alignment in welfare programs)? Do they support Button's  argument (fraud field equilibrium)? A discussion of the literature will further clarify the study's  contribution. | The revised Discussion now engages more directly with the existing simulation and fraud literature through the lens of the present findings. In particular, the manuscript explains how the results are consistent with Kim and Xiao’s broader conclusion that simulation can reproduce patterns comparable to real-world fraud systems, while also showing that organisational scale may itself operate as a generative mechanism. The discussion also now addresses Button et al.’s Fraud Field Theory more explicitly, noting that while it represents an important advance, the present findings challenge the idea that fraud systems tend toward steady-state equilibrium, instead suggesting that outcomes may vary non-linearly as scale, connectivity and susceptibility interact. |
| 2.18 | Write a Limitations section before the conclusion. This is academically important. Explicitly mention the limitations of the model (which we discussed in the methods). Examples include the assumption of static variables, the absence of external factors, etc., as well as data limitations (hidden fraud). By outlining these in the conclusion, the reader is assured that the authors have not overlooked the study's shortcomings. | We thank the reviewer for this helpful suggestion. In the revised manuscript, a dedicated Limitations and Directions for Future Research section has been added prior to the conclusion. This section discusses the simplifying assumptions of the model, including the use of static behavioural parameters, the absence of external organisational or environmental shocks, and the reliance on stylised representations of organisational interaction structures. It also acknowledges the broader empirical challenges associated with fraud research, including the hidden nature of fraud and resulting data limitations. This addition clarifies the scope of the study while identifying areas for further development of the modelling approach. |
| 2.19 | Prioritize Further Empirical Validation. The authors have recommended mirroring real networks and comparing data.[38]This suggestion is very appropriate. Additionally, they could recommend collaborating with companies to test-bedding this model. For example, applying the model to internal data from a large company (with adjusted parameters) to see if the model's predictions match the areas of vulnerability identified. This would bridge the model-reality gap | The revised manuscript now expands the Limitations and Directions for Future Research section to emphasise the importance of further empirical validation. In particular, the discussion highlights the potential value of calibrating and testing the model using organisational data, including collaboration with organisations to explore whether the model can help identify areas of vulnerability within real systems. This addition underscores the importance of bridging the gap between simulation-based insights and empirical organisational contexts. |
| 2.2 | Broaden  the scope of the literature – include empirical findings on the relationship between organizational  size and fraud incidents (e.g., global fraud survey reports often report that larger companies suffer  greater absolute fraud losses, but perhaps smaller percentages), and research on organizational  culture and internal control as influences on fraud. | We thank the reviewer for providing this helpful suggestion. The literature review has been expanded to include additional empirical evidence on the relationship between organisational size and fraud, including findings from global fraud surveys such as those published by the Association of Certified Fraud Examiners and related empirical sources. The revised manuscript also incorporates further discussion of research on organisational culture and internal control as factors influencing fraud risk. These additions help situate the modelling approach within the broader empirical literature on fraud in organisational settings. |
| 2.20 | Don't Forget the Humans: While this research is about systems, ultimately, policies must be implemented by people. Recommendations could address the importance of an effective integrity culture program. The model results clearly show that a highly contagious culture (attitude) can have detrimental effects. Therefore, strengthening the integrity culture of large companies through training, rotating leaders to ensure the company's values are shared, etc., is a practical recommendation aligned with the findings (reducing effective q_avg) | The revised manuscript expands the Implications for Policy and Practice section to address the human and organisational dimensions of fraud prevention more explicitly. Additional literature has been incorporated on the propagation of pro- and antisocial behaviour in organisational settings, highlighting that social influence operates as a two-way process: interaction networks can spread both ethical norms and misconduct. The discussion therefore emphasises that integrity programmes, leadership signalling and the management of organisational influence networks may play an important role in reducing the diffusion of pro-fraud norms within organisations. |
| 2.21 | Alignment with Regulations: Organizations are often subject to anti-fraud regulations (e.g., mandatory internal audits for public companies). The authors could recommend that regulators address the scale factor: for example, requiring mid-sized companies to have adequate audit/investigation functions due to their vulnerability. This public policy recommendation would demonstrate the broad impact of the findings. | We thank the reviewer for this helpful suggestion. In the revised manuscript, the discussion of policy implications has been expanded to consider how organisational scale may have implications for regulatory expectations around governance, audit and counter-fraud capacity. In addition, the previous categorisation of organisations into small, medium and large has been removed following substantial model re-runs using a wider range of organisational sizes. This allows the findings to be presented in terms of generalisable scaling relationships rather than discrete organisational categories. The revised discussion appears in the Implications for Policy and Practice section. |
| 2.3 | Link the complexity framework to other  theories, such as Contagion theory (the theory of the transmission of deviant behaviour in  organizations) or percolation theory in social networks, to demonstrate a broader scientific basis  for the use of ABM. | We thank the reviewer for this suggestion. The theoretical discussion has been expanded to situate the complexity framework more clearly alongside related perspectives, including research on behavioural contagion in organisations and network-based diffusion processes. This additional discussion helps clarify how agent-based modelling provides a useful method for examining the transmission of behavioural norms through organisational interaction networks, thereby linking the study more explicitly to a broader body of work on social influence and collective behaviour. |
| 2.4 | State the limitations of previous approaches as well as the challenges of  new approaches fairly. For example, the author could acknowledge: “The fraud triangle paradigm  is simple and lacks systems considerations, but even the complexity approach faces validation  challenges | This comment has been addressed through the addition of a more comprehensive limitations section and explicit acknowledgement of the problems of validation of models of this nature. |
| 2.5 | Non-binary & Dynamic Variables: Include fraud severity or loss amounts, so that  the output is not only a count of people but also financial impact. Allow the agent's opportunity or  motive to vary over time (e.g., through a periodic random distribution or influenced by external  factors such as company performance). - Real Organizational Structure: Use a more realistic  network topology, such as a small-world network with departmental clusters, or real company  communication network data (if available) to mirror the structure. The authors themselves  recommend this as a further research | We thank the reviewer for these constructive suggestions. The present study deliberately employs a simplified representation of organisational systems in order to isolate and examine the scaling dynamics of fraud emergence. Variables such as fraud severity or financial loss, dynamic changes in motive or opportunity, and more realistic organisational network structures were therefore not incorporated into the current model. However, the revised manuscript now highlights these extensions more explicitly in the Limitations and Directions for Future Research section, noting that incorporating richer behavioural dynamics, financial loss measures and empirically grounded network structures (e.g., clustered or small-world organisational networks) would represent valuable directions for future work. |
| 2.6 | Detection/Punishment Mechanism: Add a rule that a fraud  agent has a chance of being detected every tick, then being removed (fired) or reset to 0 (if the  punishment interpretation is deterrent). This will create new dynamics (new members replacing  those fired, etc.) and see if the system continues to produce similar patterns with control  interventions. - | We thank the reviewer for this thoughtful suggestion. The present model intentionally focuses on examining the possibility that fraud dynamics scale with organisational size, and therefore concentrates on the structural mechanisms relevant to scaling rather than incorporating organisational control interventions such as detection or punishment mechanisms. For this reason, such mechanisms were not added to the current model. The revised manuscript now clarifies this design choice in the Limitations and Directions for Future Research section and identifies the incorporation of control dynamics as a promising direction for future work. The paper also signposts related modelling research examining fraud control mechanisms (Freeman, 2026), which explores how different configurations of preventive and detective controls influence fraud dynamics within simulated organisational systems. |
| 2.7 | Richer Parameter Experiment: Perform sensitivity analysis with various q_avg  values (instead of 0.15 and 0.95, try 0.3; 0.5; 0.7) to map how the transition from stable to unstable  depends on size. Also, try different initial conditions (e.g., initially 1% fraud) to see if the initial  percentage only affects the time to steady state or changes the long-term fraud level. | We thank the reviewer for this helpful suggestion. In the revised manuscript, the parameter space for susceptibility to social influence (q_avg) has been expanded to include a broader range of values, allowing the model to explore how fraud dynamics vary across differing levels of social influence. The baseline fraud propensity itself was not varied; however, additional justification for the chosen initial parameterisation has been added to the Methodology section. This approach follows the modelling logic used by Davis and Pesch (2013), who likewise held the underlying fraud propensity constant in order to focus on how interaction dynamics within the organisational system shape emergent fraud behaviour. |
| 2.8 | Number  of Simulations & Statistics: Increase the number of replications or use statistical approaches (e.g.,  ANOVA) to assess the influence of each factor (size, q, αₖ) and their interactions. Since the model  is full-factorial, it should be possible to analyze it with a 2×3×8 ANOVA or non-linear regression.  The author only performed regressions per regime, whereas an integrated analysis could have  demonstrated the significance of interaction effects (e.g., the effect of size depends on αₖ and q). | We thank the reviewer for this helpful suggestion. In response, the model was re-run with an expanded experimental design, including twenty repetitions per parameter configuration, the addition of a smaller organisational size (N = 25), and more granular parameter ranges for both the social influence parameter (q_avg) and the connectivity scaling parameter (αₖ). This produced a substantially larger simulation dataset (9,000 runs, approx. 20 hours to run) and allowed integrated regression models to be estimated including organisational size, q_avg, αₖ and their interaction terms, enabling the moderating effects of these parameters on fraud outcomes to be examined directly. These revisions are reflected in the Methodology and Findings sections. |
| 2.9 | Qualitative Empirical Validation: Although it is difficult to obtain complete fraud data, the model  can be validated qualitatively. For example, compare the simulated pattern with the pattern  reported by internal auditors: do medium-sized companies sometimes experience more severe  fraud spikes than larger companies? Finding real-life case studies (e.g., scandals in medium-  sized vs. large companies) can be a check on the consistency of the model results with reality. | The revised manuscript now expands the discussion of empirical context to consider how the model’s patterns can be compared qualitatively with available evidence on organisational fraud. In particular, the paper now draws on findings from sources such as the Association of Certified Fraud Examiners and UK Home Office reporting to illustrate how observed fraud patterns across organisations of different sizes can provide a useful point of comparison for the simulated dynamics. While acknowledging that such datasets capture only detected or reported incidents, this discussion helps situate the model results within the broader empirical landscape of organisational fraud. |

## Responses to Reviewer 3:

| **Reviewer reference** | **Recommendations** | **Comments** |
| --- | --- | --- |
| 3.1 | Conceptual Framework and Theoretical Gap The manuscript would benefit from a sharper articulation of the theoretical gap. The authors are encouraged to explicitly state: Which dominant or mainstream approaches to organisational fraud detection are being challenged (e.g., linear risk models, regression-based approaches). What novel insights complexity theory and scaling laws offer as an alternative analytical lens. The discussion of Complexity Theory and Scaling Laws should be more explicitly linked to organisational fraud dynamics, rather than remaining largely implicit. In particular, the discussion related to Fraud Field Theory requires further clarification. The manuscript should explain more clearly why and how the present findings undermine the conventional assumptions of this theory, especially in the Discussion section, including the underlying logic of reasoning. | The revised manuscript now articulates the theoretical gap more clearly in both the Introduction and Discussion sections. The paper now explains more explicitly that much existing research and policy practice in organisational fraud detection is grounded in analytical approaches that implicitly assume linear or proportional relationships between organisational characteristics and fraud outcomes, such as regression-based risk models or case-level explanatory frameworks. The revised text clarifies that these approaches are valuable for identifying correlates of fraud but are less well suited to analysing how fraud may emerge from complex organisational interaction structures.  The discussion of complexity theory and scaling laws has therefore been expanded to explain how these perspectives provide an alternative analytical lens. In particular, the manuscript now emphasises how interaction networks, behavioural contagion and organisational scale can produce emergent dynamics that are not easily captured by linear analytical frameworks. The results are interpreted in this context, showing how organisational scale may act as a generative condition shaping the diffusion of fraudulent behaviour within organisational systems.  The relationship with Fraud Field Theory has also been clarified. While the theory represents an important advance in recognising the systemic nature of fraud risk, the revised discussion explains that the present findings suggest that fraud systems may not naturally stabilise around equilibrium conditions. Instead, the interaction of organisational size, connectivity and behavioural susceptibility can produce shifting regimes of fraud activity. This clarification helps position the study as extending existing fraud theory by exploring how organisational scale and interaction dynamics may shape the conditions under which fraud emerges and propagates. |
| 3.2 | Operational Definitions and Variables The manuscript currently lacks a table of operational definitions. A dedicated table defining key variables, parameters, and agent attributes used in the model is strongly recommended. This would significantly enhance transparency and replicability, particularly for readers less familiar with agent-based modelling. | We thank the reviewer for this suggestion. To improve the clarity of the model specification, the revised manuscript now includes a table in the Methodology section summarising the key agent attributes, model parameters and outcome variables together with their definitions and roles within the simulation. This addition is intended to make the structure of the model and the operationalisation of its components more transparent for readers. |
| 3.3 | Methodology: Population, Sample, and Experimental Design The method section should be presented more systematically, preferably in tabular form, to improve clarity. The description of simulations ranging “from 50 to 6,400 employees across six experimental regimes” requires further elaboration, particularly: How these organisational sizes were determined. Whether they are theoretically motivated, empirically informed, or arbitrarily selected. How the six experimental regimes differ from one another in structural or behavioural assumptions. The manuscript refers to “population” and “sample” implicitly; however, in the context of ABM, these concepts should be explicitly clarified to avoid conceptual ambiguity. | We thank the reviewer for this helpful suggestion. The Methodology section has been substantially revised to improve clarity and structure. In particular, the model variables and parameters are now presented in tabular form to provide a clearer overview of the experimental design. The description of organisational sizes has also been expanded to explain the rationale for their selection, which follow a logarithmic progression to enable the analysis of scaling relationships across multiple orders of magnitude rather than representing empirically derived organisational categories. In addition, the manuscript now clarifies that in an agent-based modelling context each simulation represents a complete artificial population rather than a statistical sample, with repeated runs used to explore stochastic variability in model outcomes. |
| 3.4 | The repeated use of terms such as “larger” and “smaller” organisations would benefit from clearer operational and conceptual definitions, especially given the analytical modelling approach. Explicit thresholds or criteria for categorising organisational size (small, medium, large) should be stated and justified. | We thank the reviewer for this helpful suggestion. In the revised manuscript, the earlier categorisation of organisations into small, medium and large groups has been removed following the expansion and re-analysis of the simulation experiment. Organisational size is now treated as a continuous variable within the scaling analysis rather than as discrete categories. The text has been revised accordingly to clarify that organisational size refers to the number of agents in the simulated organisation. The literature review has also been expanded to discuss how empirical studies often apply differing and sometimes conflicting thresholds when defining small, medium and large organisations, which further supports the decision to analyse organisational size as a continuous variable rather than relying on predefined size classes. |
| 3.5 | Results Interpretation and Discussion The findings related to linear, sublinear, superlinear, and volatile phases are intriguing; however: The manuscript should provide a clearer explanation of why these patterns emerge, particularly in the Discussion section. The rationale behind why such patterns are not detectable through global regressions but are revealed via banded analysis should be more explicitly articulated. The argument that medium-sized organisations face the highest fraud risk, as observed in regimes R2, R3, R5, and R6, is important but requires: Stronger theoretical justification. Additional support from prior empirical studies or closely related literature to strengthen the claim. | In the revised manuscript the analysis has been substantially restructured. The earlier presentation based on discrete organisational size bands has been removed following a re-run of the model across an expanded parameter space. The results are now presented in terms of general scaling relationships between organisational size and fraud outcomes, estimated using integrated regression models that incorporate social influence (q_avg), connectivity scaling (αₖ), and their interaction effects. The discussion has also been revised to provide a clearer explanation of the mechanisms generating different scaling behaviours within the model and to clarify why such dynamics may not be visible when heterogeneous regimes are aggregated in simple global regressions. |
| 3.6 | Limitations Section In the Limitations section, the statement beginning with “Like all agent-based models, this study relies on a simplified representation of an organisational system…” does not require citations. This is a widely accepted and self-evident methodological limitation in ABM research. Removing unnecessary citations would improve conciseness and focus. | In light of this suggestion that citation has now been removed. |
| 3.7 | Practical and Policy Implications The implications section would benefit from stronger empirical and theoretical grounding, particularly for claims such as: Organisations with dense networks, high susceptibility to influence, and rapid growth being inherently prone to disproportionate fraud escalation. Where possible, these claims should be supported by: Recent empirical studies, or Explicit linkage to established theories in organisational behaviour, criminology, or complexity science. | Thank you for this suggestion. The article has been substantially re-written in light of the comments of the five reviewers. Through this, these suggestions have been addressed, including a more comprehensive policy and practice section. |
| 3.8 | References and Literature Update The reference list should be updated to reflect more recent literature, ideally within the last 5 years, or a maximum of 10 years, except for clearly established grand theories. Several cited works are relatively dated and should either be: Justified as foundational (e.g., Bunge, Bettencourt et al.), or Supplemented with more recent studies that demonstrate the contemporary relevance of the theoretical framework. | We thank the reviewer for this helpful suggestion. The reference list has been updated to incorporate additional recent scholarship, particularly from the past decade, relating to organisational fraud, computational modelling and complexity-informed approaches to crime analysis. Where earlier works are retained, these have been positioned more clearly as foundational contributions to the theoretical framework underpinning the study. This revision helps ensure the manuscript reflects both the contemporary literature and the key theoretical foundations of the field. |

## Responses to Reviewer 4:

| **Reviewer reference** | **Recommendations** | **Comments** |
| --- | --- | --- |
| 4.1 | I appreciate the relevant sources from the literature review, but I encourage you to include more recent sources. | The literature review has been updated to incorporate additional recent scholarship on fraud, organisational misconduct and computational approaches to crime analysis. These additions help ensure the review reflects the current state of research while also situating the study within the growing body of work examining crime dynamics in complex organisational systems. |
| 4.2 | Also, I appreciate your research idea, of using simulations, and I confess that these results could be useful for further research that could use them in artificial intelligence practice. For this reason, your results should be more robustness and be generalized. For this reason, I appreciate to better describe the independent variables, their distribution, and how they could interact in order to support the Table 1 results (for these table, please give more references and explain how do you support the scenarios. | We thank the reviewer for this helpful suggestion. In the revised manuscript, the description of the model variables and parameters has been expanded to provide greater clarity on the independent variables used in the simulation and how they interact within the model. A dedicated table has been added in the Methodology section summarising the key variables, parameters and their operational definitions. Additional explanation has also been included to clarify how the experimental scenarios are constructed and how the behavioural parameters interact to produce the outcomes reported in Table 1. Where appropriate, further references have been added to situate the modelling assumptions within the relevant literature. |
| 4.3 | The results are interesting but give more practical applications and connect the results with practice. | In light of this comment a far more comprehensive implications for policy and practice section has been added. This explains how the results may be of use in future counter fraud strategies. |

## Responses to Reviewer 5:

| **Reviewer reference** | **Recommendations** | **Comments** |
| --- | --- | --- |
| 5.1 | Please add the literature on organizational fraud and corporate crime. Then emphasize the novelty of the research on the first scaling-based ABM of organizational fraud. Link it to the context of contemporary organizations (large complex organizations). | We thank the reviewer for this helpful suggestion. The literature review has been expanded to incorporate additional research on organisational fraud and corporate crime, including empirical evidence from sources such as the Association of Certified Fraud Examiners and the UK Economic Crime Survey. These additions help situate the study more firmly within the existing literature while also highlighting the continuing empirical uncertainty surrounding the relationship between organisational scale and fraud. The revised text also emphasises the contribution of the present study as an agent-based modelling analysis of fraud scaling within organisational systems, linking the research more explicitly to the structural characteristics of contemporary large and complex organisations. |
| 5.2 | The reviewer suggests adding the criminal/organizational ABM literature. Please summarize the section on the history of the fraud triangle. Emphasize the position of the ABM in criminology and computational social science. | We thank the reviewer for this helpful suggestion. In the revised manuscript, the literature review has been expanded to situate the study more clearly within the broader literature on agent-based modelling in criminology and computational social science. In doing so, the section discussing the historical development of the fraud triangle has been shortened and refocused, allowing greater emphasis on how agent-based modelling can be used to formalise and test criminological mechanisms within simulated social systems. This revision positions the present study more explicitly within the emerging use of computational approaches to examine crime dynamics. |
| 5.3 | Add literature on organizational/firm scaling. Explain the concept of phase transition more formally. Define fraud scaling explicitly. | The literature review has been expanded to incorporate additional research on organisational and firm-level scaling in complex systems. The manuscript now also clarifies the concept of fraud scaling, explaining how it refers to the relationship between organisational size and fraud outcomes when analysed using log–log scaling relationships. In addition, the discussion of phase transitions has been clarified in the context of the model results, explaining how changes in interaction structures and behavioural parameters can produce shifts in fraud dynamics within the simulated organisational system. These clarifications have been incorporated into the Literature Review, Methodology, and Discussion sections. |
| 5.4 | Explain the basis for band selection. Add sensitivity band thresholds. Use formal breakpoint analysis or piecewise regression. | In the revised manuscript, the earlier presentation based on discrete organisational size bands has been removed following a substantial expansion and re-analysis of the simulation experiment. The model was re-run across a broader parameter space and the results are now presented using integrated regression models that examine the relationships between organisational size, social influence (q_avg), connectivity scaling (αₖ), and their interaction effects. This approach avoids the need for manually defined size bands and instead allows the scaling dynamics to be assessed directly across the full range of simulated organisational sizes. |
| 5.5 | Add simulation confidence intervals. Add a summary figure regime map. Avoid strong causal claims without empirical validation. | We thank the reviewer for these helpful suggestions. In the revised manuscript, additional summary statistics are now reported from the expanded simulation dataset, which includes 9,000 runs across the parameter space, allowing the variability of outcomes across repetitions to be examined more clearly. The presentation of the results has also been revised to include clearer visual summaries of how fraud outcomes vary across organisational size and behavioural parameters. In addition, the wording of the findings and discussion has been moderated to avoid overly strong causal interpretations, emphasising that the results represent simulation-based insights into possible system dynamics rather than empirically validated causal relationships. |
| 5.6 | Summarize the philosophical section. Add concrete practical implications. Add limits to the simulation's interpretation. | The philosophical discussion has been streamlined in the revised manuscript to focus more directly on its relevance to the modelling approach and the interpretation of the results. At the same time, the Discussion and Implications for Policy and Practice sections have been expanded to provide more concrete practical implications for organisational governance, integrity programmes and fraud risk management. In addition, the Limitations and Directions for Future Research section has been strengthened to clarify the interpretive boundaries of the simulation and the extent to which the findings should be understood as exploratory insights into possible system dynamics rather than direct empirical predictions. |
| 5.7 | Add limitations regarding external validity, structural realism, behavioural realism, and parameter uncertainty. | The Limitations and Directions for Future Research section has been expanded to discuss the boundaries of the modelling approach more explicitly. In particular, the revised manuscript now addresses issues of external validity, the simplified structural representation of organisations within the model, the behavioural assumptions embedded in the agent rules, and the uncertainty associated with parameter selection. These additions clarify the interpretive limits of the simulation while outlining avenues for future work to develop more empirically grounded model structures and parameters. |
| 5.8 | Clarify the methodological contribution. Clarify the contribution to fraud theory. Add an empirical research roadmap. | We thank the reviewer for this helpful suggestion. The revised manuscript now clarifies the study’s contributions more explicitly in the Discussion and Conclusion sections. In particular, the paper now distinguishes more clearly between its methodological contribution—demonstrating how agent-based modelling can be used to explore scaling dynamics in organisational fraud—and its theoretical contribution to the study of fraud as an emergent property of organisational systems. The conclusion has also been expanded to outline a possible empirical research agenda, including the potential for future work to calibrate and test simulation models using organisational data and to examine how fraud dynamics vary across different organisational contexts. |
