# Supplementary material for: Organisational scale and fraud volatility: An exploratory agent-based simulation of occupational fraud dynamics
Source: PLoS One. 2026 Jul 17;21(7):e0342964. doi: 10.1371/journal.pone.0342964 (PMC13378982; doi:10.1371/journal.pone.0342964)
Supplement: Attachment — Submitted filename: Reviewer 5_Comments.docx [file pone.0342964.s003.docx]

Dear Editor

PLOS ONE,

I would like to inform you that I have completed my review of the revised manuscript. Based on my evaluation, the authors have satisfactorily addressed the reviewer’s comments and revised the manuscript adequately. In my opinion, the revised manuscript is now acceptable, subject to the Editor’s final decision.

Thank you for the opportunity to review this manuscript.

Sincerely,
Reviewer
Prof. Tarjo

Responses to Reviewer 5:

| **Reviewer reference** | **Recommendations** | **Comments** | **Recommendation for the author's revision** |
| --- | --- | --- | --- |
| 5.1 | Please add the literature on organizational fraud and corporate crime. Then emphasize the novelty of the research on the first scaling-based ABM of organizational fraud. Link it to the context of contemporary organizations (large complex organizations). | We thank the reviewer for this helpful suggestion. The literature review has been expanded to incorporate additional research on organisational fraud and corporate crime, including empirical evidence from sources such as the Association of Certified Fraud Examiners and the UK Economic Crime Survey.  These additions help situate the study more firmly within the existing literature while also highlighting the continuing empirical uncertainty surrounding the relationship between organisational scale and fraud. The revised text also emphasises the contribution of the present study as an agent-based modelling analysis of fraud scaling within organisational systems, linking the research more explicitly to the structural characteristics of contemporary large and  complex organisations. | The reviewer's comments have been adequately addressed. Revisions to this section are acceptable. |
| 5.2 | The reviewer suggests adding the criminal/organizational ABM literature. Please summarize the section on the history of the fraud triangle. Emphasize the position of the ABM in criminology and computational social science. | We thank the reviewer for this helpful suggestion. In the revised manuscript, the literature review has been expanded to situate the study more clearly within the broader literature on agent-based modelling in criminology and computational social science. In doing so, the section discussing the historical development of the fraud triangle has been shortened and refocused, allowing greater emphasis on how agent-based modelling can be used to formalise and test criminological mechanisms within simulated social systems. This revision positions the present study more explicitly within the emerging use of computational  approaches to examine crime dynamics. | The reviewer's comments have been addressed appropriately. The revisions to this section are adequate and acceptable. |
| 5.3 | Add literature on organizational/firm scaling. Explain the concept of phase transition more formally. Define fraud scaling explicitly. | The literature review has been expanded to incorporate additional research on organisational and firm-level scaling in complex systems. The manuscript now also clarifies the concept of fraud scaling, explaining how it refers to the relationship between organisational size and fraud outcomes when analysed using log–log scaling relationships. In addition, the discussion of phase transitions has been clarified in the context of the model results, explaining how changes in interaction structures and behavioural parameters can produce shifts in fraud dynamics within the simulated organisational system. These clarifications have been incorporated into the Literature Review, Methodology, and  Discussion sections. | This section has been addressed appropriately. The author's response is deemed adequate and satisfactory. |
| 5.4 | Explain the basis for band selection. Add sensitivity band thresholds. Use formal breakpoint analysis or piecewise regression. | In the revised manuscript, the earlier presentation based on discrete organisational size bands has been removed following a substantial expansion and re-analysis of the simulation experiment. The model was  re-run across a broader parameter space and the results are now presented using integrated regression models that examine the relationships between organisational size, social influence (q_avg), connectivity scaling (αₖ), and their interaction effects. This approach avoids the need for manually defined size bands and instead allows the scaling dynamics to be assessed directly across  the full range of simulated organisational sizes. | The reviewer's comments have been addressed appropriately. The revisions are deemed adequate and acceptable. |
| 5.5 | Add simulation confidence intervals.  Add a summary figure regime map. Avoid strong causal claims without empirical validation. | We thank the reviewer for these helpful suggestions. In the revised manuscript, additional summary statistics are now reported from the expanded simulation dataset, which includes 9,000 runs across the parameter space, allowing the variability of outcomes across repetitions to be examined more clearly. The presentation of the results has also been revised to include clearer visual summaries of how fraud outcomes vary across organisational size and behavioural parameters. In addition, the wording of the findings and discussion has been moderated to avoid overly strong causal interpretations, emphasising that the results represent  simulation-based insights into possible system dynamics rather than empirically  validated causal relationships. | This section has been addressed appropriately. The author's response to the reviewer's comments is deemed satisfactory. |
| 5.6 | Summarize the philosophical section. Add concrete practical implications. Add limits to the simulation's interpretation. | The philosophical discussion has been streamlined in the revised manuscript to focus more directly on its relevance to the modelling approach and the interpretation of the results. At the same time, the Discussion and Implications for Policy and Practice sections have been expanded to provide more concrete practical implications for organisational governance, integrity programmes and fraud risk management. In addition, the Limitations and Directions for Future Research section has been strengthened to clarify the interpretive boundaries of the simulation and the extent to which the findings should be understood as exploratory insights into possible system dynamics rather than direct empirical  predictions. | The author has made appropriate and adequate revisions. This section can be considered satisfactory. |
| 5.7 | Add limitations regarding external validity, structural realism, behavioural realism, and parameter uncertainty. | The Limitations and Directions for Future Research section has been expanded to discuss the boundaries of the modelling approach more explicitly. In particular, the revised manuscript now addresses issues of external validity, the simplified structural representation of organisations within the model, the behavioural assumptions embedded in the agent rules, and the uncertainty associated with parameter selection. These additions clarify the interpretive limits of the simulation while outlining avenues for future work to develop more empirically grounded model structures and  parameters. | The reviewer's comments have been addressed appropriately. The revisions are deemed adequate and acceptable. |
| 5.8 | Clarify the methodological contribution. Clarify the contribution to fraud theory. Add an empirical research roadmap. | We thank the reviewer for this helpful suggestion. The revised manuscript now clarifies the study’s contributions more explicitly in the Discussion and Conclusion sections. In particular, the paper now distinguishes more clearly between its methodological contribution— demonstrating how agent-based modelling can be used to explore scaling dynamics in organisational fraud—and its theoretical contribution to the study of fraud as an emergent property of  organisational systems. The conclusion has also been expanded to outline a possible empirical research agenda, including the potential for future work to calibrate and test simulation models using organisational data and to examine how fraud dynamics vary across different  organisational contexts. | This section has been addressed appropriately. The substance of the reviewer's comments has been adequately and satisfactorily addressed. |
