# Supplementary material for: Organisational scale and fraud volatility: An exploratory agent-based simulation of occupational fraud dynamics
Source: PLoS One. 2026 Jul 17;21(7):e0342964. doi: 10.1371/journal.pone.0342964 (PMC13378982; doi:10.1371/journal.pone.0342964)
Supplement: Attachment — Submitted filename: 2026-04-12 Response to reviewers PLOS One Second Round.docx [file pone.0342964.s007.docx]

## Responses to Reviewer 1:

| **Reviewer reference** | **Recommendations** | **Comments** |
| --- | --- | --- |
| 1 | The author clearly stated that the research was exploratory, meaning no actual world data available for comparison,  In my view, despite these deficiencies, the manuscript offers enrichment of the fraud knowledge.  It might be happed or it might be never happened, who knows | Thank you for taking the time to review the manuscript, and for your recognition of the value that it offers. As you have noted, the challenges of real-world comparisons make this a wicked problem. |

## Responses to Reviewer 2:

| **Reviewer reference** | **Recommendations** | **Comments** |
| --- | --- | --- |
| 2.1 | The author should modify the title to explicitly indicate the exploratory and simulated nature, for example by adding the words "simulated" or "exploratory modelling". The authors should more explicitly relate the term "occupational fraud" to the model structure (for example, explaining that the model represents embezzlement or certain internal misappropriation at the level of trusted individuals). | Thank you for this helpful suggestion. The title has been revised to clarify the exploratory and simulation-based nature of the study. It now reads: “Scaling of occupational fraud with organisational size: Evidence from an exploratory agent-based model.”  In addition, the manuscript has been amended to more explicitly define how “occupational fraud” is represented within the model (line 380). A clarifying statement has been added to the methodology section specifying that the model captures employee-level misconduct undertaken against the organisation for personal benefit, corresponding to forms of internal misappropriation (e.g., expense fraud or abuse of organisational resources). This distinction also makes clear that the model does not represent corporate crime or external fraud. |
| 2.2 | The abstract should include an explicit sentence marking the study as an exploratory simulation based on a conceptual model, not an empirically-calibrated model. The abstract should provide a brief, more intuitive explanation of the main technical terms (for example, "the average fraud grows faster than the growth in the number of employees" as a translation of "superlinearly"). | Thank you for this suggestion. The abstract has been revised to more clearly indicate the nature of the modelling approach. An explicit sentence has been added clarifying that the model is exploratory and conceptual and not calibrated to empirical organisational data (line 410).  In addition, brief intuitive explanations have been incorporated alongside key technical terms describing the scaling relationships. In doing so, care has been taken to ensure that these explanations accurately reflect the cross-sectional nature of the analysis. Specifically, “superlinear” scaling is now clarified as indicating that larger organisations exhibit disproportionately higher levels of fraud relative to their size, rather than implying growth within a single organisation over time. These revisions are intended to improve accessibility while maintaining conceptual precision. |
| 2.3 | The introduction should be condensed by eliminating repetition and moving the more general theoretical discussion to the literature review section.  The author should add an explicit paragraph detailing the key contributions of the article in bullet points (for example, three main contributions to theory and method). | Thank you for this suggestion. The introduction has been reviewed and minor revisions have been made to reduce repetition and improve concision, particularly where similar points were reiterated regarding the epistemic limitations of fraud data and the need for system-level approaches.  At the same time, the overall structure of the introduction has been retained to preserve a clear framing of the research problem and its theoretical orientation at the outset of the paper. The more detailed theoretical development continues in the literature review section. |
| 2.4 | The authors should focus the literature review on sources that are truly relevant to the model design (scaling crime studies, contagion deviance, ABM fraud, and empirical studies on organisational size). In-depth philosophical or historical discussions about the fraud triangle can be summarised or partially moved to the conceptual discussion section. | Thank you for this helpful suggestion. On balance (and taking into account the fact this was not requested by other reviewers), the discussion of the fraud triangle literature has been retained, as it provides the conceptual foundation for the decision structure used within the model. |
| 2.5 | The authors should include a conceptual framework diagram that connects organisational size, network structure, social influence, and fraud outcomes (mean,  peak, volatility). The authors should formulate a set of explicit hypotheses, such as the expectation of superlinearity under certain conditions of connectivity and  contagion. | Thank you for this helpful suggestion. A conceptual framework diagram (Fig 1)has been added to visually summarise the relationships between organisational size, network structure, social influence, and fraud outcomes. This is now presented at the end of the literature review to provide a clear bridge between the theoretical framing and the model specification.  With respect to hypotheses, the study adopts an exploratory, simulation-based approach aimed at examining the behaviour of hypothesised generative mechanisms rather than testing formally specified hypotheses. To clarify the expected relationships, a set of analytical expectations has been introduced (line 89), outlining how organisational scale and behavioural parameters are anticipated to influence fraud dynamics. This approach is intended to retain conceptual clarity while remaining consistent with the study’s methodological design. |
| 2.6 | The authors should justify the selection of initial values and the distribution of agent attributes more clearly, or acknowledge it as a pure simplification and test the sensitivity to parameter variations (e.g., 0.2 – 0.8). In future research, the authors could consider continuous or multimodal representations for attitude and opportunity to approach real heterogeneity. | The manuscript has been revised to more clearly clarify the role of initial parameter values. The binary representations and initial probabilities used in the model are intended as simplifying assumptions that allow the interaction of core mechanisms to be examined, rather than as empirically calibrated estimates of real organisational populations. This clarification has been strengthened in the methodology section.  The study already examines a wide range of parameter regimes for key variables such as organisational size, network connectivity and susceptibility to social influence, which allows the robustness of observed patterns to be explored under varying structural and behavioural conditions.  The suggestion to incorporate continuous or multimodal representations of agent attributes is well taken and has been included as a direction for future research in the limitations section (line 931). |
| 2.7 | The authors should test at least one or two types of alternative network structures that are more realistic (e.g., small-world with department clusters and some long ties) to examine the robustness of the results.  The author should add an explanation of why the size range of 25–6,400 was chosen and how this range relates to the size of real organisations (e.g., business units or departments). | Thank you for these helpful suggestions.  With respect to network structure, incorporating more realistic organisational forms such as small-world or clustered networks would be a valuable extension. The current model uses randomly generated networks as a neutral baseline in order to isolate the effects of organisational scale and connectivity without introducing additional structural assumptions. The potential impact of alternative network structures has now been acknowledged in the limitations section as an important direction for future research.  The manuscript has also been revised to more clearly explain the rationale for the organisational size range used in the model. The range of 25–6,400 agents is intended to approximate variation across organisational sub-units and medium-to-large organisations, capturing differences across multiple orders of magnitude while remaining analytically tractable. |
| 2.8 | The authors should add basic diagnostic checks (residual plots, additional goodness-of-fit) to justify the use of the log-log linear model and the interpretation  of the power law. The authors should consider compact visualisations (heatmaps of exponent scaling as a function of q and αk) to replace lengthy narrative descriptions. | The manuscript has been revised to more explicitly report diagnostic checks for the log–log regression models. Additional text has been included in the methodology section (line 497) noting that residual plots were examined and did not indicate systematic departures from linearity or other violations that would undermine the use of the log–log specification.  With respect to visualisation, the current presentation focuses on clarity of interpretation through log–log plots and regression tables. The suggestion to use more compact visualisations such as heatmaps is well taken and has been noted as a direction for future work, where such approaches could provide a more concise representation of how scaling behaviour varies across parameter regimes. |
| 2.9 | The authors should add a subsection "Model verification and validation" that explains the steps of code verification, parameter sensitivity testing, and pattern matching with basic empirical facts (for example, the prevalence of fraud never being zero or 100 percent persistently).  The authors should relate the simulation output to some empirical patterns (e.g. the distribution of fraud losses in large vs. small companies) to provide an initial indication that the model operates within a reasonable behavioural domain. | A subsection titled “Model verification and validation” has been added to the methodology section. This clarifies how the model is assessed within the study’s exploratory, generative framework.  The manuscript now makes explicit that the model is not intended to reproduce or predict empirical datasets, and therefore formal validation against real-world data is not undertaken. Instead, model behaviour is assessed through systematic exploration of parameter regimes varying organisational size, network connectivity scaling (αₖ), and susceptibility to social influence (q_avg), allowing the consistency of observed patterns to be examined across a range of structural and behavioural conditions.  The section also clarifies that evaluation is framed in terms of plausibility rather than empirical fit, with simulation outputs interpreted in relation to broad empirical regularities discussed in the literature. This is intended to demonstrate that the model operates within a behaviourally plausible domain while remaining consistent with the study’s methodological approach. |
| 2.10 | The authors should include more verbal interpretations linking the β values to practical consequences for the organization (for example, describing how the risk  of fraud changes when the organization doubles in size under certain regimes).  The authors should clean up all the result tables, ensure label consistency, and remove any remnants of formatting from word processing. | Thank you for this helpful suggestion. The manuscript has been revised to include clearer verbal interpretations of the estimated scaling coefficients. In particular, the findings section now provides illustrative examples of how fraud outcomes change with organisational size, including the implications of differences between organisations of varying size under the estimated scaling relationships. These additions are intended to make the practical significance of the results more accessible while remaining consistent with the cross-sectional nature of the analysis. |
| 2.11 | The discussion should balance criticism of the individualistic view of fraud with a critical reflection on the simplifications and limitations of the models used.  The author should condense the repetitive philosophical discussions and replace them with a more concrete discussion on how organisations can utilise insights  regarding scale effects and informal networks. | Thank you for this helpful suggestion. The discussion has been revised to provide a clearer balance between the critique of individualistic approaches to fraud and reflection on the limitations of the modelling approach. Additional text has been included to explicitly acknowledge the simplifying assumptions underpinning the model and the partial nature of its representation of organisational dynamics.  The manuscript has also been revised to strengthen the practical orientation of the discussion, with clearer links drawn between the theoretical framing, the simulation results, and their implications for organisational risk management. Minor reductions have been made to repetitive conceptual discussion to improve concision, while retaining the core theoretical argument. |
| 2.12 | The author should link practical recommendations to specific model scenarios, for example, highlighting parameter regimes that produce exceptionally high volatility and explaining relevant organisational actions.  The authors should explicitly label the practical implications as "preliminary thoughts" that require confirmation from real organisational data before widespread adoption. | Thank you for this helpful suggestion. The manuscript has been revised to more explicitly link the practical implications to the types of conditions represented in the model. Additional text has been added to relate differences in connectivity and susceptibility to social influence to variations in volatility and system stability, and to outline how these may inform different organisational interventions.  The implications have also been further qualified to emphasise their indicative and exploratory nature. The manuscript now makes explicit that these findings require further empirical investigation before they can be applied in specific organisational contexts. |
| 2.13 | The authors should align the entire manuscript with a strong emphasis on the exploratory and tentative nature of the results, especially in the conclusion and implications. The author should discuss in more detail how significant differences in real-world organisational structures (e.g., multi-divisional, global) can limit the direct application of the model's results. | The manuscript has been revised to more consistently emphasise the exploratory and tentative nature of the findings, particularly in the conclusion and implications sections. Additional text has been added to clarify that the results should be interpreted as indicative of plausible system-level dynamics rather than as predictive estimates of fraud behaviour in real organisational settings.  The limitations section has also been expanded to more explicitly discuss how differences between the modelled organisational structures and real-world organisations—particularly multi-divisional, geographically dispersed, and functionally differentiated structures—may limit the direct applicability of the findings. These revisions are intended to provide clearer guidance on the scope and interpretation of the results. |
| 2.14 | The author should substantially shorten the manuscript (15–20%) with a focus on eliminating repetition and moving highly philosophical conceptual discussions to an appendix or a separate article. The author should conduct a thorough final technical edit to correct all tables, captions, and formatting traces before the next submission. | Thank you for this suggestion. The manuscript has been reviewed and minor revisions made to improve concision, including tightening of phrasing in several sections.  However, the overall structure and level of conceptual discussion have been retained, as these are central to the paper’s contribution in linking complexity theory, agent-based modelling, and the study of organisational fraud. The aim has been to improve clarity and readability while preserving the coherence and integrity of the theoretical argument.  On the formatting point, this may be artifacts from any conversion to other formats, as the tables etc. appear to be correct in the revised manuscript previously submitted, and this point has not been picked up by other reviewers. |

## Responses to Reviewer 3:

| **Reviewer reference** | **Recommendations** | **Comments** |
| --- | --- | --- |
| 3.1 | While the model clearly specifies the generative mechanism underlying fraud behaviour, the assumption of equal initial probabilities for motive, opportunity, and pro-fraud attitude may limit generalisability to real-world organisations. | Thank you for this helpful observation. The manuscript has been revised to clarify the role of the initial parameterisation. The use of equal initial probabilities for motive, opportunity, and pro-fraud attitude is intended as a neutral baseline that allows the interaction of core mechanisms to be examined, rather than as a representation of real organisational distributions.  This assumption is now more explicitly acknowledged as a simplifying abstraction in the methodology and its implications for generalisability are noted in the limitations section. |
| 3.2 | the parameterisation of social influence (q_avg) and network connectivity scaling (αₖ) is scenario-based and not empirically calibrated, which should be emphasised as a limitation in the discussion. | Thank you for this helpful suggestion. The manuscript has been revised to more clearly emphasise that the parameterisation of social influence (q_avg) and network connectivity scaling (αₖ) is scenario-based and not empirically calibrated. This point is now explicitly stated in the limitations section, clarifying that the results should be interpreted as illustrating how different structural and behavioural conditions may shape fraud dynamics, rather than as empirically calibrated estimates of real-world effects. |
| 3.3 | Third, peak fraud regressions excluded fourteen runs with zero values, which could introduce minor bias in scaling estimates; this exclusion should be discussed explicitly. | The manuscript has been revised to explicitly discuss the exclusion of zero values from the log-transformed peak fraud regressions. A clarification has been added to the limitations section noting that, while this exclusion is necessary because the logarithm of zero is undefined, it may introduce a minor upward bias in the estimated scaling relationship for peak fraud. |
| 3.4 | Finally, although the study convincingly demonstrates system-level dynamics, the practical implications for organisational risk management and counter-fraud strategies could be expanded, particularly in linking observed scaling behaviours to actionable interventions. | Thank you for this helpful suggestion. The “Implications for policy and practice” section has been revised to more explicitly link the observed scaling behaviours to actionable interventions. In particular, the manuscript now distinguishes between implications for resourcing, governance, and intervention design, clarifying how superlinear scaling and increased volatility may require non-proportional resourcing, greater alignment of oversight with organisational complexity, and more targeted, system-aware control strategies. These revisions are intended to make the practical significance of the findings clearer and more directly applicable to organisational risk management. |

## Responses to Reviewer 4:

| **Reviewer reference** | **Recommendations** | **Comments** |
| --- | --- | --- |
| 4 | Thank you for the opportunity to read and review your research paper. I appreciate again the research idea, the strong theoretical background, the research methodology and the manner you presented and discussed the research results. I appreciate also the manner that you have adequately addressed reviewers' comments raised in a previous round of review and you feel that this manuscript is now acceptable for publication. The manuscript technically sound, and the data support the conclusions. The statistical analysis been performed appropriately and rigorously. You made all data underlying the findings in their manuscript fully available. The manuscript is presented in an intelligible fashion and written in standard English. | I appreciate the time that you have taken to review the article again in such depth. Many thanks for undertaking this and for your kind and thoughtful comments. |

## Responses to Reviewer 5:

| **Reviewer reference** | **Recommendations** | **Comments** |
| --- | --- | --- |
| 5 | I would like to inform you that I have completed my review of the revised manuscript. Based on my evaluation, the authors have satisfactorily addressed the reviewer’s comments and revised the manuscript adequately. In my opinion, the revised manuscript is now acceptable, subject to the Editor’s final decision.  Thank you for the opportunity to review this manuscript. | Thank you for taking the time to review the revised manuscript. I really appreciate your support and guidance. |
