# Supplementary material for: Organisational scale and fraud volatility: An exploratory agent-based simulation of occupational fraud dynamics
Source: PLoS One. 2026 Jul 17;21(7):e0342964. doi: 10.1371/journal.pone.0342964 (PMC13378982; doi:10.1371/journal.pone.0342964)
Supplement: Attachment — Submitted filename: 2026-05-11 Response to reviewers PLOS One.docx [file pone.0342964.s009.docx]

## Responses to Reviewer 2:

| **Reviewer reference** | **Recommendations** | **Comments** |
| --- | --- | --- |
| 2.1 | Model Validation and Calibration (CRITICAL PRIORITY) Finding: The model operates in an empirical vacuum with parameter choices that are not calibrated against real organisational data.  Required Actions: - Conduct a systematic literature review of fraud surveys to extract empirical ranges for parameters such as fraud prevalence rates, susceptibility to influence, and network connectivity ratios. - Develop sensitivity analyses that comprehensively explore the parameter space and identify which scenarios produce outcomes consistent with empirical fraud data. - Create a validation matrix that compares simulated fraud patterns with patterns in empirical datasets (e.g., BIS fraud surveys and Kroll fraud reports). - Minimum Acceptable Outcome: Demonstrate that at least 70-80% of simulated scenarios produce fraud dynamics within empirically observable ranges (Carter, 2020) | The reviewer's concerns regarding calibration are acknowledged, but we respectfully contend that the requests outlined go beyond what is appropriate for an explicitly exploratory simulation study of this kind, and in several respects unfortunately mischaracterise what the paper already contains.  The exploratory and conceptual nature of the model is stated clearly and repeatedly throughout the manuscript. This framing reflects a deliberate methodological choice grounded in the paper's theoretical basis. As the paper discusses at length, empirical fraud datasets capture only detected and reported incidents, representing a partial and systematically biased view of underlying fraud activity. Because of this, calibrating model parameters against such data would therefore not constitute rigorous validation. Instead it would reproduce the detection biases that simulation is specifically used to circumvent.  The paper already contains a systematic exploration of the parameter space across 9,000 simulations spanning wide ranges of organisational size, social influence susceptibility, and network connectivity scaling. This constitutes the sensitivity analysis the reviewer requests.  The existing model validation subsection explicitly frames evaluation in terms of plausibility against broad empirical regularities rather than predictive fit, consistent with established practice for exploratory agent-based models in criminology and adjacent fields.  We also note that the specific requests would fundamentally alter the nature and scope of the study. We do not consider it appropriate at this stage, and after two prior rounds of review, to undertake revisions of this magnitude, which would effectively require the paper to be rewritten as a different study entirely.  Finally, we were unable to locate a source identified as “Carter, 2020" that We would welcome clarification of this reference.  In the absence of a verifiable methodological standard, we do not consider this 70%80% threshold to represent a binding requirement, and we respectfully maintain that the plausibility-based evaluation approach adopted in this paper is consistent with established practice for exploratory agent-based models in criminology and adjacent social science fields. |
| 2.2 | Organisational Realism (High Priority) Finding: The model uses random networks that do not reflect real hierarchical, departmental, and power structures in organisations.  Required Actions: - Implement at least two alternative network structures: (a) hierarchical branching networks reflecting organisational charts and (b) small-world networks with community structure - Re-run the complete simulation suite with alternative network structures. - Compare findings to understand robustness in relation to network assumptions. - Discuss in the results how findings generalise or are specific to random network assumptions. (Carter, 2020) | This limitation is fully acknowledged in the manuscript. The limitations section explicitly notes that real organisations exhibit hierarchical reporting relationships and departmental structures not captured by the random network approach, and the future research section already identifies small-world and modular network structures as a priority for subsequent work.  Implementing two alternative network architectures and re-running the complete simulation suite would constitute a substantial new research programme rather than a moderate revision, and we do not consider this appropriate at the third round of review of an explicitly exploratory study.  In response to this comment, we have strengthened the future research section to explicitly include hierarchical branching structures and to acknowledge that testing robustness to alternative network assumptions is an important next step.  We again note that "Carter, 2020" has not been locatable in the relevant literature and would welcome clarification of this reference. |
| **2.3** | 3. Generalisability and Boundary Conditions (HIGH PRIORITY) Findings: Conclusions suggest general principles, but the boundaries of applicability are unclear.  Required Actions: - Create an explicit framework delineating (a) organisational types where findings are highly applicable, (b) organisational contexts requiring caution, and (c) organisational characteristics where predictions may fail. - Develop 2-3 empirical case studies or vignettes showing how findings apply in different real-world contexts - Discuss explicitly how findings generalise across industries, countries, organisational structures, levels of digitalisation | The paper already explicitly cautions against unrestricted generalisation, and the limitations section addresses multiple boundary conditions including structural realism, behavioural realism, parameter uncertainty, the restriction to internal fraud, and the closed simulation environment. In response to this comment, we have modestly expanded this section to more explicitly characterise the organisational contexts where findings are most and least applicable.  The development of empirical case studies or vignettes would constitute substantively new qualitative research outside the scope of a simulation study, and systematic discussion of generalisation across industries, countries, and digitalisation levels goes beyond what the present model can currently support. |
| 2.4 | . Actionability for Practitioners (MODERATE PRIORITY) Findings: Discussion provides theoretical insights but limited concrete guidance for fraud prevention practice.  Required Actions: - Develop a "Practitioner Implementation Guide" as supplementary material - Translate findings into specific, testable recommendations - Example: "Organisations expanding from 1,000 to 2,500 employees should anticipate fraud risk increasing by a factor of 2.0-2.4 and implement prevention infrastructure scaling at a minimum of 2.4x" - Create decision trees or matrices for risk managers to identify optimal prevention strategies given their organisational characteristics | The paper includes a dedicated policy implications section translating the simulation findings into guidance for counter-fraud practice at the organisational and systemic level. In response to this comment, we have modestly strengthened this section to make practical takeaways more concrete.  However, we respectfully caution against the kind of specific numerical recommendations illustrated in the reviewer's example. The model is explicitly exploratory and not calibrated against empirical organisational data; publishing precise scaling estimates as actionable thresholds would misrepresent the model's precision and risk misleading practitioners.  We would also note that the reviewer's example—anticipating fraud risk changes as an organisation expands from one size to another—reflects a longitudinal interpretation that the model does not support. The simulation compares organisations of different sizes cross-sectionally; it does not model the dynamic process of organisational growth. Framing findings in those terms would therefore misrepresent what the study actually does demonstrate.  The development of a practitioner implementation guide, decision trees, or risk matrices would constitute a separate practice-focused publication rather than a journal revision. |
| 2.5 | 1. Methodology Presentation Issues: - Model description scattered across the methodology section; lacks clear algorithm pseudocode or flowchart - Parameter values table incomplete; missing some parameter ranges and justifications Actions: - Create detailed model specification figure showing agent types, behaviours, decision rules - Provide complete parameter table with empirical sources, ranges explored, and sensitivity impact - Add pseudocode or UML diagram for key algorithms (social influence mechanism, fraud detection mechanism) | The methodology section has been refined across two prior rounds of review and already contains a conceptual framework figure (Fig 1), a comprehensive parameter table (Table 1) including ranges explored for all swept parameters, and detailed prose descriptions of the social influence mechanism and agent decision rules.  We would also note that several of the reviewer's concerns in this section appear to reflect a concern that model code and data are not publicly available. As noted in our responses to earlier comments, the full model code and simulation output data are archived on Zenodo (Freeman, 2026; https://doi.org/10.5281/zenodo.19056223), providing complete algorithmic detail beyond what pseudocode would offer. O  This is stated in both the methodology section and the data availability statement. We therefore respectfully disagree with the characterisation of the methodology as incomplete, and other reviewers have noted the availability of the data and code to aid replication. |
| 2.6 | Writing Clarity Issues: Some passages are long and have multiple embedded clauses, reducing their readability. - Inconsistent terminology (e.g., "fraud agents" vs. "potential fraudsters") Actions: - Revise for more concise sentence structure - Standardise terminology across paper - Break complex paragraphs into multiple shorter paragraphs | The manuscript has been reviewed for terminological consistency and sentence concision.  A number of complex paragraphs have been broken into shorter units to improve readability, and sentence structure has been reviewed for concision as part of the broader revision for length. |
| 2.7 | Data Visualisation Issues: - Log-log plots in results difficult to interpret without additional context. - Missing visualisations of fraud dynamics over time (time series plots) - No illustration of model architecture or interaction structures Actions: - Enhance figures with insets showing linear-scale relationships for comparison - Add time series plots showing fraud trajectory in different organisational sizes - Create conceptual figure showing model structure, agent types, and interaction mechanisms - Ensure all figures are high resolution, with clear labels and comprehensive captions Data Visualisation Issues: - Log-log plots in results are difficult to interpret without additional context - Missing visualisations of fraud dynamics over time (time series plots) - No illustration of model architecture or interaction structures Actions: - Enhance figures with insets showing linear-scale relationships for comparison - Add time series plots showing fraud trajectory in different organisational sizes - Create a conceptual figure showing model structure, agent types, and interaction mechanisms - Ensure all figures have high resolution, clear labels, and comprehensive captions Literature Update Issues: - Some references are outdated; missing recent papers in fraud detection, complex systems modelling Actions: - Update the literature review with papers from 2022-2025 in: organisational fraud, complex systems criminology, agent-based models in social science - Add discussions of recent work on scaling laws in criminal networks, organisational resilience | The log-log plots are the primary means through which the scaling relationships — the central finding of the paper — are presented. The relationship between organisational size and fraud outcomes is inherently multiplicative, and log-log space is the appropriate representation for power-law scaling.  Adding linear-scale insets would not aid interpretation and risks implying that a linear framing is equally appropriate. The text already explains the scaling relationships in both mathematical and plain-language terms.  The conceptual model architecture is already illustrated in Fig 1, and figure quality, labelling, and captioning have been reviewed in prior rounds, with the figures passed through the tool provided by PLOS One for this purpose.  The suggestion of time series plots is acknowledged as a potentially useful illustration of the volatility dynamics discussed in the paper. However, the archived simulation data captures summary statistics per run rather than tick-by-tick trajectories. Generating these figures would require additional simulation work beyond the scope of a revision at this stage.  Regarding the literature, the paper already contains citations up to 2025 and 2026, including recent work in organisational fraud, complexity-informed criminology, and agent-based modelling. The reviewer has not identified specific gaps or missing papers, and we therefore do not consider a wholesale literature update to be warranted. |
| 2.8 | Statistical Rigour 1. Confidence Intervals: Provide 95% confidence intervals for all estimated scaling exponents, not just point estimates. | We thank the reviewer for this comment. As a result, 95% confidence intervals have been added to Tables 2 and 3. The intervals are narrow throughout, consistent with the precision expected from 9,000 simulations, and confirm the stability of the estimated scaling exponents. |
| 2.9 | Model Fit Statistics: Report adjusted R², residual diagnostics, and goodness-of-fit tests for power-law relationships | Adjusted R² values are not reported separately as, given N = 9,000, adjusted R² is virtually identical to R² in all models. Adding it would duplicate existing information without adding interpretive value. R² values are already reported in both Tables 2 and 3.  Residual diagnostics are already reported in the methodology section, which notes that visual inspection of residual plots showed no systematic departures from linearity in log-log space.  Regarding goodness-of-fit tests for power-law relationships, these are designed to assess whether a variable follows a power-law distribution within a dynamic system, for example, testing whether fraud events accumulate according to a power-law as an organisation grows over time. However, as noted in our response to another reviewer comments above, this paper does not model organisational growth. Instead the analysis is cross-sectional, comparing organisations of different sizes. |
| 2.10 | Multiple Comparisons: If conducting multiple hypothesis tests, apply appropriate corrections (Bonferroni or false discovery rate). | Multiple comparisons corrections are designed for situations where the same hypothesis is tested repeatedly, such as post-hoc ANOVA comparisons, and are not standard practice for regression coefficients across conceptually distinct models. The three regressions in Table 2 test separate hypotheses concerning theoretically distinct outcome variables. Furthermore, the paper is primarily estimation-focused rather than testing-focused, and with p < .001 throughout, any correction applied would leave all conclusions unchanged. |
| 2.11 | Effect Size: Report effect sizes, not just p-values, for increased interpretability. | The scaling exponents reported throughout the paper are themselves effect size measures: they directly quantify the magnitude of the relationship between organisational size and fraud outcomes in a form that is both statistically precise and practically interpretable. The paper translates these into explicit practical terms; for example, that a doubling of organisational size is associated with a 2.37-fold increase in average fraud.  Generic effect size statistics would add no interpretive value beyond the scaling exponents and R² values already provided. |
| 2.12 | Code Availability: Commit to depositing model code on the Open Science Framework or GitHub with complete documentation. | The model code and simulation output data are already publicly archived in a Zenodo repository (Freeman, 2026; https://doi.org/10.5281/zenodo.19056223), as noted in both the Methodology section and the Data Availability statement. Zenodo is a CERN-backed open repository that is listed among the recommended data sharing platforms in PLOS ONE's data availability guidelines, and provides a persistent DOI suitable for long-term archiving and citation.  We therefore consider this requirement to be already satisfied. |
| 2.13 | Data Availability: If using any datasets, provide clear statements about availability, licensing, and access procedures. | This study does not use any external datasets. All data analysed in the paper were generated by the agent-based simulation conducted by the author. These simulation outputs are publicly available in the Zenodo repository (Freeman, 2026; https://doi.org/10.5281/zenodo.19056223), as stated in the Data Availability section of the manuscript. No third-party data licensing or access procedures are therefore applicable. |
| 2.14 | Random Seed: Report random seeds used for simulations for reproducibility. | We thank the reviewer for this comment. A brief clarification has been added to the Methodology section.  The simulations were run with rng_seed set to -1, meaning random seeds were not fixed and were not recorded.  This was a deliberate choice consistent with standard practice in agent-based modelling, where stochastic variability is characterised through repeated runs rather than exact replication of individual runs.  Twenty independent repetitions per parameter regime were used specifically for this purpose. The complete simulation output data are archived on Zenodo, providing a direct record of all results. |
| 2.15 | Version Control: Document the NetLogo version used and any version-specific behaviours. | The NetLogo version used is already documented in the Methodology section, which states that "NetLogo 6.4.0 was used" (with full citation). No version-specific behaviours requiring additional documentation were identified; the model uses standard NetLogo primitives available across NetLogo 6.x releases. |

## Responses to Reviewer 3:

| **Reviewer reference** | **Recommendations** | **Comments** |
| --- | --- | --- |
| 3.1 | In research, it's important to explain why previous findings vary. Is it due to limited empirical data or because they ignore systemic properties? This relates to your reasoning for choosing simulation. | This is a pertinent observation. The introduction has been amended to more explicitly address why previous findings vary, identifying two compounding factors: the systematic incompleteness of empirical fraud data, which introduces measurement inconsistency across organisational contexts by conflating fraud prevalence with detection effectiveness (Wang, 2011) and the tendency of individual-level frameworks to overlook systemic properties that only become visible through explicit modelling of organisational structure and interaction dynamics. This provides a clearer bridge to the rationale for the simulation approach. |
| 3.2 | Please add at least one paragraph of theoretical discussion predicting how the simulation results would change if a small-world structure were applied (e.g., "We expect the superlinear effect to be stronger because clustering accelerates behavioural transmission"). | The future research section has already been amended in this revision round to explicitly identify small-world and hierarchical network structures as a priority for subsequent work, in response to an earlier comment from this reviewer. In response to this specific comment, a theoretical prediction has been added to that passage outlining how small-world structures would be expected to influence the scaling relationships identified in the study. |
| 3.3 | Efficiency Issue (Point 2.14) requires improvements to the script by 15-20%. | Thank you for this comment. The manuscript has been reviewed for concision and targeted reductions have been made, primarily through condensing the theoretical discussion in the discussion section and tightening sentence structure throughout. We respectfully note, however, that the theoretical depth of the paper — particularly the critical realist and complexity-theoretic framing — is integral to its contribution rather than incidental, and that manuscript length has not been raised as a concern by other reviewers across two prior rounds of review. We do not consider a reduction of 15-20% to be achievable without materially compromising the paper's argument. |
