# Supplementary material for: Organisational scale and fraud volatility: An exploratory agent-based simulation of occupational fraud dynamics
Source: PLoS One. 2026 Jul 17;21(7):e0342964. doi: 10.1371/journal.pone.0342964 (PMC13378982; doi:10.1371/journal.pone.0342964)
Supplement: Attachment — Submitted filename: 2026-06-07 Response to reviewers PLOS One Fourth Round.docx [file pone.0342964.s010.docx]

## Responses to Reviewer 2:

| **Reviewer reference** | **Recommendations** | **Comments** |
| --- | --- | --- |
| 2.1 | Although the manuscript is in English, some issues of readability and consistency of terminology are apparent.  Weakness  • There are many long sentences with multiple clauses that can reduce clarity, especially for cross-disciplinary readers.  • Some terms do not appear to be entirely consistent throughout the text (e.g., the terms for perpetrators: “agents”, “employees”, “fraudulent agents”), which may confuse non-technical readers.  • Paragraphs tend to be long; these “blocks” of text make it difficult for readers to identify the main point of each paragraph.  Improvement Suggestions  • Make thorough style edits to:  o Breaking down long compound sentences into 2–3 shorter sentences with a clear subject–predicate–object–description structure;  o Reduce nonessential subordinate clauses or move explanatory clauses to separate sentences.  • Standardize key terminology, for example:   Use “agents (employees)” on the first mention, then consistently use “agents” or “employees” as the context requires.   Use consistent terms for fraud status (for example, “fraudulent agents” rather than changing terms).  • Review very long paragraphs and break them into shorter units, each with a single main idea. This will significantly improve readability in top-tier journals with a cross-disciplinary readership. | We thank the reviewer for these observations. Several improvements have been made in response. Key sections, including the abstract, introduction, discussion and conclusion, have been revised to simplify sentence structure, breaking compound sentences into shorter units with clearer subject-verb-object construction. Terminology has been standardised on first mention: agents are introduced as "agents (employees)" in the methodology section, with "agents" used consistently thereafter in technical descriptions and "employees" used where the organisational context is foregrounded. The term "fraudulent agents" is used consistently in the findings to describe agents satisfying all three fraud triangle conditions simultaneously.  We acknowledge that some longer paragraphs remain, particularly in the methodology section where the model description necessarily contains technical detail. We have reviewed these and where possible broken them into shorter units each centred on a single main idea. We believe the current version represents a substantial improvement in readability for cross-disciplinary readers while retaining the precision required for a technical model description. |
| 2.2 | However, to meet the standards of a top-tier journal, authors are advised to:  1. Streamline the narrative focus and reduce redundancy, especially in the introduction, literature review, discussion, and conclusion.  2. Clarifying theoretical structures and conceptual expectations before results, including through the formulation of propositions.  3. Strengthen reporting of ABM methodology and statistical analysis, including parameter justification and (where possible) additional sensitivity analysis.  4. Improve readability with more concise sentences, shorter paragraphs, and consistent terminology.  With substantial revisions to these aspects, the manuscript has strong potential to meet the quality expectations of top-tier journals in the fields of economic criminology and organizational complexity studies. | We thank the reviewer for this encouraging overall assessment and for the detailed and constructive feedback provided. The four areas identified have each been addressed substantively in the revised manuscript, as described in the responses to the individual comments above.  Narrative focus and redundancy have been reduced throughout, particularly in the introduction, literature review, discussion and conclusion, where overlapping material has been consolidated and cross-referenced rather than repeated.  Theoretical structure has been strengthened through the addition of three explicitly labelled theoretical expectations in the literature review and a restructured discussion that addresses each research question directly before expanding to theoretical and practical consequences.  ABM methodology and statistical reporting have been improved through the addition of three parameter tables, a clarified regression approach using regime means, and a sensitivity analysis for the volatility exclusion.  Importantly, the reviewer's question about independence and clustering prompted us to re-examine and correct the original analysis, which has refined the findings and we believe has strengthened the paper's contribution.  Readability has been improved through sentence simplification, terminology standardisation, and paragraph restructuring throughout the manuscript.  We believe the revised manuscript addresses the reviewer's concerns comprehensively and we hope it now meets the standards expected for publication. |
| 2.3 | Consider summarizing the title into a more focused two-clause form, for example (just  an example):  ▪ “Organizational Scale and Occupational Fraud Dynamics: An Exploratory Agent-  Based Simulation”  ▪ or “Non-linear Scaling of Occupational Fraud with Organizational Size: An Agent-  Based Simulation Study”.  • Add an explicit emphasis that this study is exploratory and simulation-based to avoid  readers' expectations of empirical calibration or formal hypothesis testing.  • If the journal emphasizes practical contributions, it may be worth considering including  the terms “fraud risk” or “fraud volatility” in the title to make the implications for risk  management more immediately apparent. | The title has been revised from the previous submission to address these points. The current title — "Organisational Scale and Fraud Volatility: An Exploratory Agent-Based Simulation of Occupational Fraud Dynamics" — adopts the two-clause structure suggested, signals the exploratory and simulation-based nature of the study explicitly, and includes the term "fraud volatility" to make the practical risk management contribution immediately apparent to readers. We believe the revised title accurately represents the scope and contribution of the study. |
| 2.4 | Summarize the abstract into 3–4 very short paragraphs (or 1 structured paragraph)  with the following pattern:  a. Background and gaps;  b. Methods (ABM, organizational size range, key parameters);  c. The main quantitative findings (scale exponent values and their interpretation)  and qualitative findings (the role of scale and networks);  d. Main theoretical and practical implications.  • Consider stating explicitly, near the beginning of the abstract, that this study is an  “exploratory, conceptual agent-based simulation not empirically calibrated to real-  world data.”  • Simplify long compound sentences into shorter sentences with a clear subject–verb–  object–adverb structure to improve clarity for readers across disciplines.• Clarify the distinction between theoretical contributions (e.g., incorporating  organizational scale and volatility into the framework of fraud as a systemic  phenomenon) and practical consequences (e.g., the need for non-linearly increasing  control strengthening in large organizations). | We thank the reviewer for this suggestion. The abstract has been revised to follow the recommended four-part structure: (a) background and gap; (b) methods, including an explicit statement that the study is an exploratory, conceptual simulation not empirically calibrated to real-world data; (c) main quantitative findings with scaling exponent values; and (d) theoretical and practical implications.  The final paragraph has been revised to separate these clearly: the theoretical contribution concerns establishing organisational scale as a structural mechanism and fraud volatility as a distinct dimension of disproportionate risk; the practical implication concerns the need for a different approach to managing volatility, grounded in affordance mapping rather than case-led detection. Sentence structure has been simplified throughout. |
| 2.5 | Summarize the general context sections (national fraud statistics, psychological  impact) into one or two paragraphs that directly address the main empirical issue,namely the ambiguity of the relationship between organizational size and fraud  prevalence.  • Clarify and accelerate the transition to the main gap:  o that survey studies and ACFE reports provide inconsistent findings regarding the  relationship between organizational scale and fraud,  o and that detection limitations make it difficult to use observational data to  confidently test scaling patterns.  • Move the research questions statement to an earlier part of the introduction, for  example after the first 2–3 paragraphs, so that the reader immediately has a map of  the direction of the study.  • Add an introductory concluding paragraph that explicitly presents: (1) the gap; (2) the  approach; (3) the main contribution in 3–4 concise sentences, with clear declarative  language. | The introduction has been substantially restructured in response to these suggestions. The opening context has been condensed into two paragraphs that move quickly to the central empirical issue: the inconsistency in survey and ACFE findings regarding the relationship between organisational size and fraud, and the detection limitations that make this inconsistency difficult to resolve using observational data alone.  The three research questions have been moved to immediately follow this opening discussion, giving the reader an early map of the study's direction.  The introduction now closes with a declarative paragraph that explicitly presents the gap in existing simulation-based research, the agent-based modelling approach adopted, and the study's three main contributions. |
| 2.6 | Reorganize the literature review thematically with sharper subheadings, for example:  ▪ “Fraud against Organizations and Organizational Scale”;  ▪ “Individualistic Accounts: Fraud Triangle and Its Critiques”;  ▪ “Fraud as a Systemic and Complex Phenomenon”;  ▪ "Agent-Based Modeling in Criminology and Economic Crime".  • After the primary literature review, explicitly formulate 2–4 theoretical propositions or  “expectations” (although not called formal hypotheses), for example:  ▪ That the average level of fraud will increase superlinearly with the size of the  organization;  ▪ That fraud volatility is increasing faster than average;  ▪ That changes in network connectivity and social influence will moderate the  scale–fraud relationship. This formulation will help the reader assess the extent  to which the simulation results align or contradict expectations.  • Consider moving some of the philosophical discussion of critical realism to the  discussion section so that the introduction and literature review remain focused on  establishing a more immediate empirical and theoretical context for the model. | The literature review has been reorganised into four thematic subsections with sharper headings, following the structure suggested: organisational fraud and scale; the fraud triangle and its critiques; fraud as a systemic and complex phenomenon; and agent-based modelling in criminology and economic crime. A dedicated "Theoretical Expectations" subsection now follows the review, setting out three theoretically grounded expectations derived from the literature. These are explicitly framed as conjectures rather than formal hypotheses, and the Discussion section addresses each in turn, including noting where results did not support the expectation.  The philosophical discussion of critical realism has been relocated to the Discussion section, where it is addressed under a dedicated subheading on positioning within critical realism and complexity theory, keeping the literature review focused on the empirical and theoretical context for the model. |
| 2.7 | Consider organizing the methods section according to the ODD framework or an equivalent framework recognized in the ABM community, so that the model  description is easier to review and replicate.  • Add a table or appendix summarizing all parameters (range, tested values,  justification, and theoretical implications) beyond the existing Table 1. This table  should separate structural, behavioral, and output parameters.  • Expand the explanation regarding sensitivity analysis:  ▪ Explain whether the authors have tested alternative variations of the initial  probability (e.g., 0.3 or 0.7) and whether the scaling pattern remains stable;  ▪ If you haven't already, consider adding a short experiment or at least an explicit  discussion of the possible impact of the variation on the results.  • Clarify the structure of the simulation data and justify the use of standard OLS  regression on log–log output: whether each point in the regression represents an  average over 20 runs per configuration, or a run-by-run observation; and how the  authors address issues of independence and clustering.  • In the verification/validation section, describe the steps of exploring the model's  behavior (e.g., checking face validity, testing extreme parameters, or checking whether the output returns to the expected pattern at certain parameters), with short  examples so that the reader has more confidence in the model's robustness. | We thank the reviewer for these detailed suggestions, which have prompted several substantive improvements to the methodology and findings.  On the ODD framework: Rather than restructuring the section around ODD headings, we have ensured that the equivalent content is present and clearly signposted: the model's purpose, entities and state variables, process overview, initialisation, and experimental design are all described in the current structure, and full replication materials are available in the public Zenodo repository.  Three new tables have been added to the methodology section to provide a comprehensive parameter summary beyond the existing operational definitions table. Table 2 covers structural parameters, Table 3 covers behavioural parameters, and Table 4 covers output measures. Each table includes tested values, justification for the selected range, and theoretical implications.  The reviewer's question about regression structure prompted us to re-examine the original analysis. The original regressions were estimated on all 9,000 individual simulation runs rather than on regime means. As the reviewer correctly identified, observations sharing the same parameter configuration are not independent, which risks underestimating standard errors. We therefore re-ran all regressions on regime means aggregated across the 20 repetitions per parameter configuration, yielding 450 independent observations. This corrected approach produces somewhat different results for mean fraud scaling: the exponent changes from β = 1.245 to β = 1.004, indicating approximately linear rather than superlinear scaling of mean fraud. The volatility and peak fraud exponents are essentially unchanged. These corrected results are reported throughout the revised manuscript and the discussion and conclusions have been updated accordingly. We are grateful to the reviewer for prompting this correction, which we believe strengthens the paper's analytical rigour and produces a more theoretically coherent set of findings.  On sensitivity analysis of initial probabilities: the initial probabilities were fixed at 0.5 to provide a neutral baseline. Variations such as 0.3 or 0.7 were not formally tested. However, the 10,000-tick run length means initial conditions are effectively burned in before outcomes are recorded, and the wide range of q_avg values tested encompasses the dynamics that alternative initial probabilities would primarily affect. We acknowledge this as a limitation and have added a discussion of the expected impact of initial probability variation to the methodology section.  On verification: the verification section describes systematic parameter exploration across the full parameter space. The use of 450 distinct parameter regimes, each run 20 times, constitutes a comprehensive examination of model behaviour across a wide range of structural and behavioural conditions. |
| 2.8 | Add a short subsection providing a simple numerical example: for example, an  illustration of how the average fraud rate and volatility differ for N = 100 vs. N = 3,200,  based on representative simulation results, so that readers understand the effect size  in more concrete units.  • If technically feasible, consider displaying time series (change in the number of fraud  agents per tick) for two or three contrasting configurations (e.g. small vs large  organizations with low and high q_avg) to visualize the differences in volatility patterns  that are one of the key claims.• Clarify in the text whether the reported exponent numbers are from regressions on  the inter-run average per configuration or across individual runs, and explain the  implications of this approach for the interpretation of R² and p-values. | An "Illustrative example" subsection has been added to the Findings section. This presents predicted fraud outcomes for two contrasting organisational sizes — N = 100 and N = 3,200, a 32-fold difference — derived from the regression models at representative mid-range parameter values. The example shows that mean fraud and peak fraud both increase approximately in proportion to size, while the standard deviation of fraud counts rises from approximately 1.1 agents at N = 100 to approximately 124 agents at N = 3,200, against a proportional expectation of around 35 agents. This contrast illustrates the central finding in concrete terms accessible to readers across disciplines.  Regarding time series visualisations: tick-by-tick data was not retained from the original simulations, which recorded summary statistics per run rather than per-tick counts. We acknowledge this as a limitation and note it as a direction for future work.  The clarification of regression structure is addressed in the response to an earlier comment above. |
| 2.9 | Consider restructuring the discussion into subsections with informative headings, for  example:  a. “Revisiting the Research Questions”;  b. “Fraud as a System-Level Phenomenon”;c. “Implications for Fraud Theory (Fraud Triangle and Beyond)”;  d. “Implications for Practice and Policy”;  e. "Positioning within Critical Realism and Complexity Theory".  • In the first subsection, explicitly answer each research question by referring directly  to the main results, before expanding the discussion to theoretical and practical  consequences.  • Reduce the repetition of narratives regarding criticism of the fraud triangle and the  position of critical realism by referring to the previous section, so that the discussion  appears sharper and more economical.  • Explicitly state what the core theoretical contributions are (e.g., including  organizational scale and volatility as explicit dimensions of fraud dynamics;  demonstrating that the assumption of per capita linearity is often misleading) and how  these contributions complement, rather than replace, individual frameworks such as  the fraud triangle.  • Summarize the conclusion into 3–4 paragraphs that each focus on:  a. Short answers to research questions related to scaling fraud with organizational  size;  b. Major theoretical contributions to the fraud, complexity, and economic  criminology literature;  c. Key practical implications for control design and governance;  d. The most priority direction for further research.  • Avoid repeating methodological details and critical arguments presented in previous  sections; focus on the most important take-home messages.  • Use sentences with a shorter, clearer structure (subject–predicate–object–adverb) to  improve readability, especially for readers from other disciplines. | The discussion has been substantially restructured in response to these suggestions. It is now organised around five subheadings: "Revisiting the Research Questions"; "Fraud as a System-Level Phenomenon"; "Implications for Fraud Theory"; "Positioning within Critical Realism and Complexity Theory." The first subsection directly answers each of the three research questions by reference to the regression results before expanding to broader theoretical and practical consequences. Repetition of the fraud triangle critique has been reduced by cross-referencing the literature review rather than restating the argument. The critical realism discussion is confined to its own subsection and kept concise.  The theoretical contributions are now stated explicitly, including the finding that per capita linearity holds for mean fraud but fails substantially for volatility, and the complementary rather than replacement relationship with the fraud triangle is made explicit.  The conclusion has been restructured into four focused paragraphs addressing: the research question answers; theoretical contributions; practical implications for control design; and priority directions for future research. Methodological details have been removed from the discussion and conclusion, and sentence structure has been simplified throughout to improve accessibility for readers across disciplines. |
| 2.10 | Group the limitations into 3–4 main categories, for example:▪ (1) Organizational structure and network;  ▪ (2) Representation of agent behavior;  ▪ (3) Empirical calibration and validation;  ▪ (4) Scope of fraud (internal only, does not include external perpetrators).  • In each category, summarize 1–2 sentences explaining the limitations followed by 1  sentence indicating the most immediate direction for further research.  • Highlight the 2–3 most important further research priorities (e.g., empirical integration  of network structures; calibration against specific organizational data; use of the  model as a test-bed for policy interventions), and place these priorities at the end of  the section as concrete guidance for future researchers. | The limitations section has been reorganised into four thematic categories following the structure suggested: organisational structure and network realism; agent behaviour and parameterisation; empirical calibration and external validation; and scope of modelled fraud. Each category contains a concise statement of the limitation and a sentence identifying the most immediate direction for future research. A dedicated closing subsection identifies the three most important research priorities: incorporating empirically observed network structures to test the robustness of the scaling relationships; calibrating model parameters against specific organisational data to bridge the gap between exploratory simulation and practical application; and using the calibrated model as a test-bed for policy interventions to provide theoretically grounded guidance for practitioners. These are presented as concrete priorities rather than general observations. |
| 2.11 | Summarize the conclusion into 3–4 paragraphs that each focus on:  a. Short answers to research questions related to scaling fraud with organizational  size;  b. Major theoretical contributions to the fraud, complexity, and economic  criminology literature;  c. Key practical implications for control design and governance;  d. The most priority direction for further research.  • Avoid repeating methodological details and critical arguments presented in previous  sections; focus on the most important take-home messages.  • Use sentences with a shorter, clearer structure (subject–predicate–object–adverb) to  improve readability, especially for readers from other disciplines. | This comment has been addressed as part of the broader restructuring described in the response to Comment 6. The conclusion has been rewritten as four focused paragraphs: the first answers the three research questions directly with the key β values; the second states the theoretical contributions to economic criminology and organisational complexity research; the third summarises the practical implications for fraud control design and governance; and the fourth identifies empirical testing of the scaling relationships as the priority direction for future research. Methodological details and critical arguments from earlier sections have been removed. Sentence structure has been simplified throughout to improve readability for readers across disciplines |
