# Supplementary material for: Organisational scale and fraud volatility: An exploratory agent-based simulation of occupational fraud dynamics
Source: PLoS One. 2026 Jul 17;21(7):e0342964. doi: 10.1371/journal.pone.0342964 (PMC13378982; doi:10.1371/journal.pone.0342964)
Supplement: Peer review history

# Peer review: Decision Letter 0

**DOI:** 10.1371/journal.pone.0342964.r001

**Authors:** Annesha Sil

3 Mar 2026

PONE-D-25-65420

Detecting emergent organisational fraud dynamics using agent-based modelling

PLOS One

Dear Dr. Freeman,

Thank you for submitting your manuscript to PLOS ONE. After careful consideration, we feel that it has merit but does not fully meet PLOS ONE’s publication criteria as it currently stands. Therefore, we invite you to submit a revised version of the manuscript that addresses the points raised during the review process.

The reviewers have raised concerns around the methodology and analysis and we request you to address all their concerns in your revised manuscript.

Please submit your revised manuscript by Apr 17 2026 11:59PM. If you will need more time than this to complete your revisions, please reply to this message or contact the journal office at plosone@plos.org. When you're ready to submit your revision, log on to https://www.editorialmanager.com/pone/ and select the 'Submissions Needing Revision' folder to locate your manuscript file.

Please include the following items when submitting your revised manuscript:

- A letter that responds to each point raised by the academic editor and reviewer(s). You should upload this letter as a separate file labeled 'Response to Reviewers'.
- A marked-up copy of your manuscript that highlights changes made to the original version. You should upload this as a separate file labeled 'Revised Manuscript with Track Changes'.
- An unmarked version of your revised paper without tracked changes. You should upload this as a separate file labeled 'Manuscript'.

If you would like to make changes to your financial disclosure, please include your updated statement in your cover letter. Guidelines for resubmitting your figure files are available below the reviewer comments at the end of this letter.

If applicable, we recommend that you deposit your laboratory protocols in protocols.io to enhance the reproducibility of your results. Protocols.io assigns your protocol its own identifier (DOI) so that it can be cited independently in the future. For instructions see: https://journals.plos.org/plosone/s/submission-guidelines#loc-laboratory-protocols. Additionally, PLOS ONE offers an option for publishing peer-reviewed Lab Protocol articles, which describe protocols hosted on protocols.io. Read more information on sharing protocols at https://plos.org/protocols?utm_medium=editorial-email&utm_source=authorletters&utm_campaign=protocols.

We look forward to receiving your revised manuscript.

Kind regards,

Annesha Sil, Ph.D.

Staff Editor

PLOS One

Journal Requirements:

When submitting your revision, we need you to address these additional requirements.

1. Please ensure that your manuscript meets PLOS ONE's style requirements, including those for file naming. The PLOS ONE style templates can be found at

https://journals.plos.org/plosone/s/file?id=wjVg/PLOSOne_formatting_sample_main_body.pdf and https://journals.plos.org/plosone/s/file?id=ba62/PLOSOne_formatting_sample_title_authors_affiliations.pdf

2. Please note that PLOS One has specific guidelines on code sharing for submissions in which author-generated code underpins the findings in the manuscript. In these cases, we expect all author-generated code to be made available without restrictions upon publication of the work. Please review our guidelines at https://journals.plos.org/plosone/s/materials-and-software-sharing#loc-sharing-code and ensure that your code is shared in a way that follows best practice and facilitates reproducibility and reuse.

3. You have indicated that data is available from [Zenodo.org]. Please can we ask you to provide us with a general contact email address for the data requests, so readers can request access in perpetuity. If a general email is not available please provide a link to a website where readers can obtain access to data.

4. When completing the data availability statement of the submission form, you indicated that you will make your data available on acceptance. We strongly recommend all authors decide on a data sharing plan before acceptance, as the process can be lengthy and hold up publication timelines. Please note that, though access restrictions are acceptable now, your entire data will need to be made freely accessible if your manuscript is accepted for publication. This policy applies to all data except where public deposition would breach compliance with the protocol approved by your research ethics board. If you are unable to adhere to our open data policy, please kindly revise your statement to explain your reasoning and we will seek the editor's input on an exemption. Please be assured that, once you have provided your new statement, the assessment of your exemption will not hold up the peer review process.

5. If the reviewer comments include a recommendation to cite specific previously published works, please review and evaluate these publications to determine whether they are relevant and should be cited. There is no requirement to cite these works unless the editor has indicated otherwise.

[Note: HTML markup is below. Please do not edit.]

Reviewers' comments:

Reviewer's Responses to Questions

**Comments to the Author**

1. Is the manuscript technically sound, and do the data support the conclusions?

The manuscript must describe a technically sound piece of scientific research with data that supports the conclusions. Experiments must have been conducted rigorously, with appropriate controls, replication, and sample sizes. The conclusions must be drawn appropriately based on the data presented.

Reviewer #1: Yes

Reviewer #2: Yes

Reviewer #3: Yes

Reviewer #4: Partly

Reviewer #5: Yes

**********

2. Has the statistical analysis been performed appropriately and rigorously?

Reviewer #1: No

Reviewer #2: No

Reviewer #3: Yes

Reviewer #4: Yes

Reviewer #5: Yes

**********

3. Have the authors made all data underlying the findings in their manuscript fully available?

The PLOS Data policy requires authors to make all data underlying the findings described in their manuscript fully available without restriction, with rare exception (please refer to the Data Availability Statement in the manuscript PDF file). The data should be provided as part of the manuscript or its supporting information, or deposited to a public repository. For example, in addition to summary statistics, the data points behind means, medians and variance measures should be available. If there are restrictions on publicly sharing data—e.g. participant privacy or use of data from a third party—those must be specified.

Reviewer #1: Yes

Reviewer #2: Yes

Reviewer #3: Yes

Reviewer #4: Yes

Reviewer #5: Yes

**********

4. Is the manuscript presented in an intelligible fashion and written in standard English?

PLOS ONE does not copyedit accepted manuscripts, so the language in submitted articles must be clear, correct, and unambiguous. Any typographical or grammatical errors should be corrected at revision, so please note any specific errors here.

Reviewer #1: Yes

Reviewer #2: Yes

Reviewer #3: Yes

Reviewer #4: Yes

Reviewer #5: Yes

**********

5. Review Comments to the Author

Please use the space provided to explain your answers to the questions above. You may also include additional comments for the author, including concerns about dual publication, research ethics, or publication ethics. (Please upload your review as an attachment if it exceeds 20,000 characters)

Reviewer #1: Generalizability Concerns: Results may not apply to many real-world fraud scenarios (all results from simulation)

No empirical validation against real organizational fraud data, been acknowledged but not adequately addressed. Please revise.

Visualization: Please add a conceptual framework diagram (research model) at the end of the Introduction or the beginning of the Methods to make it easier for readers to understand the relationship between variables. Do not forget to include adequate explanations upon the model.

Statistical Test

Parameter Justification - q_avg values (0.15 vs 0.95) lack empirical grounding; no sensitivity analysis. Please provide the references as well as the sensitivity analysis

Robustness Testing - No sensitivity analysis or alternative model assumptions tested. Please conduct the testing, and

provide us the results and intepretation

Reviewer #2: Critical Evaluation: The authors' conclusions are generally consistent with the results – they don't overdo it by promising absolute certainty, even acknowledging the complex nature of open systems. However, there are some critical caveats:

• Generalization vs Context:The conclusion confirms the emergent nature of fraud, but it should be accompanied by a contextual reminder that it is based on simulation. In the original text, the caveat "based on our model" was not explicitly stated when stating, for example, "dense networks + high influence = vulnerable." In a scientific report, this should be more explicit to avoid the impression that this is an empirical law. Fortunately, the authors immediately follow up with recommendations for further research for validation, indicating they understand these results are merely hypotheses that need further testing.

• Traceability to Initial Destination:The research objectives (asked in the introduction as questions) are answered in the conclusions. For example, they asked whether fraud increases proportionally with size—the answer is: not always, the scaling pattern depends on the conditions. They asked about the role of connectivity and influence—the answer is: very influential, resulting in different systemic behaviors. So, internally, the conclusions are consistent with the research questions.

• Scientific Contribution: The authors claim to open new avenues of fraud studies. From a critical perspective, these contributions are preliminary. They have demonstrated proof of concept that ABM can generate multi-scale emergent fraud behavior. However, until empirical validation is provided, these results are hypothetical. The contribution to fraud theory lies in supporting a multi-level perspective (Bunge, etc.)—but again, this is more of a model illustration than empirical evidence. It would be better for authors to state that their findings support a particular theoretical framework but still require testing. For example, it might be helpful to state, "This study provides preliminary support for perspective X" rather than implying that the old perspective should be abandoned.

• Practical Recommendations:The advice provided is sound: focus on systemic factors, monitor volatility. However, some recommendations may be difficult to understand without implementation guidance. For example, the phrase "snapshot measures risk obscuring latent escalation."That's true, but what are the concrete recommendations? Perhaps it should be added: "Therefore, management should monitor long-term trends and the distribution of cases across the organization, not just the annual total." This explanation is missing from the text, making the recommendations seem abstract.

Additional Suggestions:1. Write a Limitations section before the conclusion. This is academically important. Explicitly mention the limitations of the model (which we discussed in the methods). Examples include the assumption of static variables, the absence of external factors, etc., as well as data limitations (hidden fraud). By outlining these in the conclusion, the reader is assured that the authors have not overlooked the study's shortcomings. 2. Prioritize Further Empirical Validation. The authors have recommended mirroring real networks and comparing data.[38]This suggestion is very appropriate. Additionally, they could recommend collaborating with companies to test-bedding this model. For example, applying the model to internal data from a large company (with adjusted parameters) to see if the model's predictions match the areas of vulnerability identified. This would bridge the model-reality gap. 3. Don't Forget the Humans: While this research is about systems, ultimately, policies must be implemented by people. Recommendations could address the importance of an effective integrity culture program. The model results clearly show that a highly contagious culture (attitude) can have detrimental effects. Therefore, strengthening the integrity culture of large companies through training, rotating leaders to ensure the company's values are shared, etc., is a practical recommendation aligned with the findings (reducing effective q_avg). 4. Alignment with Regulations: Organizations are often subject to anti-fraud regulations (e.g., mandatory internal audits for public companies). The authors could recommend that regulators address the scale factor: for example, requiring mid-sized companies to have adequate audit/investigation functions due to their vulnerability. This public policy recommendation would demonstrate the broad impact of the findings.

Reviewer #3: The manuscript addresses an interesting and relevant topic, namely Detecting emergent organisational fraud dynamics using agent-based modelling. The use of complexity theory and agent-based modelling (ABM) to examine organisational fraud dynamics is promising and has the potential to make a meaningful theoretical contribution. However, several substantive revisions are required to improve clarity, methodological transparency, and theoretical rigor.

1. Conceptual Framework and Theoretical Gap

The manuscript would benefit from a sharper articulation of the theoretical gap. The authors are encouraged to explicitly state:

Which dominant or mainstream approaches to organisational fraud detection are being challenged (e.g., linear risk models, regression-based approaches).

What novel insights complexity theory and scaling laws offer as an alternative analytical lens.

The discussion of Complexity Theory and Scaling Laws should be more explicitly linked to organisational fraud dynamics, rather than remaining largely implicit.

In particular, the discussion related to Fraud Field Theory requires further clarification. The manuscript should explain more clearly why and how the present findings undermine the conventional assumptions of this theory, especially in the Discussion section, including the underlying logic of reasoning.

2. Operational Definitions and Variables

The manuscript currently lacks a table of operational definitions.

A dedicated table defining key variables, parameters, and agent attributes used in the model is strongly recommended.

This would significantly enhance transparency and replicability, particularly for readers less familiar with agent-based modelling.

3. Methodology: Population, Sample, and Experimental Design

The method section should be presented more systematically, preferably in tabular form, to improve clarity.

The description of simulations ranging “from 50 to 6,400 employees across six experimental regimes” requires further elaboration, particularly:

How these organisational sizes were determined.

Whether they are theoretically motivated, empirically informed, or arbitrarily selected.

How the six experimental regimes differ from one another in structural or behavioural assumptions.

The manuscript refers to “population” and “sample” implicitly; however, in the context of ABM, these concepts should be explicitly clarified to avoid conceptual ambiguity.

4. Definitions of Organisational Size

The repeated use of terms such as “larger” and “smaller” organisations would benefit from clearer operational and conceptual definitions, especially given the analytical modelling approach.

Explicit thresholds or criteria for categorising organisational size (small, medium, large) should be stated and justified.

5. Results Interpretation and Discussion

The findings related to linear, sublinear, superlinear, and volatile phases are intriguing; however:

The manuscript should provide a clearer explanation of why these patterns emerge, particularly in the Discussion section.

The rationale behind why such patterns are not detectable through global regressions but are revealed via banded analysis should be more explicitly articulated.

The argument that medium-sized organisations face the highest fraud risk, as observed in regimes R2, R3, R5, and R6, is important but requires:

Stronger theoretical justification.

Additional support from prior empirical studies or closely related literature to strengthen the claim.

6. Limitations Section

In the Limitations section, the statement beginning with “Like all agent-based models, this study relies on a simplified representation of an organisational system…” does not require citations.

This is a widely accepted and self-evident methodological limitation in ABM research.

Removing unnecessary citations would improve conciseness and focus.

7. Practical and Policy Implications

The implications section would benefit from stronger empirical and theoretical grounding, particularly for claims such as:

Organisations with dense networks, high susceptibility to influence, and rapid growth being inherently prone to disproportionate fraud escalation.

Where possible, these claims should be supported by: Recent empirical studies, or Explicit linkage to established theories in organisational behaviour, criminology, or complexity science.

8. References and Literature Update

The reference list should be updated to reflect more recent literature, ideally within the last 5 years, or a maximum of 10 years, except for clearly established grand theories.

Several cited works are relatively dated and should either be: Justified as foundational (e.g., Bunge, Bettencourt et al.), or

Supplemented with more recent studies that demonstrate the contemporary relevance of the theoretical framework.

Reviewer #4: Dear Authors,

It was a pleasure to read and review your paper that I found interesting and with an important scientific potential. I appreciate the relevant sources from the literature review, but I encourage you to include more recent sources.

Also, I appreciate your research idea, of using simulations, and I confess that these results could be useful for further research that could use them in artificial intelligence practice. For this reason, your results should be more robustness and be generalized. For this reason, I appreciate to better describe the independent variables, their distribution, and how they could interact in order to support the Table 1 results (for these table, please give more references and explain how do you support the scenarios.

The results are interesting but give more practical applications and connect the results with practice.

Best regards!

Reviewer #5: PONE-D-25-65420

Title: Detecting emergent organisational fraud dynamics using agent-based modelling.

Comment 1: The claim "fraud against organizations is poorly understood" is too absolute without a broader discussion of the organizational fraud literature, such as occupational fraud, corporate crime, and governance fraud. Empirical and methodological contributions have not been clearly distinguished. The practical relevance to modern organizations, such as digital organizations and network firms, has not been established.

Recommendation 1: Please add the literature on organizational fraud and corporate crime. Then emphasize the novelty of the research on the first scaling-based ABM of organizational fraud. Link it to the context of contemporary organizations (large complex organizations).

Comment 2: The review is too lengthy on the history of the fraud triangle but relatively short on organizational criminology and complexity crime studies. The broader criminal ABM literature, such as crime diffusion models and network contagion crime, has not been discussed. The study's position on social ABM in general has not been clarified.

Recommendation 2: The reviewer suggests adding the criminal/organizational ABM literature. Please summarize the section on the history of the fraud triangle. Emphasize the position of the ABM in criminology and computational social science.

Comment 3: The discussion of scaling focuses too much on cities and biology; the relevance of organizations needs to be strengthened. The concept of phase transition has not been formally explained (critical thresholds). There is no operational definition of scaling in the context of fraud.

Recommendation 3: Add literature on organizational/firm scaling. Explain the concept of phase transition more formally. Define fraud scaling explicitly.

Comment 4: The definition of band sizes (small/medium/large) is not justified. The band boundaries appear arbitrary. There is no statistical test for differences between bands. Causal interpretations are sometimes too speculative.

Recommendation 4: Explain the basis for band selection. Add sensitivity band thresholds. Use formal breakpoint analysis or piecewise regression.

Comment 5: Some interpretations are too deterministic (“organization predisposed”). There is no measure of simulation uncertainty. There is no visualization of a global dynamics summary.

Recommendation 5: Add simulation confidence intervals.

Add a summary figure regime map. Avoid strong causal claims without empirical validation.

Comment 6: The philosophical discussion is too long. Some claims go beyond simulation evidence. The relationship to antifraud practices is not yet operational.

Recommendation 6: Summarize the philosophical section. Add concrete practical implications. Add limits to the simulation's interpretation.

Comment 7: The model is not empirically calibrated. The organizational structure is unrealistic. Homogeneous agents. Single fraud type. No learning/adaptation. No enforcement/control agents.

Recommendation 7: Add limitations regarding external validity, structural realism, behavioral realism, and parameter uncertainty.

Comment 8: Some claims are too general. The empirical implications are unclear. The future research agenda is not concrete enough.

Recommendation 8: Clarify the methodological contribution. Clarify the contribution to fraud theory. Add an empirical research roadmap.

Reviewer's Decision:

The final recommendation is a Major Revision.

**********

6. PLOS authors have the option to publish the peer review history of their article (what does this mean?). If published, this will include your full peer review and any attached files.

If you choose “no”, your identity will remain anonymous but your review may still be made public.

**Do you want your identity to be public for this peer review?**  For information about this choice, including consent withdrawal, please see our Privacy Policy.

Reviewer #1: **Yes:** Armanto Witjaksono

Reviewer #2: No

Reviewer #3: **Yes:** Imang Dapit Pamungkas

Reviewer #4: No

Reviewer #5: No

**********

[NOTE: If reviewer comments were submitted as an attachment file, they will be attached to this email and accessible via the submission site. Please log into your account, locate the manuscript record, and check for the action link "View Attachments". If this link does not appear, there are no attachment files.]

To ensure your figures meet our technical requirements, please review our figure guidelines: https://journals.plos.org/plosone/s/figures

You may also use PLOS’s free figure tool, NAAS, to help you prepare publication quality figures: https://journals.plos.org/plosone/s/figures#loc-tools-for-figure-preparation.

NAAS will assess whether your figures meet our technical requirements by comparing each figure against our figure specifications.

Attachment Submitted filename: Reviewer Attachments for Manuscript Number PONE-D-25-65420.pdf

# Author comment: Author response to Decision Letter 1

**DOI:** 10.1371/journal.pone.0342964.r002

19 Mar 2026

Two documents have been uploaded: 1. A new covering letter to the editor 2. Response to all reviewer comments.

Attachment Submitted filename: 2026-03-17 Response to reviewers PLOS One-1.docx

# Peer review: Decision Letter 1

**DOI:** 10.1371/journal.pone.0342964.r003

**Authors:** Annesha Sil, Vanessa Carels

8 Apr 2026

PONE-D-25-65420R1

Scaling of occupational fraud with organisational size: Evidence from an agent-based model

PLOS One

Dear Dr. Freeman,

Thank you for submitting your manuscript to PLOS ONE. After careful consideration, we feel that it has merit but does not fully meet PLOS ONE’s publication criteria as it currently stands. Therefore, we invite you to submit a revised version of the manuscript that addresses the points raised during the review process.

Please submit your revised manuscript by May 22 2026 11:59PM. If you will need more time than this to complete your revisions, please reply to this message or contact the journal office at plosone@plos.org. When you're ready to submit your revision, log on to https://www.editorialmanager.com/pone/ and select the 'Submissions Needing Revision' folder to locate your manuscript file.

Please include the following items when submitting your revised manuscript:

- A letter that responds to each point raised by the academic editor and reviewer(s). You should upload this letter as a separate file labeled 'Response to Reviewers'.
- A marked-up copy of your manuscript that highlights changes made to the original version. You should upload this as a separate file labeled 'Revised Manuscript with Track Changes'.
- An unmarked version of your revised paper without tracked changes. You should upload this as a separate file labeled 'Manuscript'.

If you would like to make changes to your financial disclosure, please include your updated statement in your cover letter. Guidelines for resubmitting your figure files are available below the reviewer comments at the end of this letter.

If applicable, we recommend that you deposit your laboratory protocols in protocols.io to enhance the reproducibility of your results. Protocols.io assigns your protocol its own identifier (DOI) so that it can be cited independently in the future. For instructions see: https://journals.plos.org/plosone/s/submission-guidelines#loc-laboratory-protocols. Additionally, PLOS ONE offers an option for publishing peer-reviewed Lab Protocol articles, which describe protocols hosted on protocols.io. Read more information on sharing protocols at https://plos.org/protocols?utm_medium=editorial-email&utm_source=authorletters&utm_campaign=protocols.

As the corresponding author, your ORCID iD is verified in the submission system and will appear in the published article. PLOS supports the use of ORCID, and we encourage all coauthors to register for an ORCID iD and use it as well. Please encourage your coauthors to verify their ORCID iD within the submission system before final acceptance, as unverified ORCID iDs will not appear in the published article. *Only*  the individual author can complete the verification step; PLOS staff *cannot*  verify ORCID iDs on behalf of authors.

We look forward to receiving your revised manuscript.

Kind regards,

Vanessa Carels

Staff Editor

PLOS One

Journal Requirements:

If the reviewer comments include a recommendation to cite specific previously published works, please review and evaluate these publications to determine whether they are relevant and should be cited. There is no requirement to cite these works unless the editor has indicated otherwise.

[Note: HTML markup is below. Please do not edit.]

Reviewers' comments:

Reviewer's Responses to Questions

**Comments to the Author**

1. If the authors have adequately addressed your comments raised in a previous round of review and you feel that this manuscript is now acceptable for publication, you may indicate that here to bypass the “Comments to the Author” section, enter your conflict of interest statement in the “Confidential to Editor” section, and submit your "Accept" recommendation.

Reviewer #1: All comments have been addressed

Reviewer #2: (No Response)

Reviewer #3: All comments have been addressed

Reviewer #4: All comments have been addressed

Reviewer #5: All comments have been addressed

**********

2. Is the manuscript technically sound, and do the data support the conclusions?

The manuscript must describe a technically sound piece of scientific research with data that supports the conclusions. Experiments must have been conducted rigorously, with appropriate controls, replication, and sample sizes. The conclusions must be drawn appropriately based on the data presented.

Reviewer #1: Yes

Reviewer #2: Yes

Reviewer #3: No

Reviewer #4: Yes

Reviewer #5: Yes

**********

3. Has the statistical analysis been performed appropriately and rigorously?

Reviewer #1: Yes

Reviewer #2: No

Reviewer #3: No

Reviewer #4: Yes

Reviewer #5: Yes

**********

4. Have the authors made all data underlying the findings in their manuscript fully available?

The PLOS Data policy requires authors to make all data underlying the findings described in their manuscript fully available without restriction, with rare exception (please refer to the Data Availability Statement in the manuscript PDF file). The data should be provided as part of the manuscript or its supporting information, or deposited to a public repository. For example, in addition to summary statistics, the data points behind means, medians and variance measures should be available. If there are restrictions on publicly sharing data—e.g. participant privacy or use of data from a third party—those must be specified.

Reviewer #1: Yes

Reviewer #2: Yes

Reviewer #3: No

Reviewer #4: Yes

Reviewer #5: Yes

**********

5. Is the manuscript presented in an intelligible fashion and written in standard English?

PLOS ONE does not copyedit accepted manuscripts, so the language in submitted articles must be clear, correct, and unambiguous. Any typographical or grammatical errors should be corrected at revision, so please note any specific errors here.

Reviewer #1: Yes

Reviewer #2: Yes

Reviewer #3: No

Reviewer #4: Yes

Reviewer #5: Yes

**********

6. Review Comments to the Author

Please use the space provided to explain your answers to the questions above. You may also include additional comments for the author, including concerns about dual publication, research ethics, or publication ethics. (Please upload your review as an attachment if it exceeds 20,000 characters)

Reviewer #1: The author clearly stated that the research was Explatory, meaning no actual world data available for comparison,

In my view, despite these deficiencies, the manuscript offers enrichment of the fraud knowledge.

It might be happed or it might be never happened, who knows

Reviewer #2: Summary of Main Strengths and Weaknesses

1. Main Strengths

• The topic is highly relevant and relatively new at the intersection of fraud studies, organisations, and complexity science.

• The integration of criminology literature, economic crime, and complex systems theory is quite rich and demonstrates the author's depth of theoretical understanding.

• The simulation design includes many scenarios and a variety of organisational sizes, with systematic reporting of scaling indicators.

• The simulation code and data are provided openly, supporting transparency and replication.

2 Main Weaknesses

• The model assumptions are overly simplified, particularly regarding the distribution of agent attributes and network structure, without adequate empirical justification and sensitivity testing.

• The lack of explicit model verification and validation procedures diminishes the strength of the claim that the discovered scaling patterns reflect generic fraud mechanisms in real organisations.

• The philosophical discussion and critique of the fraud triangle paradigm tend to be excessive, obscuring the article's concrete empirical and methodological contributions.

• There is an inconsistency between the acknowledgement of the model's significant limitations and the strong language used to describe the stability of the outcome patterns.

Reviewer #3: The manuscript addresses an important and timely topic, providing a novel application of agent-based modelling (ABM) to examine how organisational scale affects fraud dynamics. The study contributes to the literature by explicitly considering interactions between agents and how these generate emergent system-level behaviours, which is an underexplored perspective in organisational criminology. The use of simulation across multiple organisational sizes and behavioural regimes allows the identification of scaling laws that cannot be easily observed in empirical datasets, demonstrating the value of complexity-informed approaches in fraud research.

The theoretical grounding in complexity theory and scaling laws is well-articulated. The discussion of superlinear scaling of fraud and volatility with organisational size is compelling and consistent with prior findings in urban studies and crime science. The link between network connectivity, social influence, and the propagation of pro-fraud behaviour within organisations is clearly justified and operationalised through the ABM framework. The authors successfully show that larger organisations not only experience higher average fraud levels but also greater instability in fraud outcomes, highlighting the non-linear dynamics inherent in complex social systems.

Methodologically, the ABM is rigorously described, with clear specification of agent attributes, behavioural parameters, network scaling, and simulation protocols. The use of 9,000 simulation runs across nine organisational sizes and multiple behavioural regimes ensures robustness in the observed patterns. The log–log regression approach to estimating scaling exponents is appropriate and allows quantification of the degree to which fraud outcomes scale sublinearly, linearly, or superlinearly with organisational size. The inclusion of interaction terms between organisational size and behavioural parameters (q_avg and αₖ) is an important strength, demonstrating how social influence and network connectivity moderate fraud dynamics.

Despite these strengths, several points merit consideration. First, while the model clearly specifies the generative mechanism underlying fraud behaviour, the assumption of equal initial probabilities for motive, opportunity, and pro-fraud attitude may limit generalisability to real-world organisations. Second, the parameterisation of social influence (q_avg) and network connectivity scaling (αₖ) is scenario-based and not empirically calibrated, which should be emphasised as a limitation in the discussion. Third, peak fraud regressions excluded fourteen runs with zero values, which could introduce minor bias in scaling estimates; this exclusion should be discussed explicitly. Finally, although the study convincingly demonstrates system-level dynamics, the practical implications for organisational risk management and counter-fraud strategies could be expanded, particularly in linking observed scaling behaviours to actionable interventions.

In conclusion, this manuscript represents a significant contribution to understanding how organisational size and internal social dynamics shape fraud risk. The integration of ABM with complexity theory provides novel insights that are not attainable through conventional empirical approaches. Pending minor clarifications regarding parameter assumptions, treatment of zero-value runs, and discussion of practical implications, I recommend this manuscript for publication in PLOS ONE.

Reviewer #4: Dear Authors,

Thank you for the opportunity to read and review your research paper. I appreciate again the research idea, the strong theoretical background, the research methodology and the manner you presented and discussed the research results. I appreciate also the manner that you have adequately addressed reviewers' comments raised in a previous round of review and you feel that this manuscript is now acceptable for publication.

The manuscript technically sound, and the data support the conclusions.

The statistical analysis been performed appropriately and rigorously.

You made all data underlying the findings in their manuscript fully available.

The manuscript is presented in an intelligible fashion and written in standard English.

Kind regards,

The Anonymous Reviewer

Reviewer #5: Dear Authors,

I would like to inform you that the review of the revised manuscript has been completed. Based on my evaluation, the revisions have addressed the reviewer’s comments well and adequately.

Thank you for your efforts in revising the manuscript.

Sincerely,

Reviewer

**********

7. PLOS authors have the option to publish the peer review history of their article (what does this mean?). If published, this will include your full peer review and any attached files.

If you choose “no”, your identity will remain anonymous but your review may still be made public.

**Do you want your identity to be public for this peer review?**  For information about this choice, including consent withdrawal, please see our Privacy Policy.

Reviewer #1: **Yes:** Armanto Witjaksono

Reviewer #2: No

Reviewer #3: No

Reviewer #4: No

Reviewer #5: No

**********

[NOTE: If reviewer comments were submitted as an attachment file, they will be attached to this email and accessible via the submission site. Please log into your account, locate the manuscript record, and check for the action link "View Attachments". If this link does not appear, there are no attachment files.]

To ensure your figures meet our technical requirements, please review our figure guidelines: https://journals.plos.org/plosone/s/figures

You may also use PLOS’s free figure tool, NAAS, to help you prepare publication quality figures: https://journals.plos.org/plosone/s/figures#loc-tools-for-figure-preparation.

NAAS will assess whether your figures meet our technical requirements by comparing each figure against our figure specifications.

Attachment Submitted filename: Reviewer 5_Comments.docx

Attachment Submitted filename: REVIEW_PONE-D-25-65420_R1.pdf

# Author comment: Author response to Decision Letter 2

**DOI:** 10.1371/journal.pone.0342964.r004

14 Apr 2026

Response to editors and reviewers have been added as attachments

Attachment Submitted filename: 2026-04-12 Response to reviewers PLOS One Second Round.docx

# Peer review: Decision Letter 2

**DOI:** 10.1371/journal.pone.0342964.r005

**Authors:** Annesha Sil, Vanessa Carels, Daniel Parkes

30 Apr 2026

PONE-D-25-65420R2

Scaling of occupational fraud with organisational size: Evidence from an exploratory agent-based model

PLOS One

Dear Dr. Freeman,

Thank you for submitting your manuscript to PLOS ONE. After careful consideration, we feel that it has merit but does not fully meet PLOS ONE’s publication criteria as it currently stands. Therefore, we invite you to submit a revised version of the manuscript that addresses the points raised during the review process.

**Reviewer 2 has introduced some new comments. If you feel that these are unjustified, please ensure that you respond to each point and explain why. Reviewer 3 has some minor outstanding comments that need addressing.**

Please submit your revised manuscript by Jun 13 2026 11:59PM. If you will need more time than this to complete your revisions, please reply to this message or contact the journal office at plosone@plos.org. When you're ready to submit your revision, log on to https://www.editorialmanager.com/pone/ and select the 'Submissions Needing Revision' folder to locate your manuscript file.

Please include the following items when submitting your revised manuscript:

- A letter that responds to each point raised by the academic editor and reviewer(s). You should upload this letter as a separate file labeled 'Response to Reviewers'.
- A marked-up copy of your manuscript that highlights changes made to the original version. You should upload this as a separate file labeled 'Revised Manuscript with Track Changes'.
- An unmarked version of your revised paper without tracked changes. You should upload this as a separate file labeled 'Manuscript'.

If you would like to make changes to your financial disclosure, please include your updated statement in your cover letter. Guidelines for resubmitting your figure files are available below the reviewer comments at the end of this letter.

If applicable, we recommend that you deposit your laboratory protocols in protocols.io to enhance the reproducibility of your results. Protocols.io assigns your protocol its own identifier (DOI) so that it can be cited independently in the future. For instructions see: https://journals.plos.org/plosone/s/submission-guidelines#loc-laboratory-protocols. Additionally, PLOS ONE offers an option for publishing peer-reviewed Lab Protocol articles, which describe protocols hosted on protocols.io. Read more information on sharing protocols at https://plos.org/protocols?utm_medium=editorial-email&utm_source=authorletters&utm_campaign=protocols.

As the corresponding author, your ORCID iD is verified in the submission system and will appear in the published article. PLOS supports the use of ORCID, and we encourage all coauthors to register for an ORCID iD and use it as well. Please encourage your coauthors to verify their ORCID iD within the submission system before final acceptance, as unverified ORCID iDs will not appear in the published article. *Only*  the individual author can complete the verification step; PLOS staff *cannot*  verify ORCID iDs on behalf of authors.

We look forward to receiving your revised manuscript.

Kind regards,

Daniel Parkes, PhD

Staff Editor

PLOS One

**Journal Requirements:**

If the reviewer comments include a recommendation to cite specific previously published works, please review and evaluate these publications to determine whether they are relevant and should be cited. There is no requirement to cite these works unless the editor has indicated otherwise.

[Note: HTML markup is below. Please do not edit.]

Reviewers' comments:

Reviewer's Responses to Questions

**Comments to the Author**

1. If the authors have adequately addressed your comments raised in a previous round of review and you feel that this manuscript is now acceptable for publication, you may indicate that here to bypass the “Comments to the Author” section, enter your conflict of interest statement in the “Confidential to Editor” section, and submit your "Accept" recommendation.

Reviewer #2: (No Response)

Reviewer #3: All comments have been addressed

Reviewer #4: All comments have been addressed

**********

2. Is the manuscript technically sound, and do the data support the conclusions?

The manuscript must describe a technically sound piece of scientific research with data that supports the conclusions. Experiments must have been conducted rigorously, with appropriate controls, replication, and sample sizes. The conclusions must be drawn appropriately based on the data presented.

Reviewer #2: Partly

Reviewer #3: Yes

Reviewer #4: (No Response)

**********

3. Has the statistical analysis been performed appropriately and rigorously?

Reviewer #2: Yes

Reviewer #3: Yes

Reviewer #4: (No Response)

**********

4. Have the authors made all data underlying the findings in their manuscript fully available?

The PLOS Data policy requires authors to make all data underlying the findings described in their manuscript fully available without restriction, with rare exception (please refer to the Data Availability Statement in the manuscript PDF file). The data should be provided as part of the manuscript or its supporting information, or deposited to a public repository. For example, in addition to summary statistics, the data points behind means, medians and variance measures should be available. If there are restrictions on publicly sharing data—e.g. participant privacy or use of data from a third party—those must be specified.

Reviewer #2: Yes

Reviewer #3: Yes

Reviewer #4: Yes

**********

5. Is the manuscript presented in an intelligible fashion and written in standard English?

PLOS ONE does not copyedit accepted manuscripts, so the language in submitted articles must be clear, correct, and unambiguous. Any typographical or grammatical errors should be corrected at revision, so please note any specific errors here.

Reviewer #2: Yes

Reviewer #3: Yes

Reviewer #4: Yes

**********

6. Review Comments to the Author

Please use the space provided to explain your answers to the questions above. You may also include additional comments for the author, including concerns about dual publication, research ethics, or publication ethics. (Please upload your review as an attachment if it exceeds 20,000 characters)

**Reviewer #2:**  COMPREHENSIVE REVIEWER RECOMMENDATION

This research represents a significant theoretical contribution and a novel methodological approach in fraud criminology. The use of agent-based modelling to explore organization-level fraud dynamics is pioneering, and the findings on superlinear scaling patterns have theoretical and practical value. However, before publication in a tier-1 journal like PLOS ONE, several substantive issues require attention.

MAJOR REVISION RECOMMENDATIONS

1. Model Validation and Calibration (CRITICAL PRIORITY) Finding: The model operates in an empirical vacuum with parameter choices that are not calibrated against real organisational data.

Required Actions: - Conduct a systematic literature review of fraud surveys to extract empirical ranges for parameters such as fraud prevalence rates, susceptibility to influence, and network connectivity ratios. - Develop sensitivity analyses that comprehensively explore the parameter space and identify which scenarios produce outcomes consistent with empirical fraud data. - Create a validation matrix that compares simulated fraud patterns with patterns in empirical datasets (e.g., BIS fraud surveys and Kroll fraud reports). - Minimum Acceptable Outcome: Demonstrate that at least 70-80% of simulated scenarios produce fraud dynamics within empirically observable ranges (Carter, 2020). 2. Organisational Realism (High Priority) Finding: The model uses random networks that do not reflect real hierarchical, departmental, and power structures in organisations.

Required Actions: - Implement at least two alternative network structures: (a) hierarchical branching networks reflecting organisational charts and (b) small-world networks with community structure - Re-run the complete simulation suite with alternative network structures. - Compare findings to understand robustness in relation to network assumptions. - Discuss in the results how findings generalise or are specific to random network assumptions. (Carter, 2020) 3. Generalisability and Boundary Conditions (HIGH PRIORITY) Findings: Conclusions suggest general principles, but the boundaries of applicability are unclear.

Required Actions: - Create an explicit framework delineating (a) organisational types where findings are highly applicable, (b) organisational contexts requiring caution, and (c) organisational characteristics where predictions may fail. - Develop 2-3 empirical case studies or vignettes showing how findings apply in different real-world contexts - Discuss explicitly how findings generalise across industries, countries, organisational structures, levels of digitalisation 4. Actionability for Practitioners (MODERATE PRIORITY) Findings: Discussion provides theoretical insights but limited concrete guidance for fraud prevention practice.

Required Actions: - Develop a "Practitioner Implementation Guide" as supplementary material - Translate findings into specific, testable recommendations - Example: "Organisations expanding from 1,000 to 2,500 employees should anticipate fraud risk increasing by a factor of 2.0-2.4 and implement prevention infrastructure scaling at a minimum of 2.4x" - Create decision trees or matrices for risk managers to identify optimal prevention strategies given their organisational characteristics

MINOR REVISION RECOMMENDATIONS

1. Methodology Presentation Issues: - Model description scattered across the methodology section; lacks clear algorithm pseudocode or flowchart - Parameter values table incomplete; missing some parameter ranges and justifications Actions: - Create detailed model specification figure showing agent types, behaviours, decision rules - Provide complete parameter table with empirical sources, ranges explored, and sensitivity impact - Add pseudocode or UML diagram for key algorithms (social influence mechanism, fraud detection mechanism) 2. Writing Clarity Issues: Some passages are long and have multiple embedded clauses, reducing their readability. - Inconsistent terminology (e.g., "fraud agents" vs. "potential fraudsters") Actions: - Revise for more concise sentence structure - Standardise terminology across paper - Break complex paragraphs into multiple shorter paragraphs 3. Data Visualisation Issues: - Log-log plots in results difficult to interpret without additional context. - Missing visualisations of fraud dynamics over time (time series plots) - No illustration of model architecture or interaction structures Actions: - Enhance figures with insets showing linear-scale relationships for comparison - Add time series plots showing fraud trajectory in different organisational sizes - Create conceptual figure showing model structure, agent types, and interaction mechanisms - Ensure all figures are high resolution, with clear labels and comprehensive captions Data Visualisation Issues: - Log-log plots in results are difficult to interpret without additional context - Missing visualisations of fraud dynamics over time (time series plots) - No illustration of model architecture or interaction structures Actions: - Enhance figures with insets showing linear-scale relationships for comparison - Add time series plots showing fraud trajectory in different organisational sizes - Create a conceptual figure showing model structure, agent types, and interaction mechanisms - Ensure all figures have high resolution, clear labels, and comprehensive captions Literature Update Issues: - Some references are outdated; missing recent papers in fraud detection, complex systems modelling Actions: - Update the literature review with papers from 2022-2025 in: organisational fraud, complex systems criminology, agent-based models in social science - Add discussions of recent work on scaling laws in criminal networks, organisational resilience

SPECIFIC TECHNICAL RECOMMENDATIONS

Statistical Rigour 1. Confidence Intervals: Provide 95% confidence intervals for all estimated scaling exponents, not just point estimates. 2. Model Fit Statistics: Report adjusted R², residual diagnostics, and goodness-of-fit tests for power-law relationships 3. Multiple Comparisons: If conducting multiple hypothesis tests, apply appropriate corrections (Bonferroni or false discovery rate). 4. Effect Size: Report effect sizes, not just p-values, for increased interpretability. Reproducibility 1. Code Availability: Commit to depositing model code on the Open Science Framework or GitHub with complete documentation. 2. Data Availability: If using any datasets, provide clear statements about availability, licensing, and access procedures. 3. Random Seed: Report random seeds used for simulations for reproducibility. 4. Version Control: Document the NetLogo version used and any version-specific behaviours.

STRENGTHS TO MAINTAIN

1. Novel Theoretical Framework: Integrating complexity science with fraud criminology is innovative and valuable. 2. Rigorous Methodology: Agent-based modelling is an appropriate choice with a well-justified design. Confidence Intervals: Provide 95% confidence intervals for all estimated scaling exponents, not just point estimates. 2. Model Fit Statistics: Report R² adjusted, residual diagnostics, and goodness-of-fit tests for power-law relationships. 3. Multiple Comparisons: If conducting multiple hypothesis tests, apply appropriate corrections (Bonferroni or false discovery rate). 4. Effect Size: Report effect sizes, not just p-values, for increased interpretability. Reproducibility 1. Code Availability: Commit to depositing model code in the Open Science Framework or GitHub with complete documentation. 2. Data Availability: If using any datasets, provide clear statements about availability, licensing, and access procedures. 3. Random Seed: Report random seeds used for simulations for reproducibility. 4. Version Control: Document the NetLogo version used and any version-specific behaviours.

STRENGTHS TO MAINTAIN

1. Novel Theoretical Framework: Integrating complexity science with fraud criminology is innovative and valuable. 2. Rigorous Methodology: Agent-based modelling is an appropriate choice with a well-justified design. 3. Transparent Limitations: Authors are commendable in acknowledging model limitations. 4. Practical Relevance: Findings address real problems with significant organisational implications. 5. Clear Communication: Despite length, writing is generally clear with a logical structure. Transparent Limitations: Authors commendable in acknowledging model limitations 4. Practical Relevance: Findings address real problems with significant organisational implications. 5. Clear Communication: Despite length, writing generally clear with logical structure

**Reviewer #3:** In research, it's important to explain why previous findings vary. Is it due to limited empirical data or because they ignore systemic properties? This relates to your reasoning for choosing simulation.

Efficiency Issue (Point 2.14) requires improvements to the script by 15-20%. Please add at least one paragraph of theoretical discussion predicting how the simulation results would change if a small-world structure were applied (e.g., "We expect the superlinear effect to be stronger because clustering accelerates behavioral transmission").

**Reviewer #4:** Thank you for the opportunity to read and review your

research paper. I appreciate again the research idea, the

strong theoretical background, the research methodology

and the manner you presented and discussed the

research results. I appreciate also the manner that you

have adequately addressed reviewers' comments raised

in a previous round of review and you feel that this

manuscript is now acceptable for publication.

The manuscript technically sound, and the data support the conclusions.

The statistical analysis been performed appropriately and

rigorously.

You made all data underlying the findings in their

manuscript fully available.

The manuscript is presented in an intelligible fashion and

written in standard English.

**********

7. PLOS authors have the option to publish the peer review history of their article (what does this mean?). If published, this will include your full peer review and any attached files.

If you choose “no”, your identity will remain anonymous but your review may still be made public.

**Do you want your identity to be public for this peer review?**  For information about this choice, including consent withdrawal, please see our Privacy Policy.

Reviewer #2: No

Reviewer #3: No

Reviewer #4: **Yes:** Ioan-Bogdan ROBU

**********

[NOTE: If reviewer comments were submitted as an attachment file, they will be attached to this email and accessible via the submission site. Please log into your account, locate the manuscript record, and check for the action link "View Attachments". If this link does not appear, there are no attachment files.]

To ensure your figures meet our technical requirements, please review our figure guidelines: https://journals.plos.org/plosone/s/figures

You may also use PLOS’s free figure tool, NAAS, to help you prepare publication quality figures: https://journals.plos.org/plosone/s/figures#loc-tools-for-figure-preparation.

NAAS will assess whether your figures meet our technical requirements by comparing each figure against our figure specifications.

Attachment Submitted filename: ARTICLE - PONE-D-25-65420_R2.pdf

# Author comment: Author response to Decision Letter 3

**DOI:** 10.1371/journal.pone.0342964.r006

12 May 2026

A comprehensive response to reviewer comments has been attached.

Attachment Submitted filename: 2026-05-11 Response to reviewers PLOS One.docx

# Peer review: Decision Letter 3

**DOI:** 10.1371/journal.pone.0342964.r007

**Authors:** Annesha Sil, Vanessa Carels, Daniel Parkes, Hadi Hussain

25 May 2026

PONE-D-25-65420R3

Scaling of occupational fraud with organisational size: Evidence from an exploratory agent-based model

PLOS One

Dear Dr. Freeman,

Thank you for submitting your manuscript to PLOS ONE. After careful consideration, we feel that it has merit but does not fully meet PLOS ONE’s publication criteria as it currently stands. Therefore, we invite you to submit a revised version of the manuscript that addresses the points raised during the review process.

Please submit your revised manuscript by Jul 09 2026 11:59PM. If you will need more time than this to complete your revisions, please reply to this message or contact the journal office at plosone@plos.org. When you're ready to submit your revision, log on to https://www.editorialmanager.com/pone/ and select the 'Submissions Needing Revision' folder to locate your manuscript file.

Please include the following items when submitting your revised manuscript:

- A letter that responds to each point raised by the academic editor and reviewer(s). You should upload this letter as a separate file labeled 'Response to Reviewers'.
- A marked-up copy of your manuscript that highlights changes made to the original version. You should upload this as a separate file labeled 'Revised Manuscript with Track Changes'.
- An unmarked version of your revised paper without tracked changes. You should upload this as a separate file labeled 'Manuscript'.

If you would like to make changes to your financial disclosure, please include your updated statement in your cover letter. Guidelines for resubmitting your figure files are available below the reviewer comments at the end of this letter.

If applicable, we recommend that you deposit your laboratory protocols in protocols.io to enhance the reproducibility of your results. Protocols.io assigns your protocol its own identifier (DOI) so that it can be cited independently in the future. For instructions see: https://journals.plos.org/plosone/s/submission-guidelines#loc-laboratory-protocols. Additionally, PLOS ONE offers an option for publishing peer-reviewed Lab Protocol articles, which describe protocols hosted on protocols.io. Read more information on sharing protocols at https://plos.org/protocols?utm_medium=editorial-email&utm_source=authorletters&utm_campaign=protocols.

As the corresponding author, your ORCID iD is verified in the submission system and will appear in the published article. PLOS supports the use of ORCID, and we encourage all coauthors to register for an ORCID iD and use it as well. Please encourage your coauthors to verify their ORCID iD within the submission system before final acceptance, as unverified ORCID iDs will not appear in the published article. *Only*  the individual author can complete the verification step; PLOS staff *cannot*  verify ORCID iDs on behalf of authors.

We look forward to receiving your revised manuscript.

Kind regards,

Hadi Hussain

Academic Editor

PLOS One

Journal Requirements:

If the reviewer comments include a recommendation to cite specific previously published works, please review and evaluate these publications to determine whether they are relevant and should be cited. There is no requirement to cite these works unless the editor has indicated otherwise.

Please review your reference list to ensure that it is complete and correct. If you have cited papers that have been retracted, please include the rationale for doing so in the manuscript text, or remove these references and replace them with relevant current references. Any changes to the reference list should be mentioned in the rebuttal letter that accompanies your revised manuscript. If you need to cite a retracted article, indicate the article’s retracted status in the References list and also include a citation and full reference for the retraction notice.

[Note: HTML markup is below. Please do not edit.]

Reviewers' comments:

Reviewer's Responses to Questions

**Comments to the Author**

1. If the authors have adequately addressed your comments raised in a previous round of review and you feel that this manuscript is now acceptable for publication, you may indicate that here to bypass the “Comments to the Author” section, enter your conflict of interest statement in the “Confidential to Editor” section, and submit your "Accept" recommendation.

Reviewer #2: (No Response)

Reviewer #4: All comments have been addressed

**********

2. Is the manuscript technically sound, and do the data support the conclusions?

The manuscript must describe a technically sound piece of scientific research with data that supports the conclusions. Experiments must have been conducted rigorously, with appropriate controls, replication, and sample sizes. The conclusions must be drawn appropriately based on the data presented.

Reviewer #2: Yes

Reviewer #4: Yes

**********

3. Has the statistical analysis been performed appropriately and rigorously?

Reviewer #2: Yes

Reviewer #4: Yes

**********

4. Have the authors made all data underlying the findings in their manuscript fully available?

The PLOS Data policy requires authors to make all data underlying the findings described in their manuscript fully available without restriction, with rare exception (please refer to the Data Availability Statement in the manuscript PDF file). The data should be provided as part of the manuscript or its supporting information, or deposited to a public repository. For example, in addition to summary statistics, the data points behind means, medians and variance measures should be available. If there are restrictions on publicly sharing data—e.g. participant privacy or use of data from a third party—those must be specified.

Reviewer #2: (No Response)

Reviewer #4: Yes

**********

5. Is the manuscript presented in an intelligible fashion and written in standard English?

PLOS ONE does not copyedit accepted manuscripts, so the language in submitted articles must be clear, correct, and unambiguous. Any typographical or grammatical errors should be corrected at revision, so please note any specific errors here.

Reviewer #2: Yes

Reviewer #4: Yes

**********

6. Review Comments to the Author

Please use the space provided to explain your answers to the questions above. You may also include additional comments for the author, including concerns about dual publication, research ethics, or publication ethics. (Please upload your review as an attachment if it exceeds 20,000 characters)

Reviewer #2: Style and Readability Notes

Although the manuscript is in English, some issues of readability and consistency of terminology are apparent.

Weakness

• There are many long sentences with multiple clauses that can reduce clarity, especially for cross-disciplinary readers.

• Some terms do not appear to be entirely consistent throughout the text (e.g., the terms for perpetrators: “agents”, “employees”, “fraudulent agents”), which may confuse non-technical readers.

• Paragraphs tend to be long; these “blocks” of text make it difficult for readers to identify the main point of each paragraph.

Improvement Suggestions

• Make thorough style edits to:

o Breaking down long compound sentences into 2–3 shorter sentences with a clear subject–predicate–object–description structure;

o Reduce nonessential subordinate clauses or move explanatory clauses to separate sentences.

• Standardize key terminology, for example:

Use “agents (employees)” on the first mention, then consistently use “agents” or “employees” as the context requires.

Use consistent terms for fraud status (for example, “fraudulent agents” rather than changing terms).

• Review very long paragraphs and break them into shorter units, each with a single main idea. This will significantly improve readability in top-tier journals with a cross-disciplinary readership.

However, to meet the standards of a top-tier journal, authors are advised to:

1. Streamline the narrative focus and reduce redundancy, especially in the introduction, literature review, discussion, and conclusion.

2. Clarifying theoretical structures and conceptual expectations before results, including through the formulation of propositions.

3. Strengthen reporting of ABM methodology and statistical analysis, including parameter justification and (where possible) additional sensitivity analysis.

4. Improve readability with more concise sentences, shorter paragraphs, and consistent terminology.

With substantial revisions to these aspects, the manuscript has strong potential to meet the quality expectations of top-tier journals in the fields of economic criminology and organizational complexity studies.

Reviewer #4: (No Response)

**********

7. PLOS authors have the option to publish the peer review history of their article (what does this mean?). If published, this will include your full peer review and any attached files.

If you choose “no”, your identity will remain anonymous but your review may still be made public.

**Do you want your identity to be public for this peer review?**  For information about this choice, including consent withdrawal, please see our Privacy Policy.

Reviewer #2: No

Reviewer #4: No

**********

[NOTE: If reviewer comments were submitted as an attachment file, they will be attached to this email and accessible via the submission site. Please log into your account, locate the manuscript record, and check for the action link "View Attachments". If this link does not appear, there are no attachment files.]

To ensure your figures meet our technical requirements, please review our figure guidelines: https://journals.plos.org/plosone/s/figures

You may also use PLOS’s free figure tool, NAAS, to help you prepare publication quality figures: https://journals.plos.org/plosone/s/figures#loc-tools-for-figure-preparation.

NAAS will assess whether your figures meet our technical requirements by comparing each figure against our figure specifications.

Attachment Submitted filename: ROUND 3_14 Mei PONE-D-25-65420_R3.pdf

# Author comment: Author response to Decision Letter 4

**DOI:** 10.1371/journal.pone.0342964.r008

7 Jun 2026

A letter to the editor and a separate file containing responses to all reviewer comments have been uploaded.

Attachment Submitted filename: 2026-06-07 Response to reviewers PLOS One Fourth Round.docx

# Editor report: Decision Letter 4

**DOI:** 10.1371/journal.pone.0342964.r009

**Authors:** Annesha Sil, Vanessa Carels, Daniel Parkes, Hadi Hussain, Hadi Hussain

3 Jul 2026

Organisational Scale and Fraud Volatility: An Exploratory Agent-Based Simulation of Occupational Fraud Dynamics

PONE-D-25-65420R4

Dear Dr. Freeman,

We’re pleased to inform you that your manuscript has been judged scientifically suitable for publication and will be formally accepted for publication once it meets all outstanding technical requirements.

Within one week, you’ll receive an e-mail detailing the required amendments. When these have been addressed, you’ll receive a formal acceptance letter and your manuscript will be scheduled for publication.

An invoice will be generated when your article is formally accepted. Please note, if your institution has a publishing partnership with PLOS and your article meets the relevant criteria, all or part of your publication costs will be covered. Please make sure your user information is up-to-date by logging into Editorial Manager at Editorial Manager® and clicking the ‘Update My Information' link at the top of the page. For questions related to billing, please contact billing support.

If your institution or institutions have a press office, please notify them about your upcoming paper to help maximize its impact. If they’ll be preparing press materials, please inform our press team as soon as possible -- no later than 48 hours after receiving the formal acceptance. Your manuscript will remain under strict press embargo until 2 pm Eastern Time on the date of publication. For more information, please contact onepress@plos.org.

Kind regards,

Hadi Hussain

Academic Editor

PLOS One

Additional Editor Comments (optional):

Reviewers' comments:

# Editor report: Acceptance letter

**DOI:** 10.1371/journal.pone.0342964.r010

**Authors:** Annesha Sil, Vanessa Carels, Daniel Parkes, Hadi Hussain, Hadi Hussain

PONE-D-25-65420R4

PLOS One

Dear Dr. Freeman,

I'm pleased to inform you that your manuscript has been deemed suitable for publication in PLOS One. Congratulations! Your manuscript is now being handed over to our production team.

At this stage, our production department will prepare your paper for publication. This includes ensuring the following:

* All references, tables, and figures are properly cited

* All relevant supporting information is included in the manuscript submission,

* There are no issues that prevent the paper from being properly typeset

You will receive further instructions from the production team, including instructions on how to review your proof when it is ready. Please keep in mind that we are working through a large volume of accepted articles, so please give us a few days to review your paper and let you know the next and final steps.

Lastly, if your institution or institutions have a press office, please let them know about your upcoming paper now to help maximize its impact. If they'll be preparing press materials, please inform our press team within the next 48 hours. Your manuscript will remain under strict press embargo until 2 pm Eastern Time on the date of publication. For more information, please contact onepress@plos.org.

You will receive an invoice from PLOS for your publication fee after your manuscript has reached the completed accept phase. If you receive an email requesting payment before acceptance or for any other service, this may be a phishing scheme. Learn how to identify phishing emails and protect your accounts at https://explore.plos.org/phishing.

If we can help with anything else, please email us at customercare@plos.org.

Thank you for submitting your work to PLOS ONE and supporting open access.

Kind regards,

PLOS ONE Editorial Office Staff

on behalf of

Dr. Hadi Hussain

Academic Editor

PLOS One
